# Supplementary material for: Genome-wide identification of xyloglucan endotransglucosylase/hydrolase gene family members in peanut and their expression profiles during seed germination
Source: PeerJ. 2022 May 17;10:e13428. doi: 10.7717/peerj.13428 (PMC9121870; doi:10.7717/peerj.13428)
Supplement: Supplemental Information 3 [file peerj-10-13428-s003.docx]

**Supplementary File 3 Coding sequences of Peanut *AhXTHs*.**

>*AhXTH1*

ATGGCTCCTTCATGGGGTGGTTCATATTTTCTCTTTCTTTGTATGTTGTTAGGGTTCAGCACAATTGCATATGGTGGGAATTTCAGCACTGATTTTGACCTTCTATTTGGAGATGATAGGGTTAAGATTTCTGATGGTGGACAAAGCATGTCCCTTGCAATGGATAAATATTCTGGTTCTGGGGTTGCTACCAAGGATCAATTCTTGTTTGGAAGATTTGACATGAAGATCAAGCTTGTTGGAGGAAACTCTGCAGGAACTGTTACTGCATTTTATCTAAGCTCCACAGGAGATCACCATGATGAGGTTGATCTTGAGTTCTTGGGGAACTTAACCGGAGATCCATATATGCTATCAACAAATGTGTTTGCAAATGGTGTTGGGGGTCGTGAGGTTCAATACTATCTTTGGTTTGATCCAACAGCAGATTATCACACCTATTCCATTGATTGGAGCCCTACCCGCATAATACTCTGGGTGGACGACACTCCAATCAGAGTGATAAACAACAAAGAAATAATCGGTGTTCCATTCCCAACAAAGCAACCAATGAGGCTGTACACAACACTCTGGAATGGGGATGCATGGGCAACAAGATGGGGCCAAGTGAAGATTGACTTGTCACAAGCACCATTTATAGCAAGGTTCAAGAACTACAATGGCACTGCTTGTGTTCCAAAGAAAGGCATAGCAGATTGCAAGGGTTTTGGTGCTTCAATGAAGAGAGGTCTTGACAATGAAAACAAGAAAAAGTTGAAGCAAGTTAACTCAAAGTGGGTTGTTTATCACTATTGCCGTGATTTGAGGCGTTATGCTCATGGATTACCCTTTGAATGCCGTAGGGACAACATGGCACACAGTGATAATAATCAATAA

>*AhXTH2*

ATGGCCACCTTTTATCCTCTCATTAGAAATGGTGGTGGTTCATTATTTCTAATACTGCTATCATGGGTTTTCCTAGTATCTTCTTTATTATGTGTTTTGGGACGACCAGCCACTTTTGAGCAAGACTTTAGAGCCACGTGGTCTGAGTCTCATATCAGGCATACTGATCAAGGCAGAACAATCCAACTCATGCTCGACCGAAGCTCCGGATGTGGGTTTGCTTCCAAGGTGAAGTACATGTTTGGGCGTGTTAGCATGAAGATCAAACTCGTCCCTGGAGACTCTGCTGGCACCGTCACCGCATTTTACATGAATTCTGATACAGATTCTGTTCGTGATGAGCTGGACTTCGAGTTCTTGGGGAACAGGAGTGGACAGCCATACACGGTTCAAACAAACGTATATGCTCATGGAAAGGGTGATAGAGAGCAAAGGGTCAACCTCTGGTTTGATCCTTCTGCTGATTTCCATACCTACACTATTCTCTGGAACCATAAACATATTGTATTTTCCGTGGACGATTTCCCCATCAGAGTATACAAGAACAACGAAGCGAAAGGGGTGCCATACCCAAAGATGCAAGCCATGGGAGTGTACTCAACACTATGGGAAGCAGATAACTGGGCAACAAGAGGTGGGTTAGAGAAAATCGATTGGAGCAAAGCACCCTTCAATGCCTACTACAAAGACTTCGACATTGAAGGGTGTTCAGTTCCGGGTCCGGCAAGCTGCGCCTCCAACCCGAGTAACTGGTGGGAAGGCGCAGCCTACCAAGAACTTAGTGCCATTCAAGCAAGGAGGTACAGATGGGTTCGTATTAACCACGTTGTCTATGACTATTGTCAAGATAAGTCAAGGTTCCCAGTCACCCCACCTGAGTGTCTTGCTGGCATTTGA

*>AhXTH3*

ATGCACATCATCGATCACTCATATACTTCTCTTGGCTTCTTTTGCTTCCTTCTCTCTCTCTATACATATTTGCCTCCTATTTTCCAACCAAATCAAATTAAGGGAACCATAATTAATATGGGGATTGAGGTACTTATTATTAATATTGTTCTCCTTATAGGGTTTGCGGTTGCTGCATCCGCAGGTAACTTCAACCAAGACTTTGAAATTACATGGGGTAATGACCGTGCCAAAGTCCTCAACAACGGACAGCTTCTGACGCTGTCCCTTGACAAGGCCTCCGGCTCCGGCTTCCGTTCCCGGAACGAGTACCTGTTTGGAAAGATCGACATGCAGCTTAAACTTGTTCCTGGTAATTCAGCAGGCACAGTCACAGCCTATTATCTAAGTTCACTTGGTGACACTCACGATGAGATAGACTTTGAATTTCTGGGGAACTTGAGTGGTGAACCATACACTCTGCACACGAATGTATTCACGCAAGGGAAAGGCAACAGAGAACAACAGTTCCATCTATGGTTCGACCCCACCAAGGACTTCCATACGTATTCTCTTCTTTGGAATCCTCAAAGCATCATATTCTCTGTGGACGGCACACCAATAAGGGAGTTCAAGAATTGGGAATCAAAAGGAGTTCCATTTCCCAAGAATCAAGCAATGAGGATATACTCAAGTCTATGGGACGCAGAAGATTGGGCCACAAGGGGTGGGCTTGTGAAGACTGATTGGACCCAAGCCCCATTCACAGCCTCATACAAAGGCTTCAATGCACAAGCTTGTGTTTGGACCTCTTCTTCAGGCTCCTCTTGTTCTTCAAAACAAGGCCAATCATGGTTCACTCAGTCACTTGACTCAACTGGGCAAGCAAGAATCCAGTGGGTTCAGAAGAACTACATGATCTACAATTACTGCACTGATACCAAACGATTCCCACAAGGCCTTCCTCCTGAATGCACCCTTGCATGA

*>AhXTH4*

ATGGCTGCTGCTTGTGTGAACAATAATAATGATGCATTGATTGTGATTGTCATAACATTGTTCCTAATAATCACTTTGCCTTCTTCTTCTATGGCTGGTAGCAATTTCAACCAACAAGTTGATATCACTTGGGGCGACGGTAGAGGTAAGATTCTCAATAGTGGCAAGATTCTTACCCTGTCTCTAGACAGAGCTTCTGGCTCTGGCTTTCAATCCAAGAATGAGTATCTGTATGGCAAGATTGACATGCAAATCAAGCTTGTCCCTGGAAATTCTGCTGGCACTGTCACTGCTTATTACTTACGTTCAGATGGAATATCATGGGATGAGATAGACTTTGAGTTCTTGGGAAATCTTAGTGGTGATCCGTACGTAGTTCATACAAATGTGTATACACAAGGAAAAGGTGGCAAAGAGCAACAATTTTATCTCTGGTTTGATCCAACCGCAAATCTTCACACCTATTCCATTCTCTGGAATCCTGCACACATTGTGTTTTATATTGATGGAAGACCAATAAGGGAGTTCAAAAACTTAGAGGGTGTTGGTGTTTCCTACCCAAAGAACCAACCAATGAAACTGTATTCAAGTATCTGGAACGCTGATGATTGGGCAACAAGAGGAGGGCTTGTGAAGACAGATTGGAGCCAAGCTCCATTCACAGCTTCCTTCAAGAACTTGAAAGCCAATGGTTGTGTTTGGTCCAATGGTGTTTCTTCATGCAACTTATCATCATCAAATTCCTCTAATAATAACAACTCTTGGCTTTCTCAACAGCTTGATTCAAACGGTCAGAGGAAGCTCAAATGGGTGCAGAAGAATTACATGATTTATAATTACTGCTCAGACATTAACCGATTCCCTCAGGGCCTTCCTCTTGAATGCACGCTCCGAACCACCTGA

*>AhXTH5*

ATGCTTCTGCCTTATCCCACTAGTGTTGTTATGGTGGCACTATTCACTATGACGATGATGATGATGTTGTGCGGTGGCGATTTGCATAAGAACATTGACATAACATGGGGTAACGGGCGTGCCCAGATGCTCAACAATGGCCAGCTTCTTACTGTGTCCCTTGACGCTGCCTCTGGCTCTGGATTCCAATCCAAGGATCACTATCTTTTTGGCCACTTTCAAATTCAACTCAAACTTGTTCCTGGAAATTCTGCAGGCACTGTCACTTCTTTCTATTTACAATCAGAAGGATCAACTTGGGATGAAATAGACTTTGAATTCCTTGGAAATCTGAGTGGCAACCCGTATATTCTTCACTCTAATGTAATCACGCAAGGTAAAGGTGGCAGGGAGCAACAATTCTATCTATGGTTTGATCCCACTTCTGATTTTCACACTTACTCAATTCTGTGGAATCCACTATGCATCATAGAATTCAAGAACTATGAATCAAGGAATATATCATTTCCAATGAAGAAACCAATGAGAATGTATGGAAGCTTATGGGATGCTGAAGATTGGGCTACAAGGAGTGGTCTAATCAAGACAAATTGGAGTGCAGCTCCATTCATTGCATATTTTAAAAATTTTTTTGTCAATGCTTGTGTTGAGTCTACTCCTCCTTCTATTACGTCTTCTTGCACACACCCAAACAAAAAGAGTAATAATCATAATTCCGTTGGTGAAAAATGGATCACACAAGGGTTGAAGCCCTCTGAAGTAGACAAACTCAACTGGGTGCACAAGAATTTCATAGTCTATAACTATTGTTCCGACTTAAAACGCTTCCCTCAAGGATTGCCGCTAGAATGTAAAATAAACTAA

*>AhXTH6*

ATGGCTGCTTCAACTCTCTCCTACTTGCTTCTAATCCCTCTTCTGATGGTTGTTGCATATGCTGGTAACTTAGACCAAGAATTTGACATTACATGGGGCGATGGACGAGCCAAGATGCTCAACAATGGGGAGCTACTCACATTGTCACTTGACAAAGCCTCTGGTTCTGGGTTCCAATCCAAGAACGAGTATCTCTTTGGCAAGATTGACATGCAGCTCAAGCTTGTTCCTGGGAACTCTGCTGGCACTGTAACTGCCTATTATTTATCTTCAAAAGGAACAACTTGGGATGAGATAGACTATGAATTCCTTGGGAATTTGAGCGGAGATCCATACATTCTTCACACCAATGTTTTCAGCCAAGGCAAAGGAAACAGGGAACAACAATTCTATCTATGGTTTGATCCTACTGCTGATTTCCACACTTACTCCATCACTTGGAACCCCCAAAGGATTATATTTTCAGTGGATGGAACTCCAATCAGAGAGTTCAAGAACTCAGAGAGCATGGGTGTCCCCTTCCCAAAGAGCCAGCCAATGAGAATATATTCAAGTTTGTGGAATGCAGATGACTGGGCAACAAGAGGTGGACTTGTGAAGACAGATTGGACAAAAGCTCCATTCACAGCTTCATACAGGAACTTCAATGCTGATGCTTGCATATGGTCTAACGGGGCTTCATCTTGTGGTTCTGGTTCAGGTTCTTCCTCTACTAGCAGTTCATGGCTTTCACAAGAGTTGGACACTACGGCACAAGAGAGGCTAAGATGGGTGCAGAAGAACTACATGATATACAATTACTGCACAGATGCAAAGAGGTTTCCACAGGGTTTTCCTCCAGAATGCAGAACCTCTTAA

*>AhXTH7*

ATGGCGGATCCAGTTCTTCACCCAGACACTACAAACCCTCTTCATCACCATCAAACACAACCTCTCAAAGAGATCGCCATTGACTACACACCAGAAGCATGCTCTCACTGCCCTAATTCCAACACCATCACCCTCACCTTCGATCACCGCGGCGGCGCCAGGTGGCGCACCACCACTCGCTTCCACTATGGCACCTTCAGCTCCCTCATCCAGTGCCCTAAGGGTAACACCAATGGCCTCAACTTCAACCTCTACCTCTCTTCCTTAGAAGGTGAAAAGTCCCAAGATGAGATAGATTTTGAGTTCTTGGGTAAGGATAGGACCATTGTGCAGACAAACTACTTCAGTGGTGGCAATGGCAACAAGGAGAAGATTCACCATCTGGGTTTTGATGCCTCTGATGGTTTTCATGAGTATGTGATCAAGTGGAGTTATGATGTGATAGAGTGGCTCATTGATGGGAAGGTAGTCAGAAGAGAGGAGAAGAAAGAAGGGAAAGGGTTCCCTCAGAAGCCTATGTTCTTGTATGCTTCCATTTGGGATGCAAGCTGCATTGATAATGGGAGGTGGGCAGGGAAGTATGATGGGAGTGATGCACCTTATGTTTGTCTCTATAAAGACATTCATGTCCCTACTAGCACTGCAGTTAAATGA

*>AhXTH8*

ATGATGAAGGCTCTACTATTATTTCTCTTTGCTATTTCTTCATCACTGTTTATGGAACCTTGTTATTCAAGCAGTGGATATTGGCCTCCTTCACCAGGATACTGGCCAAGTCACAAATTCAGGTCTATGAACTTTTACAAAGGATTTAGAAACCTTTGGGGTCCTCAACACCAAGCACTTGACAACAATAATGCATTAACAATTTGGCTTGATAGAACCTCAGGGAGTGGATTCAAATCAGTTCGTCCATTTAGATCAGGTTACTTTGGTGCTTCAATTAAGCTCCACCCTGGCTACACTGCAGGAGTTATAACAGCTTTCTATCTTTCTAACAATGAAGCACACCCTGGGTTCCATGATGAAGTGGACATAGAGTTTCTTGGGACCACATTTGGAAAACCTTATACTTTACAAACAAATGTTTACATAAGAGGAAGTGGGGATGGAACGATTATAGGAAGAGAGATGAAGTTCCATTTGTGGTTTGATCCTACCAAAGATTTTCATCACTATGCTATTCTTTGGTCTCCTAAAGAAATCATACCAATGTGGGTTTATGGTTCAATATGGGATGCATCATCATGGGCAACTGAAGATGGCAAGTACAAAGCTGATTATAGGTACCAACCTTTTGTTGCAAGGTACACAAATTTCAAGGCTAGTGGTTGCTCAGCCTATGCATCACGGTGGTGCCACCCAGTCTCAGCCTCACCATATAGGTCCGGTGGCTTGACCAGGCAACAATATTGGGCCATGAGGTGGGTCCAAAGACACCATATGGTTTATAACTATTGCCAAGACCCCAAAAGGGACCATAGATTAACACCTGAATGTTGGGGTAAAAAAAAGGAAAATAATGAAAAGAGAATGAGAAGCAGAGGGTATGTAATTGTGCATATATATGCTAGTTACTTCATTATATTATTGTTGACTGTTAGTTCTGAAACTTTTCAATTGGTTTCTTTTATTTCATTTACTCCAAGGAACCTTCAAGTGCAAAATTAA

*>AhXTH9*

ATGTTCACTACAATGCTGCCATCATCACCCCTTTCTTTCTTCTTCTTCTTCTTCCTTCTTGTACTCTCATTCATGATATGTGCTAGTGCTCAGGGTCCACCTTCACCTGGCTACTACCCTGGTTCCAAAATTAGTCCTATTAGCTTTGATCAAGGGTTCAGAAACCTATGGGGACCTCAGCATCAGAGGCTAGAACAAGGCACATTATCAATATGGCTAGATTCTAACTCGGGGAGTGGATTCAAGTCACTTCACTCTTATCAATCTGGATACTTTAGTGCTGCAATTAAGCTTCAACCCGGTTATACTGCAGGGGTCATTACAACTCTTTATCTTTCAAACAACCAAGATCACCCCGGAAATCACGACGAAATCGACATTGAGTTCCTAGGTACCACCCCTGATAAGCCATATGTTCTGCAGACAAATGTATACATAAGAGGAACTGGTGATGGCAACATTGTAGGGAGAGAGATGAGGTTTCATCTTTGGTTTGATCCAACACAAGATTTTCACATCTATGCTATTCTATGGAAACCAAGTGAGACAATATTTTTTGTGGATGATGTTCCCATAAGGAGGTACCCTAGGAAAGGTGATGCTACATACCCAAATAGACCAATGTATGTGTATGGATCAATATGGGATGCATCTTCATGGGCAACAGAAGATGGAAAATACAAAGCAAATTATAAATACCAACCCTTCATTGGAAGGTACAAGAACTTCAAGCTCCAAGGTTGCACCATTAATGAAAGCCCTTCCTCATGCAAGCCACCATCTGCATCACCATCAGGGTATGGAAGTCTTAGTCTTCAGCAAATTTCAGCCATGCAATGGGTCCAAAACCACTACTTGGTCTATTATTATTGCCATGACCCTAAGAGAGATCATACTCTTACCCCAGAGTGCTAG

*>AhXTH10*

ATGTCTAAGGAGGTGTCGTTGTTTTTGGGATTGGTTATGGGGTTTGTGTTTGTTGGAGTGGCTTTTGCTGCTGCCACTGCCAAGTTTGAAGAACTCTTCCAACCAAGTTGGGCTTTAGACCATTTCATTCATGAAGGAGACCTTCTCAAACTCAAGCTTGATAACTATTCCGGTGCTGGGTTTGTATCCAAAAGCAAGTATATGTTTGGGAAAGTTACCGTCCAACTTAAACTTGTTGAAGGTGATTCTGCCGGAACAGTTACTGCTTTCTATATGTCATCGGAGGGTCCAAATCACAACGAGTTTGATTTTGAGTTCCTGGGGAACACCACAGGGGAACCTTACTCTGTTCAAACAAATGTGTATGTGAATGGAGTGGGAAATAGAGAGCAAAGGCTAAACCTTTGGTTTGACCCTACTAAAGACTTTCATTCTTACTCTTTCTTTTGGAACCAACGTCAAGTTATATTTCTAGTGGATGAAACACCAATAAGGGTGCACACAAACATGGAACACAGGGGTATTCCATTTCCAAAGGATCAAGCAATGGGTGTATATAGCTCAATTTGGAACGCTGATGATTGGGCCACACAAGGTGGAAGAGTGAAAACTAATTGGAGCCATGCACCTTTCATTGCAACATATAAGGCCTTTGAGATCAATGGTTGTGAGTGTCCAATAGTGTCATCAACATCAGTGGAAAATTTGAAGAGGTGTAGTAGTAATGAGAAGAAGTATTGGTGGGATGAGCCTAATTTGGGTGTGTTAAGTTTGCATCAAAGTCACCAACTTATGTGGGTTAGGGCCAAACATATGGTTTATGATTATTGTGCTGATACTGCTAGGTTCCCTGTTATGCCTGCTGAGTGTGTTCATCATAGTCACCACAAATTAGTGCTAAAAAATTAG

*>AhXTH11*

ATGACAACACTAGTAACTAAGGTCCCTATTGGGTTTCTTTCTCTTCTCTTAATCATTACTATAGCTACCAAAGCTGCGGCTGGTAACTTCTATCAAGACTTTGAAGTAACATGGGGTGATAACCGTGCTAAGATCTTTAACGGTGGCCAGCTTCTTACACTGTCCTTGGACAGAGCCTCCGGCTCCGGATTTCGCTCCAAGAACGAGTATTTGTTTGCCAAACTTGACATGCAAATCAAACTCGTTCCTGGTAACTCCGCTGGCACCGTTACAACATACTATCTATCTTCGTCGGGTGGGACACATGACGAAATAGACTTTGAATTTCTGGGGAACTTGAGTGGAGATCCGTACATTCTTCACACAAACGTATTCACGCAAGGGAAAGGCAACAGAGAACAGCAGTTCTATCTATGGTTCGACCCCACCGCCGACTTCCACACCTATTCCATTCTTTGGAATCCTCACAACATCATATTCTCTGTGGACGGCACACCAATAAGGGAGTTCAAGAATTTAGAATCAAGAGGGGTCCCATTCCCAAAGAACCAACCCATGAGACTATACTCAAGCCTTTGGAATGCTGATGACTGGGCCACAAGGGGTGGGCTTGTGAAGACAGATTGGGCCCATGCCCCATTCACTGCCTCATACAGAAACTTCAATGCACCACCAGCCCAATCATGGATGGGCCAATCTCTTGATTCAACAGGCCTTGCAAGAATCCATTGGGTCCAAAGGAATTACATGATTTACAATTATTGCACTGATCTCAAACGCTTCCCACAAGGGCCTCCTCTTGAATGCTCACTTGCATGA

*>AhXTH12*

ATGGCACTAATTTGGCAACTCAAGCTTCTCTTCTCACCTCTCTTAATAATCTTATTAGCAAACGTGGTTTCTTCACGCACTAGGCCCTTCACAGCTCCAACTGTGACACCCTTGACTAATTCCTTCCCTCGTGTCCCAATTGATCCTAATTTTTCTAATGCTTTTGGGGCCTCCAATATCAAGCTTCTTGCTAATGGGACCATGGCCACTCTGGCTCTCGACAAACTCTCAGGCTCCGGATTAGTGTCCAAAAGCAGTTACTACTATGGATTCTTCAGTGCTGCAATCAAATTACCAGCTGGTATATCATCTGGAGTTGTAGTTGCCTTCTATTTGTCTAATGCAGATAAATTCCCTCATAACCATGATGAGATTGATATCGAACTTCTTGGGCATGACAAGAGGAACGATTGGGTTATCCAAACAAATGTGTATGCCAATGGAAGCGTGAGCACTGGAAGAGAAGAAAAGTTCTACTTCTGGTTCGACCCGACAAAGCAGTACCATTACTATAGCATCTTGTGGAACAGTTATCATACAGTGTTCCTAGTGGACAACATTCCAGTGAGGGAATTCATACACGGGTCTGTTTTCCCATCAAAACCGATGTCGGTGTATGCAACAATATGGGATGGTTCAGAATGGGCCACACATGGTGGAAAATACCCTGTTGATTACAAGTATGCACCATTTGTTGTTTCATTCTCAGAGATGCAACTCGCTGGCTGCACTTCTGATCCATTAGCATGCTCTAAATCCACCCCTTCTTCTGGTGTAGACCCTGTTAATGGACCTCAATTTACCAAGCTATCCCCACAACAGATGGCAGCACTTGATTGGGCTAGAAAGAAGCTCATGTTTTACTCCTATTGTACTGACAAGAACAGATATAAAGTCATGCCACCAGAATGCCACTAA

*>AhXTH13*

ATGGGATTGGGAGGAGGGTTGGTCACGTTCTTTTTGTGTCTACTATTTTTTGCTGCGCCTTCTGCTTCATCAACAAACTTATTGCCTATCATACCCTTCGATGAAGGATACGCACCCTTGTTTGGGGATAACAACTTGGTCATTCACAGGGATGGAAAAACCGTCCATCTTTCACTCGATGAGAGAACAGGTTCTGGATTCGTGTCTCATGATCTTTACCTCCATGGATATTTCAGAGCTTCCATTAAGCTACCTGCTGACTACACCGCTGGGGTTGTGGTTGCCTTTTATATGTCAAATGGTGACATGTTCCAGAACAACCATGATGAAATAGACTTTGAGTTCTTGGGGAATATAAGAGGCAAAGACTGGAGGATTCAGACCAATGTTTATGGCAATGGAAGTACAAGCATTGGCAGGGAGGAAAGATATGGCCTCTGGTTCGATCCTGCTGAGGATTTCCATCAGTACAGTATTCTCTGGACTGATTCTCAGATCATATTTTATGTAGATAATGTTCCTATTAGGGAAATTAAGCGGACGGAATCTATGGGAGGTGACTTCCCTTCTAAACCTATGACTTTGTACGCGACAATATGGGACGCATCTGATTGGGCAACCAATGGAGGCAAATACAGAGTGAATTACAAGTATGCACCCTATGTCGCCGAGTTCTCTGATCTTGTCTTGCACGGTTGTGCGGTCGATCCCATTGAACATGAAGCCAAGTGTGACAATGCTCAAACTTCCAAAGCAGTTCCTACTGGTGTTACACCAGCACAAAGAATCAAGATGGAGAACTTCAGGAAGAAGCACATGACATACTCTTACTGTTATGACAAAGTCAGATACAAAGTCCCTCCAACAGAGTGTGTCATTAATCCCCAAGAAGCTGAGAGGCTGAGAAAATTCGACCCTGTGACATTCGGTGGTGGCCGGCGCCGCCACGGAAAGCGACATTATCGCAGCAGAGGGAGCCAGGCAGAAGAAGCTGCTGCATTTTGA

*>AhXTH14*

ATGGGTTCTACTTGTACTAATCATAATGGGTTCTATGTGGGTATTATGGTGATTGGGTTAGTGGTAGTCAGCACCATGGTGGGTTCATGTGAAGGTAACTTCAACCAAGAGTTTGATCTAACATGGGGTGGTAACCGTGCCAAGATATTTGGCGGTGGCCAGCTTCTATCTCTTTCCCTAGACAGAGTCTCTGGCTCTGGCTTTCAATCAAAGAGAGAGTATCTCTTTGGTAGAATTGATATGCAGCTCAAGCTTGTTGCTGGCAACTCTGCTGGCACTGTCACTGCTTACTACTTATCATCACAAGGACCAACACACGATGAAATTGATTTCGAGTTCTTGGGAAACTTAAGCGGTGACCCTTACACACTGCACACAAACATCTTCACCCAAGGCAAAGGTGACAGAGAGCAACAGTTCCACCTTTGGTTTGATCCCACCAAGAACTTCCACACTTATTCTGTCATTTGGAAGCCCCAACATATCATATTCTTGGTTGATAACATCCCAATAAGGGTTTTCAAGAACGCAGAATCTATGGGGGTTCCTTTCCCAAAGAAACAACCAATGAGAATCTATTCGAGTCTGTGGAACGCAGATGATTGGGCCACGAGAGGTGGGCTTGTGAAGACTGATTGGGCCAAAGCACCGTTCACTGCATACTACAGAAACTTTAGGGCCACGCAGCTCTCATCAGCCTCTCTGAGGCCCAACACGCGCTCTTCAGAGTGGGAAACAAGTGACATTGACGCAATTGGAAGGAGAAGGCTGAGGTGGGTTCAGAAGTATTTCATGATCTATAATTACTGCAATGATTTCAAGCGATTCCCACAGGGCCTTCCTGCTGAATGTAGATCAAGGTTCTGA

*>AhXTH15*

ATGGGTGGTTCTACTACGAAAGGGTTTAACCTGCTAGTTGTTGGGGTTGTGGTGGTAGTTACCACCATGGTGGGTACGTGCAGCGCTAACTTCTACGACGATTTTGATCTAACATGGGGAGAAAACCGTGCAAAAATATTCAATGGAGGGCAGCTTCTCTCTCTTTCGCTCGATAAAGTGTCTGGCTCTGGCTTCCGATCCAAGAAAGAGTACATGTTTGGCAGAATTGACATGCAACTCAAACTTGTTTCTGGCAATTCCGCTGGCACTGTTACTGCTTATTATTTGTCATCAGAAGGAGCAACACATGATGAAATAGATTTTGAGTTTCTAGGGAACGTGAGTGGTGAGCCATACATTCTCCATACGAATGTGTTCAGTCAAGGTAAGGGCAACAGAGAGCAACAGTTCTATCTTTGGTTTGATCCCACAACAAATTTCCACACTTATTCTATCATTTGGAACCCCCACCACATCACATTCTTGGTTGATAACATCCCAATAAGGGTATTCAAGAATGCTGAATCAGATGGTGTTCCATTCCCAAAGAACCAGCCAATGAGAATCTATTCGAGTCTTTGGAACGCAGATGATTGGGCCACAAGAGGAGGTTTGGTGAAAACCGATTGGTCCAAAGCACCCTTCACGGCCTATTACCGCAACTTCAAGGTCACTGGCTTCTCCGCTACTTCTGCCTTCTCTGATGTTGCAACATCAGAGATACAGAATAGCGGAAAAGACAGCGTGCTCGATGCCTATGGCAGAAGAAGACTCAGATGGGTTCAGAAGTATTTCATGGTCTATAATTACTGCAACGATCTTAAACGCTTCCCAGAAGGCATTCCTGCTGAGTGTAGTCACGGAAGGTTTTGA

*>AhXTH16*

ATGGATCATAATAATACTTTACGTCGTTTTGGGGAAATAATACCCAAAACTCATCGTAATCATAATTTTCTCTTATCACTCTTTCTCTTCTTCTTCTTCTTCTTCTTCTTCTTCTTCTCATCACACGCAGCTTTTGACCTAGCTACCATCCCCTTCAACGATGGCTATTCTCCTCTCTTCGGAGATAGCAACGTCGTTCGCTCCGATGACGGCAACGGCGTTAATCTCCTCCTCGATCGCTTCACCGGTTCTGGCTTCATATCATCGAGTATGTACAAATATGGATTCTTCAGCGCTAATATCAAGTTGCCATCAAATTATACAGCTGGAATTTGCGTCGCTTTTTATACGTCAAATGGTGATGTGTTCGAGAAGACACACGATGAGTTGGACTTTGAATTCCTTGGTAACTTAGCGGGAAAACCGTGGCGGTTTCAGACCAACTTGTACGGCAACGGAAGCACCCACCGTGGCCGTGAGGAGAGGTATCGCCTGTGGTTTGATCCTACAAAGGAATACCATAGATACAGCATCCTTTGGACTGCCAAGAATGTTGTATTTTACATTGATGAAGTTCCAATTAGAGAGGTGCTAAGAAGTGCTGAAATGGGAGCTGATTACCCATCTAAACCAATGTCATTATATGCAACAATTTGGGATGCATCAAATTGGGCCACTTCAGGTGGAAAGTACAAAGTGAACTATAAATATGCACCTTTTGTAGCCGAATTTAAAGACCTAGTCCTCAAGGGTTGCTCCGTTGACCCTATTGAAGAGTCCACCGTGGCCGGCCGAAGCATTTGCTCCGACCAACACGCCGATCTTGAGGCACAAGACTATGCTGCCGTGACCCCAAGGCGCCGCCTAGCGATGCGCCGGTTCCGGCAGCGTTACATGTACTATTCTTATTGTTATGATACATTAAGGTACCCTAATCCATTACCTGAATGTGACATTATACCATCGGAGAAACAAAGATTTAAGGAGACTGGAAGGTTGAAATTCGGCGGCAGCCACCGCCGGCAGTCTCGGCGGAAGGGCCGGACTACAACTCCGGTGGATGATACCGATCAAGGTGATATGTGA

*>AhXTH17*

ATGTACATGGCATTCTTTAAAAACCCTTTCTTTTTATTATTATCATTATGGGCTCTCGTATTATCTGGTGTGTGTGTTTGGGGAAAACCAGTTACATTCCTTCAAGACTTTAGAGTTACATGGTCTGATTCCCACATCAGGCAAATTGATAATCAAGGCACCGCCATACAACTCATTCTAGACCGAAATTCTGGATGCGGATTTGCTTCAAAGAGTAGGTACATGTTTGGGCGTATTAGCATGAAGATTAAGCTCATTGCCGGAGACTCTGCTGGGACTGTGACGGCATTTTATATGAGCTCGGACACGGACGCTATAAGGGACGAGCTTGACTTCGAGTTCTTGGGAAACCGTAGTGGTCAACCATACACTGTTCAGACTAACATCTATGCCCACGGCAAAGGGGGTAGAGAACAAAGGGTCAACCTCTGGTTTGATCCTTCCGCCCAATTCCACACTTACACTATTCTCTGGAACCACCACCACATTGTGTTCTACGTGGATGAGTTTCCGATAAGAGTGTACAAGAACGACGTGGCAAGGGGAGTGGCATACCCAAGAATGCAAGGAATGGGAGTGTACTCAACGCTGTGGGAAGCAGATAACTGGGCAACAAGGGGAGGCTTGGAGAAAATTGATTGGAGGAAAGCACCCTTCTATGCATACTACAAGGACTTCGACATTGAAGGCTGCCAACTCCCAGGGCCCACCAGTTGTGCCTCCAACGCAAGTAACTGGTGGGAGGGTGCTGCTTACCAGGCCCTTACTCCCACTCAAGCCCGCTTGTACAGGTGGGTTCGCATCAACCACATTATCTATGATTATTGCCAAGACAAGCCACGATTCCCACTCGGCCCACCACCCGAGTGCCTTTCCTAG

*>AhXTH18*

ATGGTGAGAATGGAAGCAAAAGCTTCATCATCATTGGGCGTGATCCTTCTTCTCGTAGTCATAGCTGAAGCAGCTGTGTCCAAAGGATCCTTTGAAGATAATTTCAGCATAATGTGGTCTGAAGACCATTTTAGTACCTCCAAGGATGGGCAGATCTGGTATCTCTCACTTGACAAAGACACAGGATGTGGATTTCAAACAAAGCAGCGATACAGATTCGGGTGGTTCAGTATGAAGCTGAAATTGGTGGCAGGTGACTCTGCTGGAGTTGTCACAGCTTACTATATGTGCTCGGAGAACGGGGCAGGGCCAGAAAGAGATGAGCTGGACTTTGAGTTCTTGGGGAACAGAACAGGGCAGCCGTTCTTGATTCAGACAAATGTATACAAGAATGGAACTGGAGGCCGTGAGATGAGGCACATGCTTTGGTTCGATCCAACTGAGGACTACCACACCTATTCGATTCTCTGGAACAACCACCAAATTGTGTTTTTCGTGGATAGAGTGCCCATAAGGGTGTTCAAGAACAATGGGAAGGAAAACAATTTCTTCCCGAATGAGAAGCCCATGTACTTGTTCTCGAGCATATGGAACGCAGATGACTGGGCCACAAGAGGAGGACTGGAGAAGACGAACTGGAAACTAGCACCCTTTGTGTCATCATACAAGGACTTCAGCGTCGACGGCTGCCAATGGAAAGATCCATATCCTGCATGTGTATCAACCACCACCGACAATTGGTGGGATCAATACTCTGCCTGGCACCTCTCCGATGATCAGAAGAAGGATTATGCTTGGGTTCGGAGGAACCTCGTCATCTATGACTACTGCAACGATTCTGAACGCTATCCAACCCTTCCAGAAGAGTGTTCTTTGAGCCCCTGGGATTAA

*>AhXTH19*

ATGGCAGCAGCATCCGAAAAAATGTTTTTGGCTTTGTTGTTCATATTTTTCATGGCACGAGGGATTATCATAGTGGATGCAAACTTTGGTAAAAGCATGTATCTCACATGGGGTACCCAACATGCTTCAATTCAGGGTGAAGATCTTCAGCTTGTGTTACATCAAACTTCAGGTTCTGCTGCTCAAACAAAGATACCATTCTTATTTGGAAGTATAGAATCGAAAATCAAACTGGTACCTAATAATTCTGCAGGAACTGTTACAGCCTATTATCTATCCTCTACAGGAAGTCAACATGATGAGATAGACTTTGAGTTCTTAGGCAACATTTCAGGACAACCATATATTGTCCACACAAACATATATACACAAGGAAATGGAAGCAAAGAGCAACAATTTTACCTTTGGTTTGACCCTACCGCTGATTTTCACAATTACACCATTCATTGGAACCCCACTCAAGTTGTGTGGTATATTGATAGCATACCGATTAGAGTTTTTATGAACAATGAAGAGGAGGGCATAGCATACCCAAACAAGCAAGGAATGAAAGTATACACAAGCCTATGGAATGCAGATGATTGGGCCACAAGGGGTGGTTTGGTGAAGACTAATTGGACCAATGCACCATTCATTGCAAGGTTGAACCGTTTCAGAGCAAGGGCTTGCAAATGGAATGGACCAATTAGCATAAACAATTGTGCCTCTAATGTCCCTTCTAATTGGTGGACTTCACCCATTTACAAGCAATTAAGCTATGCACAGATGGGTCAGTTATATTGGGTTAGAAACAATTACATGATCTATGATTACTGCAAAGATACCAAGAGATTCAATGGTCAAAGTTCAGACTTTGTGATTTAA

*>AhXTH20*

ATGGCTCTTTTTGTATTTGCTATTTTAATGTTGATGGCCCCTTCAAGCAATGCTGAGTGGCCACCTTCACCTGGCTACTGGCCAAGTTCCAAATTCAAGACGATGAACTTTTACAAAGGTTTTAGGAACCTTTGGGGCCCCCAGCACCAAAGAATAGAACAAAATGCATTAACAATTTGGCTTGATAGAACCTCAGGAAGTGGCTTCAAGTCTGTTGCTCCGTTTCGATCCGGATACTTTGGTGCTTCCATTAAGCTCCAACCTGGCTACACTGCTGGAGTTATAACAGCTTTCTATCTTTCCAACAATGAAGCTCATCCTGGATTCCATGATGAAGTAGACATTGAGTTTCTTGGGACTACGTTTGGAAAGCCTTACACTTTGCAGACCAATGTTTATATCAGAGGAAGTGGAGATGGTCAAATTATAGGCAGAGAGATGAAGTTCCATTTATGGTTTGATCCAACCAAAGATTTTCATCACTATGCTATTCTCTGGAGTCCTAAGGAAATAATATTCTTTGTGGATGATGTGCCAATAAGGAGGTACCCGAGGAAAAGTGCAGAGACATTTCCAATGAGGCCAATGTGGCTTTATGGTTCAATATGGGATGCATCATCATGGGCAACCGAGGATGGCAAGTACAAAGCTGATTATAGGTACCAACCCTTTGTGGCAAGGTACACAAACTTCAAGGCCAGTGGTTGCACGGCGTATGCGCCGCGCTGGTGCCACGCCGTCTCAGCCTCGCCCTATAGGTCCGGCGAGTTGAGTAGGCAGCAATATAGTGCCATGAGATGGGTGCAAATGTACCACATGGTTTACAACTATTGTCAAGACTCCAAAAGGGATCATAGGCTAACACCTGAGTGTTGGAGTTAA

*>AhXTH21*

ATGGGTTCAAGGCGTGTAGGTGTATTGACCCTAAGTCTTGTTATTGTAGCGTCACTGGTGTCCGCTGCAATGTGCGGCGTTCCCAGAAGACCCGTTGATGTTCAATTTGGCCGTAACTATGTTCCCACTTGGGCCTTTGACCACATCAAATACTTCAATGGCGGTTCTGAGATTCAGCTCCATCTTGATAAATATACTGGCACTGGATTCCAGTCCAAAGGTTCATACTTGTTTGGTCACTTCAGCATGTACATCAAGATGGTTCCTGGTGATTCCGCTGGCACTGTAACCGCTTTCTATTTGTCTTCACAAACTGCGGAGCATGATGAAATAGACTTTGAGTTCTTGGGGAACAGAACAGGACAACCTTACATATTGCAAACCAATGTGTTCACCGGAGGTAAAGAGAACAGAGAATCTATCTCTGGTTTGATCCCACCAAAGAATACCACAGATATTCAGTTCTTTGTGGATGATGTACCAATTAGGGTGTTCAAGAACTGCAAGGACTTGGGTGTGAAATTCCCATTTGACCAACCAATGAAAATCTACAACAGCTTATGGAACGCAGATGATTGGGCCACAAGGGGTGGTTTGGAGAAAACAGATTGGTCGAAAGCACCATTCATAGCTTCCTACAAAGGCTTCCACATTGATGGTTGTGAAGCCTCCGTTGAAGCCAAGTTCTGTTCCACTCAAGGTAAGAGATGGTGGGACCAACAAGAATTCAGAGACCTTGATGCTCTTCAATGGAGGAGGCTTAGATGGGTTCGCCAGAAGTTCACTATTTACAACTATTGCAATGATAGAAAGCGTTACCCTACTCTTCCTCCTGAATGCTCTAGAGACCGTGACATTTAA

*>AhXTH22*

ATGGGTTATTATTCTATGTGGAGTGTTTGTTGTGTGATATTGGGATGGTTTGTTTCAGTTGGAATATGCGGCACACCAAGGAGACCCATGGCGGTTCCATTTGGAAGAAACTATGTTCCAACTTGGGCTTATGATCACATCAAATATTTGAATTCTGGTTATGATGCTCAGCTTCTTCTTGACAAGTACACTGGTACTGGCTTCCAATCCAAAGGTTCATACTTGTTTGGTCACTTCAGCATGGATATAAAGATGGTTGCTGGAGATTCCGCTGGAACAGTCACTGCTTTCTACTTATCTTCCCTAGGGACAGAGCATGATGAGATAGACTTTGAGTTCTTAGGGAACAGAACAGGACAACCTTACATTTTGCAAACAAATGTATTCACCGGTGGCAAAGGTGATAGAGAACAAAGAATCTATCTCTGGTTTGATCCTACCAAAGAATACCACAGATATTCAGTTCTATGGAACTTGTATCAAATTGTATTCTTTGTGGACAACATCCCAATTAGGGTGTTCAAGAACAACAAGCGAATGGGTGTGAAATTCCCATTTAACCAACCAATGAAGATCTACAACAGCCTATGGAACGCAGATGATTGGGCCACAAGGGGTGGTTTGGAGAAAACAGATTGGTCCAAAGCACCATTCATAGCTTCCTACAAGGGCTTCCACATTGATGGCTGTGAAACCTCCGTTGAAGCCAAGTTCTGTTCCACTCAAGGTAAGAGATGGTGGGACCAACAAGAATTCAGAGACCTTGATTCTTATCAATGGAGGAGGCTTAGATGGGTCAGAAGGAGATTCACCATTTACAACTATTGCAGTGATAGAACAAGGTACCCTCAGATGCCACCTGAATGCAGAAGAAACGGTGACTATTGA

*>AhXTH23*

ATGCCATCATTGCTTTCCTCTTCTCTCCTTCAAATGCCTCCTCTTCTATTCTCTCTTATACTCTCACTCATGCTTTGTGGAATCATTGCTGATGAGTCTCCACCTTCACCTGGCTACTACCCTAGTTCCCAAGTTAGTTCTGTTGCCTTTGATCAAGCTTATAGAAACCTTTGGGGACCTCAGCACCAAAGACTAGACCAATCTGGCTCACTAACTATTTGGCTTGACTCTTACTCTGGAAGTGGATTCAAGTCAATCCGGCCATATCGATCCGGATACTTTGGTGCTGCCATTAAGCTTCAATCTGGTTACACTGCAGGACTTTCAAACAACCAAGACTACCCAGGAGACCATGATGAAGTGGACATTGAGTTCCTTGGTACCATCCCAGATAAGCAATATGTGCTGCAGACAAATGTGTTCATGCGAGGAAGCGGAGACAAGAATAATGTGATAGGAAGAGAGATGAGGTTTCACCTTTGGTTCGATCCAACACAAGATTTTCACCACTATGCTATTCTATGGACACCCACTGACATCATATTTTTGGTGGACGATGTTCCAATAAGGAATTACCCAAGAAAGAATGATGCAACATTCCCTGAAAGAGCAATGTACGTGTACGGATCGATATGGGACGCATCATCATGGGCCACAGAGAATGGGAAATACAAAGCTGATTACAAATACCAACCATTCATTGGAAGATACAAAGATTTCAAGCTCCAAGGTTGCACCACTCAATCCTCCTCCTCATGCCAGCCACCCTCACCTTCACCACCTGGCTACAATTCACTAAGCCCTCAACAGTATAATGCAATGCAATGGGTCCAAAACAATTACTTGGTCTATGACTATTGCCGTGATCCCACTAGAGACCATACCCTTACTCCAGAATGCTAA

*>AhXTH24*

ATGTCTAAGATGTCTAGTCTCTTGGGATTCTTTGTGGGTCTAGTTTTGGTGGGGGTTGTTGCCTCTTCCAAGTTTGAAGAACTCTACCAACCTGCTTGGGCCTTGGATCATTTCATCCACGATGGCGAACTCATTAAACTCAAGCTTGATAACTATTCAGGTGCTGGTTTTGGATCAAAGAGCAAATATATGTTTGGGAAAGTGAGCATCCAACTTAAGCTTGTGGAGGGTGACTCTGCTGGAACCGTTACTGCTTTCTATATGTCATCGGAGGGTCCAAATCACAACGAATTTGATTTTGAGTTCTTGGGGAACACTACTGGTGAGCCTTATTCGGTGCAGACGAATGTGTATGTGAATGGTGTTGGTAACAGGGAGCAGAGACTCGACCTATGGTTCGATCCCACCAAGGACTTCCACACCTACTCTATCTTCTGGAATCAACGCCAAGTTGTGTTCCTAGTGGACGATACGCCAATAAGGGTGCACACAAACCTTGAACACAAGGGAATCCCTTTCCCTAAAGACCAAGCAATGGGAGTGTACAGCTCAATATGGAACGCAGATGATTGGGCCACACAGGGTGGTAGGGTGAAGACAGATTGGAGCCATGCACCATTCGTTGCCACATACAAGGACTTCACGATTGACGCGTGTGAGTGCCCAGTGGGAGTGTCATCGTCATCAGTGGCCCCGGAAAATGCTAAGAGGTGCAGTAGCAGCGAGGATAAGAAGTATTGGTGGGATGAACCAACTATGTCGGAGTTGAACGTTCACCAGAGCCACCAGCTTATGTGGGTTAGGGCTAACCATATGGTCTATGACTATTGCACTGATACTGCTAGGTTCCCAGTCACACCCGCTGAGTGTGTCCACCACCGCCACTAA

*>AhXTH25*

ATGAATAACTTGCAGATAGCACTCTTCTTCCTCATTGGGATTGTGTCCTCCATTTTGTTTCATATTTCAGTTGCATCTGTTGTTTCAACAGGAAACTTCAATAAGGACTTCTATGTTTTATGGTCACCTACCCATGTAAACACATCTGCTGATGGACACACTAGAACTTTGAAGCTTGATCAACAATCCGGGGCTGGTTTTGCTTCAAATCAGATGTTTTTGTTTGGACAAATTGACATGCAAATCAAACTAGTACCAGGTGATTCTGCAGGCACAGTATTAGCCTACTATATGGCATCTGATCAACCAAATCGCGACGAGATTGACTTTGAGTTTCTAGGAAACATGTCTGAGCAGCCTTATATTCTTCAAACAAATATTTATGCAGATGGGTTTGGCAATAGAGAGGAGAGGATTTATCTATGGTTTGATCCTACAAAGGACTTCCATACTTACTCAGTGTTGTGGAATCTGCACCAGATTGTGTTCATGGTGGATAGCATTCCAATAAGAGTGTACAGAAACCATGGTGACAAGGGAGTTCCATTTCCAAGAAGGCAACCAATGAGTCTAGAAGCAACTCTTTGGAATGGTGATAGCTGGGCAACAAGAGGAGGTCAAGACAAGATAGATTGGACAAAGGGTCCCTTCATAGCTTCATTCAGGAACTACAACATTGATGCTTGTGTGTGGAAAGGGAACCCAAGGTTCTGCAGAGTAGCTAGCCATGTTAATTGGTGGAACCTAAACAACTTCAGCACACTCACATCCCCACAAAGAAGGTGGTTCAAATGGGTCAGGAAATACCATATGATTTATGATTATTGCCAAGACAATGAGAGGTTCCAAAACAATCTTCCACAGGAATGGTCCCTTCCCAAGTATTGA

*>AhXTH26*

ATGGGAGGGTGTCATCTGTGTTTTCTCTTCTTGTGTTTGTCCTCAGCCATGGTTGTTGTTTCTGGTTCTTCTTCCATGAACAATAATTTGCCTATCATAGCCTTTGAGGATGGTTACACACCCTTGTTTGGAGATAATAATTTGATTATCCATAATGATGGCAAATTGGTTCATCTTACACTTGATCAGGGAACAGGTTCTGGATTTGTGTCTCATGAACTTTACCTTCATGGGTATTTTAGTGCTAAGATTAAGTTGCCTGCTGATTACACTGCTGGAGTTGTGGTGGCTTTTTATATGTCAAATGGTGACATGTTCAAGAAGAACCATGACGAAATAGACTTTGAGTTTTTGGGAAATATTAGAGGCAGAGATTGGAGAATGCAGACCAATTTTTATGGTAATGGTAGTACCAACACTGGCAGAGAAGAAAGATATGATCTCTGGTTTGATCCTTCTCAAGATTTTCACCAGTATAGTATTCTGTGGACAGATTCTAAGATCATATTTTATATAGATAATGTTCCAATAAGAGAAGTGAAGAGAACAGAATCCATGGGTGGAGATTTTCCTTCAAAGCCAATGACTTTGTATGCAACCATATGGGATGCATCTGATTGGGCAACTGATGGAGGAAAATACAGAGTAAATTACAAATATGCCCCTTATGTTGCTGAGTTCTCAAACTTTGTCATGCATGGTTGTGGGGTTGATCCAATTGATGAAAATGTTGCAATGTGCAACAATGCTCAAAATTCTAAGGCAAATATCATACCTAAACACAAAATCAAGATGGAGAACTTTAGGAACAATCATATGACATACTCTTATTGTTATGATAGGGCTAGGTACCAAGTTCCTCCACCTGAGTGTGTCATTAGTCTCCAAGAGGCTGAAGCACTAAGAAAACTTGATCCTGCCACCTTCGGTGGCGGTCGGCGCCACCGGGGTGGTGGAAAGCGGCGGCACCATCAAAGCAAAGGAAGGAAGGCAGAGGATGCTTCATTTTGA

*>AhXTH27*

ATGGCTCCTTCATGGGGTGGTTCATATTTTCTCCTTCTTTGTATGTTGTTAGGGTTCAGCACAATTGCATATGGTGGGAATTTCAACACTGATTTTGACCTTCTATTTGGAGATGATAGGGTTAAGATTTCTGATGGTGGACAAAGCATGTCTCTCTCAATGGATAAATATTCTGGTTCTGGGGTTGCTACCAAGGATCAATTCTTGTTTGGAAGATTTGACATGAAGATCAAGCTTGTTGGAGGAAACTCTGCAGGAACTGTTACTGCATTTTATCTAAGCTCTCAAGGAGATCACCATGATGAGGTTGATCTTGAGTTCTTGGGGAACTTAACCGGAGATCCATATATGCTATCAACAAATGTGTTTGCAAATGGTGTTGGGGGTCGTGAGGTTCAATACTATCTTTGGTTTGATCCAACAGCAGATTATCACACCTATTCCATTGATTGGAGCCCTACCCGCATAATAATCTGGGTGGACGACACTCCAATCAGAGTGATAAACAACAAAGAAATAATCGGTGTTCCATTCCCAACAAAGCAACCAATGAGACTGTACACAACACTCTGGAATGGGGATGCATGGGCAACAAGGTGGGGCCAAGTGAAGATTGACTTGTCACAAGCACCATTTATAGCAAGGTTCAAGAACTACAATGGCACTGCTTGTGTTCCAAAGAAAGGTATAGCAGATTGCAAGGGTTTTGGTGCTTCAATGAAGAAAGGTATTGACAATGAAAACAAGAAAAAGTTGAAGCAAGTTAACTCAAAGTGGGTTGTTTATCACTATTGCCGTGATTTGAGGCGTTATGCTCATGGATTACCCTTTGAATGCCGTAGGGACAACATGGCACACAGTGATAATAATCAATAA

*>AhXTH28*

ATGGCCACCTTTTATCCTCTCATTAGAAATGGTGGTGGTTCATTATTTCTAATACTGCTATCATGTGTTTTCCTAGTATCTTCTTTATTATGTGTTTTGGGACGACCAGCCACTTTTGAGCAAGACTTTAGAGCCACGTGGTCTGAGTCTCATATCAGGCATATTGATCAAGGCAGAACAATCCAACTCATGCTCGACCAAAGCTCCGGATGTGGGTTTGCTTCCAAGGTGAAGTACATGTTTGGGCGTGTTAGCATGAAGATCAAACTCGTCCCTGGAGACTCTGCTGGCACCGTCACCGCATTTTAATGAATTCTGATACAGATTCTGTTCGTGATGAGCTGGACTTCGAGTTCTTGGGGAACAGGAGTGGACAGCCATACACGGTTCAAACAAACGTATATGCTCATGGAAAGGGTAATAGAGAGCAAAGGGTCAACCTCTGGTTTGATCCTTCTGCTGATTTCCATACCTACACTATTCTCTGGAACCATAAACATATTGTATTTTCCGTGGACGATTTCCCCATCAGAGTATACAAGAACAACGAAGCGAAAGGGGTGCCATACCCAAAGATGCAAGCCATGGGAGTGTACTCAACACTATGGGAAGCAGATAACTGGGCAACAAGAGGTGGGTTAGAGAAAATCGATTGGAGCAAAGCACCCTTCAATGCTTACTATAAAGACTTCGACATTGAAGGGTGTTCAGTTCCGGGTCCGGCAAGCTGCTCCTCCAACCCGAGTAACTGGTGGGAAGGGGCAGCCTACCAAGAACTTAGTGCTATTCAAGCAAGGAGGTACAGATGGGTTCGAATTAACCACGTTGTCTATGACTATTGTCAAGATAAGTCAAGGTTCCCAGTCACCCCACCTGAGTGTCTTGCTGGCATTTGA

*>AhXTH29*

ATGGCTGCTTCAACTCTCTCCTACTTGCTTCTAATCCCTCTTCTGATGGTTGTTGCATATGCTGGTAACTTAGACCAAGAATTTGACATTACATGGGGCGATGGGCGAGCCAAGATGCTCAACAATGGGGAGCTACTCACATTGTCACTTGACAAAGCCTCTGGTTCTGGCTTCCAATCCAAGAACGAGTATCTCTTTGGCAAGATTGACATGCAGCTCAAGCTTGTTCCTGGGAACTCTGCTGGCACTGTAACTGCCTATTATTTATCTTCAAAAGGAACAACTTGGGATGAGATAGACTATGAATTCCTTGGGAATTTGAGCGGAGATCCATACATTCTTCACACCAATGTTTTCAGCCAAGGCAAAGGAAACAGGGAACAACAATTCTATCTATGGTTTGATCCTACTGCTGATTTCCACACTTACTCCATCACTTGGAACCCCCAAAGGATTATATTCTCAGTGGATGGAACTCCAATCAGAGAGTTCAAGAACTCAGAGAGCATGGGTGTCCCCTTCCCAAAGAGCCAGCCAATGAGAATATATTCAAGTTTGTGGAATGCAGATGACTGGGCAACAAGAGGTGGACTTGTGAAGACAGATTGGACAAATGCTCCATTCACAGCTTCATACAGGAACTTCAATGCTGATGCTTGCATATGGTCTAATGGAGCTTCATCTTGTGGTTCTGGTTCAGGTTCTTCCTCTACTAGCAGTTCATGGCTTTCACAAGAGTTGGACACTACAGCACAAGAGAGGCTAAGATGGGTGCAGAAGAACTATATGATATACAATTACTGCACAGATGCAAAGAGATTTCCACAGGGTTTTCCTCCAGAATGCAGAACCTCTTAA

*>AhXTH30*

ATGGCTTCATCAAAAGTAGTAGTGCTTGTTGTTCCCTTACTTGTGATGAGCTTTTGCATGGTGTCATGTTGGGGTGGTAACTTCAATAAAGACTTTCAGATAACGTGGGGCGATGGTCGTGCTAAGATACTCAACAACGGGAATCTGTTGACCCTGTCCCTTGACAAAGCCTCTGGCTCCGGTTTCCAGTCCACCAACGAGTATCTGTTTGGCAAGATCGACATGCAACTCAAACTTGTCCCTGGAAACTCCGCTGGCACCGTCACTGCCTATTATCTATCATCAAAAGGAGCAACATGGGATGAGATTGACTTCGAGTTCTTGGGGAATTTGAGTGGCGACCCTTACATCCTTCACACCAATGTCTTCAGCCAAGGCAAGGGTAACAGGGAACAACAATTCTACCTTTGGTTTGACCCAACCGCAGATTTTCACACTTATTCTATTCTCTGGAATCCTCAACGCATTGTATTCTCTGTGGATGGAACCCCTATAAGGGAATTCAAGAACTTGGAATCAGCTGGTGTTCCATTTCCAAAGAATCAACCAATGAGAATCTATTCAAGTCTGTGGAACGCAGATGATTGGGCTACAAGAGGTGGACTTGTTAAGACTGATTGGTCCAAAGCTCCATTCACTGCTTCCTACAGAAACTTCAATGCCAACAATGCATGCATATGGAAGAATGGAAGATCATCATGCAAGAGTTCCTCGTCATGGCTGTCACAGGAGCTTGATTCAACGGGTTTGCAGAGGCTGAGATGGGTGCAGAAGAACTACATGATTTATAATTACTGTACGGACAAGAAGAGGTTCCCTCGAGGATTTCCTATCGAATGCAACCGCTCTTAG

*>AhXTH31*

ATGAATCCTACTTCTCTGCCAAAGAAACAATTCCCTGACTCTCAACTGTTAGACCAAGAATTTGACATCACATGGGGTGATGGGCGGGCCAAGATGCTCAACAATGGAGAGCTACTCACATTGTCACTTGACAAAGCCTCTTGTTTCGGTTTCCAATCTAAGAACGAATTTCTCTTTGACAAGATTGACATGCAGCTCAAGCTTGTTTCCGGAAACTCAGCAGGCACTGTCACTGCCTACTATTTATCATCAAAGGGATCGAATTGGGATGAAATTGACTTTGAATTCTTGGGAAATGTGAGTGGAGAGCCTTACATTCTGCACACCAATGTGTTCAGCAATGGCAAAGGCAATAGAGAGCAACAGTTCTATCTCAGGTTCGACCCCACTACTGATTTCCACACCTACTCCATTCTTTGGAATCCTCAGCTCATTATGTTTTCCGTGGATAGTAGTCCAATAAGGCAATAA

*>AhXTH32*

ATGCTTCTGCCTTATCCCACTAGTGTTGTTATGGTGGCACTATTCAGTATGACGATGATGATGATGTTGTGTGGTGGTGATTTGCATAAGAAAATTGACATAACATGGGGTAACGGGCGTGCCCAGATGCTCAACAATGGCCAGCTTCTTACTGTGTCCCTTGACGCTGCCTCTGGCTCTGGATTCCAATCCAAGGATCACTATCTTTTTGGCCACTTCCAAATTCAACTCAAACTTGTTCCTGGAAATTCTGCAGGCACTGTCACTTCTTTCTATTTACAATCAGAGGGATCAACTTGGGATGAAATAGACTTTGAATTCCTTGGAAATCTGAGTGGCAACCCATATATTCTTCACACTAATGTAATCACACAAGGTAAAGGTGGCAGGGAGCAACAATTCTATCTATGGTTTGATCCCACTTCTGATTTTCACACTTACTCAATTCTGTGGAATCCACTATGCGTCATCTTATACGTAGATGGCATTCCTATTAGAGAATTCAAGAACTACGAATCAAGGAATATATCATTTCCAATGAAGAAACCAATGAGAATGTATGGAAGCTTATGGGATGCTGAAGATTGGGCTACAAAGGGTGGTGTAATCAAGACAAATTGGAGTGCAGCTCCATTCATTGCATATTTTAAAAATTTTTTTGTCAACGCTTGTGTTGAGTCTACTCCTCCTTCTATTAAGTCTTCTTGCACACACCTAAACAAAAAGAATAACAATCATAATTCCGTTGGTGAAAAATGGATCACACAAGAGTTGAAGCCCTCTGAAGTGGACAAACTCAACTGGGTGCACAAGAATTTCATGGTCTATAACTATTGTTCAGACTTAAAACGCTTCCCTCAAGGATTGCCACTAGAATGTAAAATAAACTAA

*>AhXTH33*

ATGGTTGCTTCTTGTGTGAACAATAATAATGATGCATTGATTGTAATTGTCATAACATTGTTCCTAATATTGCCTCCTTCTTCTATGGCTGGTAGCAATTTCAACCAACAAGTTGATATCACTTGGGGCGACGGTAGAGGTAAGATTCTCAATAGTGGCAAGATTCTTACCCTGTCTCTAGACAGGGCTTCTGGCTCTGGCTTCCAGTCCAAGAATCAGTATCTGTATGGCAAGATTGACATGCAAATCAAGCTTGTCCCTGGAAATTCTGCTGGCACTGTCACTGCTTATTACTTACGTTCAGATGGAATATCATGGGATGAAATAGACTTTGAGTTCTTGGGAAATCTTAGTGGTGATCCTTACGTAGTTCATACAAATGTGTATACACAAGGAACAGGTGGCAGAGAGCAACAATTTTATCTCTGGTTTGATCCAACCGCAAATCTTCACACCTATTCCATTCTCTGGAATCCTGCACACATTGTGTTTTATATTGATGGGAGAGCAATAAGGGAGTTCAAGAACTTAGAGGGTGTTGGTATTCCCTACCCAAAGAACCAACCAATGAAACTGTATTCAAGCCTGTGGAACGCTGATGATTGGGCAACAAGAGGAGGGCTTGTGAAGACAGATTGGAGCCAAGCTCCATTCACAGCTTCCTTCAGGAACTTGAAAGCCAATGGTTGTGTTTGGTCCAATGGTGTTTCTTCATGCAACTTATCATCAAACTCCTCTGATAATAACAACTCTTGGCTCTCTCAACAGCTTGATTCAAACGGTCAGAGGAAGCTCAAATGGGTGCAGAAGAATTACATGATTTATAATTACTGCTCAGACATTAACCGATTCCCTCAGGGCCTCCCTCTTGAATGCACGCTCCTAACCACCTGA

*>AhXTH34*

ATGGGGATTGAGGTACTTATTATTAATAGTGTTCTCCTTATAGGGTTTGCGGTTGCTGCATCCGCAGGTAACTTCAACCAAGACTTTGAAATTACATGGGGTAATGACCGTGCCAAAGTTCTCAACAACGGACAGCTTCTGACGCTGTCCCTTGACAAGGCCTCCGGCTCCGGCTTCCGTTCCCGGAACGAGTACCTGTTTGGAAAGATCGACATGCAGCTTAAACTTGTTCCTGGTAATTCAGCAGGCACAGTCACAGCCTATTATCTAAGTTCACTTGGTGACACTCACGATGAGATAGACTTTGAATTTCTGGGGAACTTGAGTGGTGAACCATACACTCTGCACACGAATGTATTCACGCAAGGGAAAGGCAACAGAGAACAACAGTTCCATCTATGGTTCGACCCCACCAAGGACTTCCATACTTATTCTCTTCTTTGGAATCCTCAAAGCATCATATTCTCTGTGGACGGCACACCAATAAGGGAGTTCAAGAATTGGGAATCAAAAGGAGTTCCATTTCCTAAGAATCAAGCAATGAGGATATACTCAAGTCTATGGGACGCAGAAGATTGGGCCACAAGGGGTGGGCTTGTGAAGACTGATTGGACCCAAGCCCCATTCACAGCCTCATACAAAGGCTTCAATGCACAAGCTTGTGTTTGGAGCTCTTCTTCAGGCTCCTCTTGTTCTTCAAAACAAGGCCAATCATGGTTCACTCAGTCACTTGACTCAACTGGGCAAGCAAGAATCCAGTGGGTTCAGAAGAACTACATGATCTACAATTACTGCACTGATACCAAACGATTCCCACAAGGCCTTCCTCCTGAATGCACCCTTGCATGA

*>AhXTH35*

ATGGCTTCTTCAAGTCCCTCCTTGCTTCTAATCCCTCTTCTTGTTGGTTCTATAATGGTTGTTGCATATGGTGCCAAGTTAGACCAAGAAGTTGACATCACATGGGGCGATGGGCGCGCCAAGATGCTCAACAATGGGGAGCTACTCACTTTGTCACTTGACAAAGCCTCAGGTTCTGGTTTCCAATCCAAGAATGAGTATCTCTTTGGCAAGATTGACATGCAGCTCAAGCTTGTTTCTGGAAACTCTGCTGGCACTGTAACTGCCTATTATTTATCTTCAAAAGGAGCAACTTGGGATGAGATAGACTATGAATTCCTTGGGAATTTGAGCGGCGACCCCTACATTCTTCACACAAATGTTTTCAGTCAGGGCAAAGGAAACAGGGAACAACAATTCTATTTATGGTTTGATCCTACGGCTGATTTTCACACTTACTCCATCACTTGGAACCCCCAAAGGATTATATTCTCAGTGGATGGAACTCCAATAAGAGAGTTCAAGAACTCAGAGAGCATTGGTGTCCCCTTCCCAAAGAGCCAGCCAATGAGAATATATTCAAGTTTGTGGAATGCAGATGACTGGGCAACAAGAGGTGGACTTGTGAAGACAGATTGGACAAAAGCTCCATTCACAGCTTCATACAGGAACTTCAATGCCGATGCTTGCATATGGTCTAATGGCGCATCATCTTGTGGTTCTGGTTCTGGTTTAGGTTCTTCTTCTACTACTAGCAGTTCATCATGGCTCTCACAAGAGTTGGACACTACGGCACAAGAGAGGCTAAGATGGGTCCAGAAGAACTATATGATATACAATTACTGCACTGATGCAAAGAGGTTTCCACAGGGTTTTCCTCCAGAATGCAGAACCTCCTAA

*>AhXTH36*

ATGGCGGATCCAGTTCTTCACCCAGACACTACAAACCCTCTTCATCACCATCAAACGCAACCTCTCAAAGAGATCGCCATTGACTACACACCAGAAGCATGCTCTCATTGCCCTAATTCCAACACCATCACCCTCACCTTCGATCACCGCGGCGGCGCCAGGTGGCGCACCACCACTCGCTTCCACTATGGCACCTTCAGCTCCCTCATCCAGTGCCCTAAGGGTAACACCAATGGCCTCAACTTCAACCTCTACCTCTCTTCCTTAGAAGGTGAAAAGTCCCAAGATGAGATAGATTTTGAGTTCTTGGGTAAGGATAGGACCATTGTGCAGACAAACTACTTCAGTGGTGGCAATGGCAACAAGGAGAAGATTCACCATCTGGGTTTTGATGCCTCTGATGGTTTTCATGAGTATGTGATCAAGTGGAGTTGTGACGTGATAGAGTGGCTCATTGATGGGAAGGTAGTCAGAAGAGAGGAGAAGAAAGAAGGTAAAGGGTTCCCTCAGAAGCCTATGTTCTTGTATGCTTCCATTTGGGATGCAAGCTGCATTGCTAATGGGATGTGGGCAGGGAAGTATGATGGGAGTGATGCACCTTATGTTTGTCTCTATAAGGACATTCATGTCCCTACTAGCACTGCAGTTAAATGA

*>AhXTH37*

ATGATGAAGGCTCTACTATTATTTCTCTTTGCTATTTCTTCATCACTGTTTATGGAACCTTGTTATTCAAGCAGTGGATATTGGCCTCCTTCACCAGGATACTGGCCAAGTCACAAATTCAGGTCTATGAACTTTTACAAAGGATTTAGAAACCTTTGGGGTCCTCAACACCAAGCACTTGACAACAATAATGCATTAACAATTTGGCTTGATAGAACCTCAGGGAGTGGATTCAAATCAGTTCGTCCATTTAGATCAGGTTACTTTGGTGCTTCAATTAAGCTCCACCCTGGCTACACTGCAGGAGTTATAACAGCTTTCTATCTTTCTAACAATGAAGCACACCCTGGGTTCCATGATGAAGTGGACATAGAGTTTCTTGGGACCACATTTGGAAAACCTTATACTTTACAAACAAATGTTTACATAAGAGGAAGTGGGGATGGAACGATTATAGGAAGAGAGATGAAGTTCCATTTGTGGTTTGATCCTACCAAAGATTTTCATCACTATGCTATTCTTTGGTCTCCTAAAGAAATCATACCAATGTGGGTTTATGGTTCAATATGGGATGCATCATCATGGGCAACTGAAGATGGCAAGTACAAAGCTGATTATAGGTACCAACCTTTTGTTGCAAGGTACACAAATTTCAAGGCTAGTGGTTGCTCAGCCTATGCATCACGGTGGTGCCACCCAGTCTCAGCCTCACCATATAGGTCCGGTGGCTTGACCAGGCAACAATATTGGGCCATGAGGTGGGTCCAAAGACACCATATGGTTTATAACTATTGCCAAGACCCCAAAAGGGACCATAGATTAACACCTGAATGTTGGGGTAAAAAAAAGGAAAATAATGAAAAGAGAATGAGAAGCAGAGGGTATGTAATTGTGCATATATATGCTAGTTACTTCATTATATTATTGTTGACTGTTAGTTCTGAAACTTTTCAATTGGTTTCTTTTATTTCATTTACTCCAAGGAACCTTCAAGTGCAAAATTAA

*>AhXTH38*

ATGGGTTCTACTTGTACTAATCATAATGGGTTCTATGTGGGTATTATGGTGATTGGGTTAGTGGTGGTCAGCACCATGGTGGGTTCATGTGAAGGTAACTTCAACCAAGAGTTTGATCTAACATGGGGTGGTAACCGTGCCAAGATATTTGGCGGTGGCCAGCTTCTATCTCTTTCCCTAGACAGAGTCTCTGGCTCTGGCTTTCAATCAAAGAGAGAATACCTCTTTGGTAGAATTGATATGCAGCTCAAGCTTGTTGCTGGCAACTCTGCTGGCACTGTCACTGCTTACTACTTATCATCACAAGGTCCAACACACGATGAAATTGATTTCGAGTTCTTGGGAAACTTAAGCGGTGACCCTTACACACTGCACACAAACATCTTCACCCAAGGCAAAGGTGACAGAGAGCAACAGTTCCACCTTTGGTTTGATCCCACCAAGAACTTCCACACTTACTCTGTCATTTGGAAGCCCCAACATATCATATTCTTGGTTGATAACATCCCAATAAGGGTTTTCAAGAACGCAGAATCTATGGGTGTTCCTTTCCCAAAGAAACAACCAATGAGAATCTATTCGAGTCTGTGGAACGCAGATGATTGGGCCACGAGAGGTGGGCTTGTGAAGACTGATTGGGCCAAAGCACCGTTCACTGCATACTACAGAAACTTTAGGGCCACGCAGCTCTCATCAGCCTCTCTGAGGCCCAACACGCGCTCCTCTGAGTGGGAAACAAGTGACATTGACGCAGTTGGAAGGAGAAGGCTGAGGTGGGTTCAGAAGTATTTCATGATCTATAACTACTGCAATGATTTCAAGCGATTCCCACAGGGCCTTCCTGCTGAATGTAGATCAAGGTTCTGA

*>AhXTH39*

ATGTCTAAGGAGGTGTCGTTGTTTTTGGGATTGGTTATGGGGTTTGTGTTTGTTGGAGTGGCTTTTGCTGCTGCCACTGCCAAGTTTGAAGAACTCTTCCAACCAAGTTGGGCTTTAGACCATTTCATTCATGAAGGAGACCTTCTCAAACTCAAGCTTGATAACTATTCCGGTGCTGGGTTTGTATCCAAAAGCAAGTATATGTTTGGGAAAGTTACCGTCCAACTTAAACTTGTTGAAGGTGATTCTGCCGGAACAGTTACTGCTTTCTATATGTCATCGGAGGGTCCAAATCACAACGAGTTTGATTTTGAGTTCCTGGGGAACACCACAGGGGAACCTTACTCTGTTCAAACAAATGTGTATGTGAATGGAGTGGGAAATAGAGAGCAAAGGCTAAACCTTTGGTTTGACCCTACTAAAGACTTTCATTCTTACTCTTTCTTTTGGAACCAACGTCAAGTTATATTTCTAGTGGATGAAACACCAATAAGGGTGCACACAAACATGGAACACAGGGGTATTCCATTTCCAAAGGACCAAGCAATGGGTGTTTATAGCTCAATTTGGAACGCTGATGATTGGGCCACACAAGGTGGAAGAGTGAAAACTAATTGGAGCCATGCACCTTTCATTGCAACATATAAGGCCTTTGAGATCAATGCTTGCGAGTGTCCAATAGTGTCATCAAAATCAGTGGAAAATTTGAAGAGGTGTAGTAGTAATGAGAAGAAGTATTGGTGGGATGAGCCTAATTTGGGTGTGTTAAGTTTGCATCAAAGTCACCAACTTATGTGGGTTAGGGCCAAACATATGGTTTATGATTATTGTGCTGATACTGCTAGGTTCCCTGTTATGCCTGCTGAGTGTGTTCATCATAGTCACCACAAACTAGTGCTCAAAAATTAG

*>AhXTH40*

ATGTTGGTGGCTTTGTTTATATGTGTAGTCGTACTCGTTGGCAACATCGTCCAAGTGGATGGCAACTTTTCGAAGAGCATGTATCTCACATGGGGTGTTCAACATGCATCGATTATGGGCGAAGACCTTCATCTAGTGTTGGACACAACCTCAGGATCTGCTGCTAAATCGAAGAGATCATTCTTATTTGGAAGCATTGAAATGCTAATCAAGCTCATACCAGGCAATGCCGCAGGAATAGTAACAGCCTACTATTTATCCTCCACGGGAAGTCAGCATGATGAGATAGATTTTGAGTTCTTAGGCAACATTACAGGACAACCATACACTGTCAATACCAACATATATACACAAGGAAAAGGAAACAAAGAGCAACAATTTTACCTCTGGTTTGACCCAGCTGCTGACTTTCACAACTACACCATTCACTGGAACCCCACGCAAATTGTGTGGTATGTTGATGGTCTGCCGATTCGGGTTTTTCAGAACTACGAAAATCACGGCGTTGCGTATCCAAACAAGCATGGAATGAGGGTATACAGCAGCCTATGGAATGCAGATGACTGGGCAACTAGAGGAGGGCTTGTTAAGACTGACTGGAGAGGTGCACCATTCATAGCCAGCTTTCATCATTTCAGAGCAAGGGCTTGCAAGTGGAATGGGGCAGTGAGCATCAATCACTGTGCCTCCAATGTCCCTGCAAATTGGTGGATTTCTCCCCTATACAAGCAGCTAAGTTACTCCGAAAAAGGCCAGTTGAACTGGGTCAGAAAGAATTACATGATCTACGACTACTGTGCCGATTCCAAGAGATTCAACGGCCAGTTGCCTCCAGAATGTTCTAAAACACAACTCTAA

*>AhXTH41*

ATGACAACACTAGTAACTAAGGTCCCTATTGGGTTTCTTTCTCTTCTCTTAATTATTACTATAGCTACCAAAGCTGCGGCTGGTAACTTCTATCAAGACTTTGAAGTAACATGGGGTGATAACCGTGCTAAGATCTTTAACGGTGGCCAGCTTCTTACACTGTCCTTGGACAGAGCCTCCGGCTCCGGATTTCGCTCCAAGAACGAGTATTTGTTTGCCAAACTTGACATGCAAATCAAACTCGTTCCTGGTAACTCCGCTGGCACCGTTACAACATACTATCTATCTTCATTGGGTGGGACACATGATGAAATAGACTTTGAATTTCTGGGGAACTTGAGTGGAGATCCGTACATTCTTCACACAAACGTATTCACGCAAGGGAGAGGGAACAGAGAACAGCAGTTCTATCTATGGTTCGACCCCACCGCCGACTTCCACACCTACTCAATTCTTTGGAATCCTCACAACATCATATTCTCTGTGGACGGCACACCAATAAGGGAGTTCAAGAATTTAGAATCAAGAGGGGTCCCATTCCCAAAGAACCAACCCATGAGACTATACTCAAGCCTTTGGAATGCTGATGACTGGGCCACAAGGGGTGGGCTTGTGAAGACAGATTGGGCCCATGCCCCATTCACTGCCTCATACAGAAACTTCAATGCACCACCAGCCCAATCATGGATGGGCCAGTCTCTTGATTCAACAGGCCTTGCAAGAATCCATTGGGTCCAAAAGAATTACATGATTTACAATTATTGCACTGATCTCAAACGCTTCCCACAAGGGCCTCCTCCTGAATGCTCACTTGCATGA

*>AhXTH42*

ATGGCACTAATTTGGCAACTCAACCTTCTCTTCTCACCTCTCATAATAATATGCTTATTATCAAACGTGGTTTCTTCACGCACTAGGCCCTTCACAGCTCCAACTGTGACACCCTTGACTAATTCCTTCCCTCGTGTCCCAATTGATCCTGCTTTTTCTAATGCTTTTGGGGCCTCCAATGTCAAGCTCCTTGCTAATGGGACCATGGCCACTCTGGCTCTCGACAAACTCTCAGGCTCCGGATTGGTGTCCAAAAGCAGTTACTACTATGGATTCTTCAGTGCTGCAATCAAATTACCAGCTGGTATATCATCTGGAGTTGTAGTTGCCTTCTATTTGTCTAATGCAGATAAATTCCCTCATAACCATGATGAGATTGATATCGAACTCCTTGGGCATGACAAGAGGAACGATTGGGTTATCCAAACAAATGTGTATGCCAATGGAAGCGTGAGCACTGGAAGAGAAGAAAAGTTCTACTTCTGGTTCGACCCAACAAAGCAGTACCATTACTATAGCATCTTGTGGAACAGTTATCATACAGTGTTCCTAGTGGACAACATTCCAGTGAGGGAATTCATACACGGGACTGTTTTCCCATCAAAACCGATGTCGGTGTATGCAACAATATGGGATGGTTCAGAATGGGCCACACATGGTGGAAAATACCCAGTTGATTATAAGTATGCACCATTTGTTGTTTCATTCTCAGAGATGCAACTCACTGGCTGCACTTCTGATCCATTAGCATGCTCTAAATCCACCCCTTCTTCTGGTGTAGACCCTGTTAATGGACCTCAATTTACCAAGCTATCCCCACAACAGCTGGCAGCACTTGATTGGGCTAGAAAGAAGCTCATGTTTTACTCCTATTGTACTGACAAGAACAGATATAAAGTCATGCCACCAGAATGCCACTAA

*>AhXTH43*

ATGGGATTGGGAGGAGGGTTGGTCACGTTCTTTTTGTGTCTACTATTTTTTGCTGCGCCTTCTGCTTCATCAACAAACTTATTGCCTATCATACCCTTCGATGAAGGATACGCACCCTTGTTTGGGGATAACAACTTGGTCATTCACAGGGATGGCAAAACCGTCCATCTTTCACTCGATGAGAGAACAGGTTCTGGATTCGTGTCTCATGATCTTTACCTCCATGGATATTTCAGTGCTTCCATTAAGCTACCTGCTGACTACACCGCTGGGGTTGTGGTTGCCTTTTATATGTCAAATGGTGACATGTTCCAGAACAACCATGATGAAATAGACTTTGAGTTCTTGGGGAATATAAGAGGCAAAGACTGGAGGATTCAGACCAATGTTTATGGCAATGGAAGTACAAGCATTGGCAGGGAGGAAAGATATGGCCTCTGGTTCGATCCTGCTGAGGATTTCCATCAGTACAGTATTCTCTGGACTGATTCTCAGATCATATTTTATGTAGATAATGTTCCTATTAGGGAAATTAAGCGGACGGAATCTATGGGAGGTGACTTCCCTTCCAAGCCTATGACTCTGTACGCGACAATATGGGACGCATCTGATTGGGCAACCAATGGAGGCAAATACAGAGTGAATTACAAGTATGCACCCTATGTCGCCGAGTTCTCTGATCTTGTCTTGCACGGTTGTGCGGTCGATCCCATTGAACATGAAGCCAAGTGTGACAATGCTCAAACTTCCAAAGCAGTTCCTACTGGTGTCACACCAGCACAAAGAATCAAGATGGAGAACTTCAGGAAGAAGCACATGACATACTCTTACTGTTACGACAAAGTCAGATACAAAGTCCCTCCAACAGAGTGTCTCATTAATCCCCAAGAAGCTGAGAGGCTGAGAAAATTCGACCCCGTGACATTCGGGGGTGGCCGGCGCCGCCACGGAAAGCGACATTATCGCAGCAGAGGGAGCCAGGCAGAAGAAGCTGCTGCATTTTGA

*>AhXTH44*

ATGGGTGGGTTTAACCTGCTAGTGGTTGGGGTTGTGGTGGTGGTTACCACCATGGTGGGTACGTGCAGCGCTAACTTCTACGACGATTTTGATCTAACATGGGGAGAAAACCGTGCAAAAATATTCAATGGAGGGCAGCTTCTGTCTCTTTCGCTGGATAAAGTGTCTGGCTCTGGCTTCCGATCCAAGAAAGAGTACATGTTTGGCAGAATTGACATGCAACTCAAACTTGTTTCTGGCAATTCCGCTGGCACTTTGTCGTCAGAAGGAGCAACACATGATGAAATAGATTTTGAGTTTCTAGGGAACGTGAGTGGTGAGCCATACATTCTCCATACGAATGTGTTCAGTCAAGGTAAGGGCAACAGAGAGCAACAGTTCTATCTTTGGTTTGATCCCACAACAAATTTCCACACTTATTCTATCATTTGGAAGCCCCACCACATCACATTCTTGGTTGATAACATCCCAATAAGAGTATTCAAGAATGCTGAATCAGTTGGTGTTCCATTCCCAAAGAACCAGCCAATGAGAATTTATTCGAGTCTGTGGAACGCAGATGATTGGGCCACAAGAGGAGGTTTGGTGAAAACCGATTGGTCCAAGGCACCCTTTACGGCCTATTACCGCAACTTCAAGGCCACCGCCTTCTCCGCTACTTCTGCCTTCTCCAATGTTGTAACATCAGAGATACAGGGTAGCGGAGAAGACAGCGTGCTCGATGCCTATGGCAGAAGAAGACTCAGATGGGTTCAGAAGTACTTCATGGTCTATAATTACTGCAACGATCCCAAACGCTTCCCGCAAGGCATTCCTGCTGAGTGTAGTCACGGAAGGTTTTGA

*>AhXTH45*

ATGGATCATAATAATACTTTACGTCGTTTTGGGGAAATAATACCCAAAACTCATCGTAATCATAATTTTCTCTTATCACTCTTTCTCTTCTTCTTCTTCTTCTTCTTCTTCTTCTTCTCATCACACGCAGCTTTTGACCTAGCTACCATCCCCTTCAACGATGGCTATTCTCCTCTCTTCGGAGATAGCAACGTCGTTCGCTCCGATGACGGCAACGGCGTTAATCTCCTCCTCGATCGCTTCACCGGTTCTGGCTTCATATCATCGAGTATGTACAAATATGGATTCTTCAGCGCTAATATCAAGTTGCCATCAAATTATACAGCTGGAATTTGCGTCGCTTTTTATACGTCAAATGGTGATGTGTTCGAGAAGACACACGATGAGTTGGACTTTGAATTCCTTGGTAACTTAGCGGGAAAACCGTGGCGGTTTCAGACCAACTTGTACGGCAACGGAAGCACCCACCGTGGCCGTGAGGAGAGGTATCGCCTGTGGTTTGATCCTACAAAGGAATACCATAGATACAGCATCCTTTGGACTGCCAAGAATGTTGTATTTTACATTGATGAAGTTCCAATTAGAGAGGTGCTAAGAAGTGCTGAAATGGGAGCTGATTACCCATCTAAACCAATGTCATTATATGCAACAATTTGGGATGCATCAAATTGGGCCACTTCAGGTGGAAAGTACAAAGTGAACTATAAATATGCACCTTTTGTAGCCGAATTTAAAGACCTAGTCCTCAAGGGTTGCTCCGTTGACCCTATTGAAGAGTCCACCGTGGCCGGCCGAAGCATTTGCTCCGACCAACACGCCGATCTTGAGGCACAAGACTATGCTGCCGTGACCCCAAGGCGCCGCCTAGCGATGCGCCGGTTCCGGCAGCGTTACATGTACTATTCTTATTGTTATGATACATTAAGGTACCCTAATCCATTACCTGAATGTGACATTATACCATCGGAGAAACAAAGATTTAAGGAGACTGGAAGGTTGAAATTCGGCGGCAGCCACCGCCGGCAGTCTCGGCGGAAGGGCCGGACTACAACTCCGGTGGATGATACCGATCAAGGTGATATGTGA

*>AhXTH46*

ATGTACATGGCATTCTTTAAAAACCCTTTCTTTTTATTATTATCATTATGGGCTCTCGTATTATCTGGTGTGTGTGTTTGGGGAAAACCAGTTACATTCCTTCAAGACTTTAGAGTTACATGGTCTGATTCCCACATCAGGCAAATTGATAATCAAGGCACCGCCATACAACTCATTCTAGACCGAAATTCTGGATGCGGATTTGCTTCAAAGAGTAGGTACATGTTTGGGCGTATTAGCATGAAGATTAAGCTCATTGCCGGAGACTCTGCTGGGACTGTGACGGCATTTTATATGAGCTCGGACACGGACGCTATAAGGGACGAGCTTGACTTCGAGTTCTTGGGAAACCGTAGTGGTCAACCATACACTGTTCAGACTAACATCTATGCCCACGGCAAAGGGGGTAGAGAACAAAGGGTCAACCTCTGGTTTGATCCTTCCGCCCAATTCCACACTTACACTATTCTCTGGAACCACCACCACATTGTGTTCTACGTGGATGAGTTTCCGATAAGAGTGTACAAGAACGACGTGGCAAGGGGAGTGGCATACCCAAGAATGCAAGGAATGGGAGTGTACTCAACGCTGTGGGAAGCAGATAACTGGGCAACAAGGGGAGGCTTGGAGAAAATTGATTGGAGGAAAGCACCCTTCTATGCATACTACAAGGACTTCGACATTGAAGGCTGCCAACTCCCAGGGCCCACCAGTTGTGCCTCCAACGCAAGTAACTGGTGGGAGGGTGCTGCTTACCAGGCCCTTACTCCCACTCAAGCCCGCTTGTACAGGTGGGTTCGCATCAACCACATTATCTATGATTATTGCCAAGACAAGCCACGATTCCCACTCGGCCCACCACCCGAGTGCCTTTCCTAG

*>AhXTH47*

ATGGTGAGAATGGAAGCAAAAGCTTCATCATCATTGGGCGTGATCCTTCTTCTCGTAGTCATAGCTGAAGCAGCTGTGTCCAAAGGATCCTTTGAAGATAATTTCAGCATAATGTGGTCTGAAGACCATTTTAGTACCTCCAAGGATGGGCAGATCTGGTATCTCTCACTTGACAAAGACACAGGATGTGGATTTCAAACAAAGCAGCGATACAGATTCGGGTGGTTCAGTATGAAGCTGAAATTGGTGGCAGGTGACTCTGCTGGAGTTGTCACAGCTTACTATATGTGCTCGGAGAACGGGGCAGGGCCAGAAAGAGATGAGCTGGACTTTGAGTTCTTGGGGAACAGAACAGGGCAGCCGTTCTTGATTCAGACAAATGTATACAAGAATGGAACTGGAGGCCGTGAGATGAGGCACATGCTTTGGTTCGATCCAACTGAGGACTACCACACCTATTCGATTCTCTGGAACAACCACCAAATTGTGTTTTTCGTGGATAGAGTGCCCATAAGGGTGTTCAAGAACAATGGGAAGGAAAACAATTTCTTCCCAAATGAGAAGCCCATGTACTTGTTCTCGAGCATATGGAACGCAGATGAGTGGGCCACAAGAGGAGGACTGGAGAAGACGAACTGGAAACTAGCACCCTTTGTGTCATCATACAAGGACTTCAGCGTCGACGGCTGCCAATGGAAAGATCCATATCCTGCATGTGTCTCAACCACCACCGACAATTGGTGGGATCAATACTCTGCCTGGCACCTCTCCGATGATCAGAAGAAGGATTATGCTTGGGTTCAGAGGAACCTCGTCATCTATGACTACTGCAACGATTCTCAACGCTATCCAATCCTTCCAGAAGAGTGTTCTTTGAGCCCCTGGGATTAA

*>AhXTH48*

ATGGCAGCAGCATCTGAAAAAATGGTTTTGGCTTTGTTGTTCATATTTTTCATGGCACGAGGGATTATCATAGTGGATGCAAACTTTGGCAAAAGCATGTATCTCACATGGGGTACCCAACATGCTTCAATTCAGGGTGAAGATCTTCAGCTTGTGTTAGATCAAACTTCAGGCTCTGCTGCTCAAACAAAGATACCATTCTTATTTGGAAGTATAGAATCGAAAATCAAACTGGTACCTAATAATTCTGCAGGAACTGTTACAGCCTATTATCTATCCTCAACAGGAAGTCAACATGATGAGATAGACTTTGAGTTCTTAGGCAACATTTCAGGACAACCATATATTGTCCACACAAACATATATACACAAGGAAATGGAAGCAAAGAGCAACAATTTTACCTTTGGTTTGACCCTACCGCTGATTTTCACAATTACACCATTCATTGGAACCCCACTCAAGTTGTGTGGTATATTGATAGCATACCGATTAGAGTTTTTATGAACTATGAAGAGGAGGGCATAGCATACCCAAACAAGCAAGGAATGAAAGTATACACAAGCCTATGGAATGCAGATGATTGGGCCACAAGGGGTGGTTTGGTGAAGACTAATTGGACCAATGCACCATTCATTGCAAGGTTGAACCGTTTCAGAGCAAGGGCTTGCAAATGGAATGGACCAATTAGCATAAACAATTGTGCCTCTAATGTCCCTTCTAATTGGTGGACTTCACCCATTTACAAGCAATTAAGCTATGCACAGATGGGTCAGTTAAATTGGGTTAGAAACAATTACATGATCTATGATTACTGCAAAGATACCAAGAGATTCAATGGTCAAGTGCCTCCTGAATGTTTCAAGACACAGTTCTAA

*>AhXTH49*

ATGATGATGAAGGCTCTACTATTATTTCTCTTTGCTATTTCTTCATCACTGTTTATGGAACCTTGTTATTCAAGCAGTGGATATTGGCCTCCTTCACCAGGATACTGGCCAAGTCACAAATTCAGGTCTATGAACTTTTACAAAGGATTTAGAAACCTTTGGGGTCCTCAACACCAAGCACTTGACAACAATAATGCATTAACAATTTGGCTTGATAGAACCTCAGGGAGTGGATTCAAATCAGTTCGTCCATTTAGATCAGGTTACTTTGGTGCTTCAATTAAGCTCCACCCTGGCTACACTGCAGGAGTTATAACAGCTTTCTATCTTTCTAACAATGAAGCACACCCTGGGTTCCATGATGAAGTGGACATAGAGTTTCTTGGGACCACATTTGGAAAACCTTATACTTTACAAACAAATGTTTACATAAGAGGAAGTGGGGATGGAACGATTATAGGAAGAGAGATGAAGTTCCATTTGTGGTTTGATCCTACCAAAGATTTTCATCACTATGCTATTCTTTGGTCTCCTAAAGAAATCATACCAATGTGGGTTTATGGTTCAATATGGGATGCATCATCATGGGCAACTGAAGATGGCAAGTACAAAGCTGATTATAGGTACCAACCTTTTGTTGCAAGGTACACAAATTTCAAGGCTAGTGGTTGCTCCGCCTATGCATCACGGTGGTGCCACCCAGTCTCAGCCTCACCATATAGGTCCGGTGGCTTGACCAGGCAACAATATTGGGCCATGAGGTGGGTCCAAAGACACCATATGGTTTATAACTATTGCCAAGACCCCAAAAGGGACCATAGATTAACACCTGAATGTTGGGGTTAA

*>AhXTH50*

ATGTTGATGGCCCCTTCAAGCAATGCTGAGTGGCCACCTTCACCTGGCTACTGGCCAAGTTCCAAATTCAAGACTATGAACTTTTACAAAGGAAGTGGCTTCAAGTCTGTTGCTCCGTTTCGATCCGGATACTTTGGTGCTTCCATTAAGCTCCAACCTGGCTACACTGCTGGAGTTATAACAGCTTTCTATCTTTCCAACAATGAAGCTCATCCTGGATTCCATGATGAAGTAGACATTGAGTTTCTTGGGACTACGTTTGGAAAGCCTTACACTTTGCAGACCAATGTTTATATCAGAGGAAGTGGAGATGGTCAAATTATAGGCAGAGAGATGAAGTTCCATCTATGGTTTGATCCAACCAAAGATTTTCATCACTATGCTATTCTCTGGACTCCTAAGGAAATAATATTCTTTGTGGATGATGTGCCAATAAGGAGGTACCCGAGGAAAAGTGCAGAGACATTTCCAATGAGGCCAATGTGGCTTTATGGTTCAATATGGGATGCATCATCATGGGCAACCGAGGATGGCAAGTACAAAGCTGATTATAGGTACCAACCCTTTGTGGCAAGGTACACAAACTTCAAGGCCAGTGGTTGCACGGCGTATGCACCGCGCTGGTGCCACGCCGTCTCAGCCTCGCCCTATAGGTCCGGCGGGTTGAGTAGGCAGCAATATAGTGCCATGAGATGGGTGCAAACGTACCACATGGTTTACAACTATTGTCAAGACTCCAAAAGGGATCATAGGCTAACACCTGAGTGTTGGAGTTAA

*>AhXTH51*

ATGGGTTCAAGGCGTGTAGGTGTATTGACCCTAAGTCTTGTTGTTGTAGCGTCACTGGTGTCCGCTGCAATGTGCGGCGTTCCCAGAAGGCCCGTTGATGTTCAATTCGGCCGTAACTATGTTCCCACTTGGGCCTTTGACCACATCAAATACTTCAATGGCGGTTCTGAGATTCAGCTCCATCTTGATAAATATACTGGCACTGGATTCCAGTCCAAAGGTTCATACTTGTTTGGTCACTTCAGCATGTACATCAAGATGGTTCCTGGTGATTCCGCTGGCACTGTAACCGCTTTCTATTTGTCTTCACAAACTGCGGAGCATGATGAAATAGACTTTGAGTTCTTGGGGAACAGAACAGGACAACCTTACATATTGCAAACCAATGTGTTCACCGGAGGTAAAGGTGATAGAGAACAGAGAATCTATCTCTGGTTCTTTGTGGATGATGTACCAATTAGGGTGTTCAAGAACTGCAAGGACTTGGGTGTGAAATTCCCATTTGACCAACCAATGAAAATCTACAACAGCTTATGGAACGCAGATGATTGGGCCACAAGGGGTGGTTTGGAGAAAACAGATTGGTCGAAAGCACCATTCATAGCTTCCTACAAAGGCTTCCACATTGATGGTTGTGAAGCCTCCGTTGAAGCCAAGTTCTGTTCCACTCAAGGTAAGAGATGGTGGGACCAACAAGAATTCAGAGACCTTGATGCTCTTCAATGGAGGAGGCTTAGATGGGTTCGCCAGAAGTTCACTATTTACAACTATTGCAATGATAGAAAGCGTTACCCTACTCTTCCTCCTGAATGCTCTAGAGACCGTGACATTTAA

*>AhXTH52*

ATGGGTTATTATTCTTTGTGGAGTGTTTGTTGTGTGATATTGGCATGGTTTGTTTCAGTTGGAATATGCAGCACACCAAGGAGACCCATGGCGGTTCCATTTGGAAGAAACTATGTTCCAACTTGGGCTTATGATCACATCAAATACTTGAATTCTGGTTATGATGCTCAGCTTCTTCTTGACAAGTACACTGGTACTGGGTTCCAATCCAAAGGTTCATACTTGTTTGGTCACTTCAGCATGGATATAAAGATGGTTGCTGGAGATTCTGCTGGAACAGTAACTGCTTTCTACTTATCTTCCCTAGGGACAGAGCATGATGAGATAGACTTTGAGTTCTTAGGTAACAGAACAGGACAACCTTACATTTTGCAAACAAATGTATTCACCGGTGGCAAAGGCGATAGAGAACAAAGAATCTATCTCTGGTTTGATCCTACCAAAGAATACCACAGATATTCAGTTCTATGGAACTTGTATCAAATTGTATTCTTTGTGGACAACATCCCAATTAGGGTGTTCAAGAACAACAAGCGCATGGGTGTGAAATTCCCATTTAACCAACCAATGAAGATCTACAACAGCCTATGGAACGCAGATGATTGGGCCACAAGGGGTGGTTTGGAGAAAACAGATTGGTCGAAAGCACCATTCATAGCTTCCTACAAAGGCTTCCACATTGATGGCTGTGAAACCTCCGTTGAAGCCAAGTTCTGTTCCACTCAAGGTAAGAGATGGTGGGACCAACAAGAATTCAGAGACCTTGATTCTTATCAATGGAGGAGGCTTAGATGGGTCAGAAGGAGATTCACCATTTATAATTATTGCAGTGATAGAACAAGGTACCCTCAGATGCCACCTGAATGCAGAACAAACGGTGACTATTGA

*>AhXTH53*

ATGCCATCATTGCTTTCCTCTTCTCTCCTTCAAATGCTTCCTCTTCTATTCTCTCTTATAATCTCACTCATGCTTTGTGGAATCATTGCTGATGAGTCTCCACCTTCACCTGGCTACTACCCTAGTTCCCAAGTTAGTTCTGTTGCCTTTGATCAAGCTTATAGAAACCTTTGGGGACCTCAGCACCAAAGACTAGACCAATCTGGCTCACTAACTATTTGGCTTGACTCTTACTCTGGAAGTGGATTCAAGTCAATCCGGCCGTATCGATCCGGATACTTTGGTGCTGCCATTAAGCTTCAATCTGGTTACACTGCAGGACTTTCAAACAACCAAGACTACCCAGGAGACCATGATGAAGTGGACATTGAGTTCCTTGGTACCATCCCAGGTAAGCAATATGTGCTGCAGACAAATGTGTTCATGAGAGGAAGCGGAGACAAGAATAATGTGATAGGAAGAGAGATGAGGTTTCACCTTTGGTTCGATCCAACACAAGATTTTCACCACTATGCTATTCTATGGACACCCACTGACATCATATTTTTGGTGGACGATGTTCCAATAAGGAATTACCCAAGAAAGAATGATGCAACATTCCCTGAAAGAGCAATGTACGTGTACGGATCAATATGGGATGCATCATCATGGGCCACAGAGAATGGTAAATACAAAGCTGATTACAAATACCAACCATTCATTGGAAGATACAAAGATTTCAAGCTCCAAGGTTGCACCACTCAATCTTCCTCCTCATGCCAGCCACCCTCACCTTCACCACCTGGCTACAATTCACTAAGTCCTCAACAGTATAATGCAATGCAATGGGTCCAAAACAATTACTTGGTCTATGACTATTGCCGTGATCCCAATAGAGACCATACCCTTACTCCAGAATGCTAA

*>AhXTH54*

ATGTCTAAGATGTCTAGTCTCTTGGGATTCTTTGTGGGTCTAGTTTTGGTGGGGGTTGTTGCCTCTTCCAAGTTTGAAGAACTCTACCAGCCTGCTTGGGCCTTCGATCATTTCATCCACGATGGCGAACTCATTAAACTCAAGCTTGATAACTATTCAGGTGCTGGTTTTGGATCAAAGAGCAAATATATGTTTGGGAAAGTGAGCATCCAACTTAAGCTTGTGGAGGGTGACTCTGCTGGAACCGTTACTGCTTTCTATATGTCATCGGAGGGTCCAAATCACAACGAATTTGATTTTGAGTTCTTGGGGAACACTACTGGTGAGCCTTATTCGGTGCAGACGAATGTGTATGTGAATGGTGTTGGTAACAGGGAGCAGAGACTCGACCTATGGTTCGATCCCACCAAGGACTTCCACACCTACTCTATCTTCTGGAATCAACGCCAAGTTGTGTTCCTAGTGGACGAGACGCCAATAAGGGTGCACACAAACCTTGAACACAAGGGAATCCCTTTCCCTAAAGACCAAGCAATGGGAGTGTACAGCTCAATATGGAACGCAGATGATTGGGCCACACAGGGTGGTAGGGTGAAGACAGATTGGAGCCATGCACCATTCGTTGCCACATACAAGGACTTCACGATTGACGCGTGTGAGTGCCCAGTGGGAGTGTCATCGTCATCAGTGGCCCCGGAAAATGCTAAGAGGTGCAGTAGCAGCGAGGATAAGAAGTATTGGTGGGATGAACCAACCATGTCGGAGTTGAACGTTCACCAGAGCCACCAGCTTATGTGGGTTAGGGCTAACCATATGGTCTATGACTATTGCACTGATACTGCTAGGTTCCCAGTCACACCCGCTGAGTGTGTCCACCACCGTCACTAA

*>AhXTH55*

ATGAGTTCTTTAATATTTGTTTTTCTTATTATTCTCTACTTTATTGATGGTGTCCTTGCTAGAAGAAGTAGGGAAGTAAGCTTTGATCAAAATTACAAGGTGATATGGGGTGATAATCATGTTGTGTCCTTAAACCAAGGGACAGAAATTCAGCTCTTAATGGATAACTCTTCAGGATCTGGCTTTGGATCCAAGAAGAACTATGGCTCTGGATTCTTTCATCTAAGGATCAAAGTACCAGGAGGCGATTCTGCAGGAGTTGTCACAGCCTATTATATGACTTCGCAAGGAAGCAGGCATGACGAGTTAGACTTTGAATTCTTGGGTAATAAAAAGGGGAAGCCGTACATATTACAAACCAATGTATTTTCAGAAGGCGAAGGGAACAGAGAGCAAAGATTTTTTGTGGACAATATCCCCATTAGGGTATTCAAGAACATGAGCAATATTGGAGTTAGGTACCCAACAAAGCCAATGCAAACACAAGCATCTTTGTGGGATGGAGATAGCTGGGCAACAGATGGAGGCAGAACCAAAATAAATTGGAACAGTGCACCTTTCAAAGCATATTTCCAAGGCTTTGATGTTAAAGGCTGCGAAGTTCTTCAAGATTCGTCAGATATTCAACATTGTGCTTCTCATAAGTACCAATGGAATACGCCAAGCTTTTGGCAGCTAGACCCTGTAAGACAAAGACAATATGAAAATGTGAAGACGAGATACATGATCTATGACTATTGTACTGATAGGAAGAGGAATCCCACACCTCCTTTGGAGTGTCAAAATTGA

*>AhXTH56*

ATGAGTTCTCTAATATTTGTTTTTCTTATTGTCCTTTTTACTGATGATGGTGTTCTTGTTAGAGGAGGAAATAGCGAAATAAACTTTGATCAAAATTACAAGGTGATATGGGGTGATAATCATGCTGTGTCTTTAAATCAAGGGACAGAAATTCAGCTCTTAATGGATAATTCTTCAGGAGCTGGGATTGGATCCAAGATGGACTATGGCTCCGGATTCTTTCATCTAAGGATCAAAGTACCAGGAAACGATTCTGCGGGAGTTGTCACGGCCTATTATATGAGTTCACAAGGAAGTTCAAGCAGGCACGACGAGCTAGACTTCGAATTATTGGGTAACAGAGAAGAGAAGCCGTACATATTACAAACCAATGTATTTGCAGATGACGGAGGAAATAGAGAGCAAAAGTTAAAGCTTTGGTTTGATCCTAGACAAGATTTCCACGACTACCAAATCCTTTGGAACCAACATCACATTGTCTTTTTTGTGGACAATATCCCCATTAGGGTATTCAAGAACATGAGTAATATTGGCGTTAGCTATCCAACAAAGCCAATGCAAATACATGCATCTTTGTGGGATGGAGATAGTTGGGCAACAGATGGAGGCAGAACCAAAATAAATTGGAGCAGTGCACCTTTCAAAGCATATTTCCAAGGCTTTGATGTTAAAGGCTGTGAAGTTCTTCAAAACTCATCAGATATTCAACATTGTGATTCTGATAAGTACCAATGGAATACCCCAAGCTTTTGGCAACTGGACCCTGTAAGACAAAGACAATATGAAGATGTGAAGACGAGATACATGATCTATGACTATTGTACTGATAGGAAGAGGAATCCCACACCTCCTTTGGAGTGCCAACATTGA

*>AhXTH57*

ATGAATAACTTTCACATAGCACTCTTCTTCCTCATTGGGATTGTGTCCTCCATTTTGTTTCAGATTTCAGTTGCATCTGTTGTTTCAACAGGAAACTTCAATAAGGACTTCTATGTTTTATGGTCACCTACCCATGTAAACACATCTGCTGATGGACACACTAGAACTTTGAAGCTTGATCAACAATCCGGGGCTGGTTTTGCTTCAAATCAGATGTTTTTGTTTGGACAAATTGACATGCAAATCAAACTAGTACCAGGTGATTCTGCAGGCACAGTATTAGCCTACTATATGGCATCTGATCAACCAAATCGCGACGAGATTGACTTTGAGTTTCTAGGAAACATGTCTGAGCAGCCTTATATTCTTCAAACAAATATTTATGCAGATGGGTTTGGCAATAGAGAGGAGAGGATTTATCTATGGTTTGATCCTACAAAGGACTTCCATACTTACTCAGTGTTGTGGAATCTGCACCAGATTGTGTTCATGGTGGATAGCATTCCAATAAGAGTGTACAGAAACCATGGTGACAAGGGAGTTCCATTTCCAAGAAGGCAACCAATGAGTCTAGAAGCAACTCTTTGGAATGGTGATAGCTGGGCAACAAGAGGAGGTCAAGACAAGATAGATTGGACAAAGGGTCCCTTCATAGCTTCATTCAGGAACTACAACATTGATGCTTGTGTGTGGAAAGGGAACCCAAGGTTCTGCAGAGTAGCTAGCCATGTTAATTGGTGGAACCTAAACAACTTCAGCACACTCACATCCCCACAAAGAAGGTGGTTCAAATGGGTCAGGAAATACCATATGATTTATGATTATTGCCAAGATAATGAGAGGTTCCAAAACAATCTTCCACAGGAATGTTCCCTTCCCAAGTATTGA

*>AhXTH58*

ATGGGAGGGTGTCATGTGTGTTTTCTCTTCTTGTGTTTGTCCTCAGCCATGGTTGTTGTTTCTGGTTCTTCTTCCATGAACAATAATTTGCCTATCATAGCCTTTGAGGATGGTTACACACCCTTGTTTGGAGATAATAATTTGATAATCCATAATGATGGCAAATTGGTTCATCTTACACTTGATCAGAGAACAGGTTCTGGATTTGTGTCCCATGAACTTTACCTTCATGGGTATTTTAGTGCTAAGATTAAGTTGCCTGCTGATTACACTGCTGGAATGTCAAATGGTGACATGTTCAAGAAGAACCATGACGAAATAGACTTTGAGTTTTTGGGAAATATTAGAGGCAGAGATTGGAGAATGCAGACCAATTTTTATGGTAATGGTAGTACCAACACTGGCAGAGAAGAAAGATATGATCTCTGGTTTGATCCTTCTCAAGATTTTCACCAGTACAGTATTCTGTGGACAGATTCTAAGATCATAGAAGTGAAGAGAACAGAATCTATGGGTGGAGATTTTCCTTCAAAGCCAATGACTTTGTATGCAACCATATGGGATGCATCTGATTGGGCAACTGATGGAGGAAAATACCGAGTAAATTACAAATATGCCCCTTATGTTGCTGAGTTCTCAAACTTTGTCATGCATGGTTGTGGGGTTGATCCAATTGATGAGAATGTTGCAATGTGCAACAATGCTCAAAATTCTAAGGCAAATATCATACCTAAGCACAAAATCAAGATGGAGAACTTTAGGAACAATCATATGACATACTCTTATTGTTATGATAGGGCTAGGTACCAAGTTCCTCCACCTGAGTGTGTCATTAGTCTCCAAGAGGCTGAAGCACTAAGAAAACTTGATCCTGCCACCTTCGGTGACGGTCGGCGCCACCGGGGTGGTGGAAAGCGGCGGCACCATCAAAGCAAAGGAAGGAAGGCAGAGGATGCTTCATTTTGA
