# Supplementary material for: Genome-wide identification of xyloglucan endotransglucosylase/hydrolase gene family members in peanut and their expression profiles during seed germination
Source: PeerJ. 2022 May 17;10:e13428. doi: 10.7717/peerj.13428 (PMC9121870; doi:10.7717/peerj.13428)
Supplement: Supplemental Information 4 [file peerj-10-13428-s004.docx]

**Supplementary File 4 Protein sequences of Peanut AhXTHs.**

>AhXTH1

MAPSWGGSYFLFLCMLLGFSTIAYGGNFSTDFDLLFGDDRVKISDGGQSMSLAMDKYSGSGVATKDQFLFGRFDMKIKLVGGNSAGTVTAFYLSSTGDHHDEVDLEFLGNLTGDPYMLSTNVFANGVGGREVQYYLWFDPTADYHTYSIDWSPTRIILWVDDTPIRVINNKEIIGVPFPTKQPMRLYTTLWNGDAWATRWGQVKIDLSQAPFIARFKNYNGTACVPKKGIADCKGFGASMKRGLDNENKKKLKQVNSKWVVYHYCRDLRRYAHGLPFECRRDNMAHSDNNQ

>AhXTH2

MATFYPLIRNGGGSLFLILLSWVFLVSSLLCVLGRPATFEQDFRATWSESHIRHTDQGRTIQLMLDRSSGCGFASKVKYMFGRVSMKIKLVPGDSAGTVTAFYMNSDTDSVRDELDFEFLGNRSGQPYTVQTNVYAHGKGDREQRVNLWFDPSADFHTYTILWNHKHIVFSVDDFPIRVYKNNEAKGVPYPKMQAMGVYSTLWEADNWATRGGLEKIDWSKAPFNAYYKDFDIEGCSVPGPASCASNPSNWWEGAAYQELSAIQARRYRWVRINHVVYDYCQDKSRFPHFALMFVGKKPNMEEHKRI

>AhXTH3

MHIIDHSYTSLGFFCFLLSLYTYLPPIFQPNQIKGTIINMGIEVLIINIVLLIGFAVAASAGNFNQDFEITWGNDRAKVLNNGQLLTLSLDKASGSGFRSRNEYLFGKIDMQLKLVPGNSAGTVTAYYLSSLGDTHDEIDFEFLGNLSGEPYTLHTNVFTQGKGNREQQFHLWFDPTKDFHTYSLLWNPQSIIFSVDGTPIREFKNWESKGVPFPKNQAMRIYSSLWDAEDWATRGGLVKTDWTQAPFTASYKGFNAQACVWTSSSGSSCSSKQGQSWFTQSLDSTGQARIQWVQKNYMIYNYCTDTKRFPQGLPPECTLA

>AhXTH4

MAAACVNNNNDALIVIVITLFLIITLPSSSMAGSNFNQQVDITWGDGRGKILNSGKILTLSLDRASGSGFQSKNEYLYGKIDMQIKLVPGNSAGTVTAYYLRSDGISWDEIDFEFLGNLSGDPYVVHTNVYTQGKGGKEQQFYLWFDPTANLHTYSILWNPAHIVFYIDGRPIREFKNLEGVGVSYPKNQPMKLYSSIWNADDWATRGGLVKTDWSQAPFTASFKNLKANGCVWSNGVSSCNLSSSNSSNNNNSWLSQQLDSNGQRKLKWVQKNYMIYNYCSDINRFPQGLPLECTLRTT

>AhXTH5

MLLPYPTSVVMVALFTMTMMMMLCGGDLHKNIDITWGNGRAQMLNNGQLLTVSLDAASGSGFQSKDHYLFGHFQIQLKLVPGNSAGTVTSFYLQSEGSTWDEIDFEFLGNLSGNPYILHSNVITQGKGGREQQFYLWFDPTSDFHTYSILWNPLCIILYVDGIPIREFKNYESRNISFPMKKPMRMYGSLWDAEDWATRSGLIKTNWSAAPFIAYFKNFFVNACVESTPPSITSSCTHPNKKSNNHNSVGEKWITQGLKPSEVDKLNWVHKNFIVYNYCSDLKRFPQGLPLECIPSVQKEKLLDQEFDITWGDGRAKMLNNGELLTLSLDKASCSGFQSKNEFLFGKIDMQLKLVPGNSLSSKGSNWDEIDFEFLGNVSGEPYILHTNVFSNGKGNTEQQFYLWFDPSADFHTYSILWNPQRIMFSVDSSPIREFKNMNQTEFHSQSRSQ

>AhXTH6

MAASTLSYLLLIPLLMVVAYAGNLDQEFDITWGDGRAKMLNNGELLTLSLDKASGSGFQSKNEYLFGKIDMQLKLVPGNSAGTVTAYYLSSKGTTWDEIDYEFLGNLSGDPYILHTNVFSQGKGNREQQFYLWFDPTADFHTYSITWNPQRIIFSVDGTPIREFKNSESMGVPFPKSQPMRIYSSLWNADDWATRGGLVKTDWTKAPFTASYRNFNADACIWSNGASSCGSGSGSSSTSSSWLSQELDTTAQERLRWVQKNYMIYNYCTDAKRFPQGFPPECRTS

>AhXTH7

MADPVLHPDTTNPLHHHQTQPLKEIAIDYTPEACSHCPNSNTITLTFDHRGGARWRTTTRFHYGTFSSLIQCPKGNTNGLNFNLYLSSLEGEKSQDEIDFEFLGKDRTIVQTNYFSGGNGNKEKIHHLGFDASDGFHEYVIKWSYDVIEWLIDGKVVRREEKKEGKGFPQKPMFLYASIWDASCIDNGRWAGKYDGSDAPYVCLYKDIHVPTSTAVK

>AhXTH8

MMKALLLFLFAISSSLFMEPCYSSSGYWPPSPGYWPSHKFRSMNFYKGFRNLWGPQHQALDNNNALTIWLDRTSGSGFKSVRPFRSGYFGASIKLHPGYTAGVITAFYLSNNEAHPGFHDEVDIEFLGTTFGKPYTLQTNVYIRGSGDGTIIGREMKFHLWFDPTKDFHHYAILWSPKEIIPMWVYGSIWDASSWATEDGKYKADYRYQPFVARYTNFKASGCSAYASRWCHPVSASPYRSGGLTRQQYWAMRWVQRHHMVYNYCQDPKRDHRLTPECWGKKKENNEKRMRSRGYVIVHIYASYFIILLLTVSSETFQLVSFISFTPRNLQVQN

>AhXTH9

MFTTMLPSSPLSFFFFFFLLVLSFMICASAQGPPSPGYYPGSKISPISFDQGFRNLWGPQHQRLEQGTLSIWLDSNSGSGFKSLHSYQSGYFSAAIKLQPGYTAGVITTLYLSNNQDHPGNHDEIDIEFLGTTPDKPYVLQTNVYIRGTGDGNIVGREMRFHLWFDPTQDFHIYAILWKPSETIFFVDDVPIRRYPRKGDATYPNRPMYVYGSIWDASSWATEDGKYKANYKYQPFIGRYKNFKLQGCTINESPSSCKPPSASPSGYGSLSLQQISAMQWVQNHYLVYYYCHDPKRDHTLTPEC

>AhXTH10

MSKEVSLFLGLVMGFVFVGVAFAAATAKFEELFQPSWALDHFIHEGDLLKLKLDNYSGAGFVSKSKYMFGKVTVQLKLVEGDSAGTVTAFYMSSEGPNHNEFDFEFLGNTTGEPYSVQTNVYVNGVGNREQRLNLWFDPTKDFHSYSFFWNQRQVIFLVDETPIRVHTNMEHRGIPFPKDQAMGVYSSIWNADDWATQGGRVKTNWSHAPFIATYKAFEINGCECPIVSSTSVENLKRCSSNEKKYWWDEPNLGVLSLHQSHQLMWVRAKHMVYDYCADTARFPVMPAECVHHSHHKLVLKN

>AhXTH11

MTTLVTKVPIGFLSLLLIITIATKAAAGNFYQDFEVTWGDNRAKIFNGGQLLTLSLDRASGSGFRSKNEYLFAKLDMQIKLVPGNSAGTVTTYYLSSSGGTHDEIDFEFLGNLSGDPYILHTNVFTQGKGNREQQFYLWFDPTADFHTYSILWNPHNIIFSVDGTPIREFKNLESRGVPFPKNQPMRLYSSLWNADDWATRGGLVKTDWAHAPFTASYRNFNAPPAQSWMGQSLDSTGLARIHWVQRNYMIYNYCTDLKRFPQGPPLECSLA

>AhXTH12

MALIWQLKLLFSPLLIILLANVVSSRTRPFTAPTVTPLTNSFPRVPIDPNFSNAFGASNIKLLANGTMATLALDKLSGSGLVSKSSYYYGFFSAAIKLPAGISSGVVVAFYLSNADKFPHNHDEIDIELLGHDKRNDWVIQTNVYANGSVSTGREEKFYFWFDPTKQYHYYSILWNSYHTVFLVDNIPVREFIHGSVFPSKPMSVYATIWDGSEWATHGGKYPVDYKYAPFVVSFSEMQLAGCTSDPLACSKSTPSSGVDPVNGPQFTKLSPQQMAALDWARKKLMFYSYCTDKNRYKVMPPECH

>AhXTH13

MGLGGGLVTFFLCLLFFAAPSASSTNLLPIIPFDEGYAPLFGDNNLVIHRDGKTVHLSLDERTGSGFVSHDLYLHGYFRASIKLPADYTAGVVVAFYMSNGDMFQNNHDEIDFEFLGNIRGKDWRIQTNVYGNGSTSIGREERYGLWFDPAEDFHQYSILWTDSQIIFYVDNVPIREIKRTESMGGDFPSKPMTLYATIWDASDWATNGGKYRVNYKYAPYVAEFSDLVLHGCAVDPIEHEAKCDNAQTSKAVPTGVTPAQRIKMENFRKKHMTYSYCYDKVRYKVPPTECVINPQEAERLRKFDPVTFGGGRRRHGKRHYRSRGSQAEEAAAF

>AhXTH14

MGSTCTNHNGFYVGIMVIGLVVVSTMVGSCEGNFNQEFDLTWGGNRAKIFGGGQLLSLSLDRVSGSGFQSKREYLFGRIDMQLKLVAGNSAGTVTAYYLSSQGPTHDEIDFEFLGNLSGDPYTLHTNIFTQGKGDREQQFHLWFDPTKNFHTYSVIWKPQHIIFLVDNIPIRVFKNAESMGVPFPKKQPMRIYSSLWNADDWATRGGLVKTDWAKAPFTAYYRNFRATQLSSASLRPNTRSSEWETSDIDAIGRRRLRWVQKYFMIYNYCNDFKRFPQGLPAECRSRF

>AhXTH15

MGGSTTKGFNLLVVGVVVVVTTMVGTCSANFYDDFDLTWGENRAKIFNGGQLLSLSLDKVSGSGFRSKKEYMFGRIDMQLKLVSGNSAGTVTAYYLSSEGATHDEIDFEFLGNVSGEPYILHTNVFSQGKGNREQQFYLWFDPTTNFHTYSIIWNPHHITFLVDNIPIRVFKNAESDGVPFPKNQPMRIYSSLWNADDWATRGGLVKTDWSKAPFTAYYRNFKVTGFSATSAFSDVATSEIQNSGKDSVLDAYGRRRLRWVQKYFMVYNYCNDLKRFPEGIPAECSHGRF

>AhXTH16

MDHNNTLRRFGEIIPKTHRNHNFLLSLFLFFFFFFFFFFSSHAAFDLATIPFNDGYSPLFGDSNVVRSDDGNGVNLLLDRFTGSGFISSSMYKYGFFSANIKLPSNYTAGICVAFYTSNGDVFEKTHDELDFEFLGNLAGKPWRFQTNLYGNGSTHRGREERYRLWFDPTKEYHRYSILWTAKNVVFYIDEVPIREVLRSAEMGADYPSKPMSLYATIWDASNWATSGGKYKVNYKYAPFVAEFKDLVLKGCSVDPIEESTVAGRSICSDQHADLEAQDYAAVTPRRRLAMRRFRQRYMYYSYCYDTLRYPNPLPECDIIPSEKQRFKETGRLKFGGSHRRQSRRKGRTTTPVDDTDQGDM

>AhXTH17

MYMAFFKNPFFLLLSLWALVLSGVCVWGKPVTFLQDFRVTWSDSHIRQIDNQGTAIQLILDRNSGCGFASKSRYMFGRISMKIKLIAGDSAGTVTAFYMSSDTDAIRDELDFEFLGNRSGQPYTVQTNIYAHGKGGREQRVNLWFDPSAQFHTYTILWNHHHIVFYVDEFPIRVYKNDVARGVAYPRMQGMGVYSTLWEADNWATRGGLEKIDWRKAPFYAYYKDFDIEGCQLPGPTSCASNASNWWEGAAYQALTPTQARLYRWVRINHIIYDYCQDKPRFPLGPPPECLS

>AhXTH18

MVRMEAKASSSLGVILLLVVIAEAAVSKGSFEDNFSIMWSEDHFSTSKDGQIWYLSLDKDTGCGFQTKQRYRFGWFSMKLKLVAGDSAGVVTAYYMCSENGAGPERDELDFEFLGNRTGQPFLIQTNVYKNGTGGREMRHMLWFDPTEDYHTYSILWNNHQIVFFVDRVPIRVFKNNGKENNFFPNEKPMYLFSSIWNADDWATRGGLEKTNWKLAPFVSSYKDFSVDGCQWKDPYPACVSTTTDNWWDQYSAWHLSDDQKKDYAWVRRNLVIYDYCNDSERYPTLPEECSLSPWD

>AhXTH19

MAAASEKMFLALLFIFFMARGIIIVDANFGKSMYLTWGTQHASIQGEDLQLVLHQTSGSAAQTKIPFLFGSIESKIKLVPNNSAGTVTAYYLSSTGSQHDEIDFEFLGNISGQPYIVHTNIYTQGNGSKEQQFYLWFDPTADFHNYTIHWNPTQVVWYIDSIPIRVFMNNEEEGIAYPNKQGMKVYTSLWNADDWATRGGLVKTNWTNAPFIARLNRFRARACKWNGPISINNCASNVPSNWWTSPIYKQLSYAQMGQLYWVRNNYMIYDYCKDTKRFNGQSSDFVI

>AhXTH20

MALFVFAILMLMAPSSNAEWPPSPGYWPSSKFKTMNFYKGFRNLWGPQHQRIEQNALTIWLDRTSGSGFKSVAPFRSGYFGASIKLQPGYTAGVITAFYLSNNEAHPGFHDEVDIEFLGTTFGKPYTLQTNVYIRGSGDGQIIGREMKFHLWFDPTKDFHHYAILWSPKEIIFFVDDVPIRRYPRKSAETFPMRPMWLYGSIWDASSWATEDGKYKADYRYQPFVARYTNFKASGCTAYAPRWCHAVSASPYRSGELSRQQYSAMRWVQMYHMVYNYCQDSKRDHRLTPECWS

>AhXTH21

MGSRRVGVLTLSLVIVASLVSAAMCGVPRRPVDVQFGRNYVPTWAFDHIKYFNGGSEIQLHLDKYTGTGFQSKGSYLFGHFSMYIKMVPGDSAGTVTAFYLSSQTAEHDEIDFEFLGNRTGQPYILQTNVFTGGKENRESISGLIPPKNTTDIQFFVDDVPIRVFKNCKDLGVKFPFDQPMKIYNSLWNADDWATRGGLEKTDWSKAPFIASYKGFHIDGCEASVEAKFCSTQGKRWWDQQEFRDLDALQWRRLRWVRQKFTIYNYCNDRKRYPTLPPECSRDRDI

>AhXTH22

MGYYSMWSVCCVILGWFVSVGICGTPRRPMAVPFGRNYVPTWAYDHIKYLNSGYDAQLLLDKYTGTGFQSKGSYLFGHFSMDIKMVAGDSAGTVTAFYLSSLGTEHDEIDFEFLGNRTGQPYILQTNVFTGGKGDREQRIYLWFDPTKEYHRYSVLWNLYQIVFFVDNIPIRVFKNNKRMGVKFPFNQPMKIYNSLWNADDWATRGGLEKTDWSKAPFIASYKGFHIDGCETSVEAKFCSTQGKRWWDQQEFRDLDSYQWRRLRWVRRRFTIYNYCSDRTRYPQMPPECRRNGDY

>AhXTH23

MPSLLSSSLLQMPPLLFSLILSLMLCGIIADESPPSPGYYPSSQVSSVAFDQAYRNLWGPQHQRLDQSGSLTIWLDSYSGSGFKSIRPYRSGYFGAAIKLQSGYTAGLSNNQDYPGDHDEVDIEFLGTIPDKQYVLQTNVFMRGSGDKNNVIGREMRFHLWFDPTQDFHHYAILWTPTDIIFLVDDVPIRNYPRKNDATFPERAMYVYGSIWDASSWATENGKYKADYKYQPFIGRYKDFKLQGCTTQSSSSCQPPSPSPPGYNSLSPQQYNAMQWVQNNYLVYDYCRDPTRDHTLTPEC

>AhXTH24

MSKMSSLLGFFVGLVLVGVVASSKFEELYQPAWALDHFIHDGELIKLKLDNYSGAGFGSKSKYMFGKVSIQLKLVEGDSAGTVTAFYMSSEGPNHNEFDFEFLGNTTGEPYSVQTNVYVNGVGNREQRLDLWFDPTKDFHTYSIFWNQRQVVFLVDDTPIRVHTNLEHKGIPFPKDQAMGVYSSIWNADDWATQGGRVKTDWSHAPFVATYKDFTIDACECPVGVSSSSVAPENAKRCSSSEDKKYWWDEPTMSELNVHQSHQLMWVRANHMVYDYCTDTARFPVTPAECVHHRH

>AhXTH25

MNNLQIALFFLIGIVSSILFHISVASVVSTGNFNKDFYVLWSPTHVNTSADGHTRTLKLDQQSGAGFASNQMFLFGQIDMQIKLVPGDSAGTVLAYYMASDQPNRDEIDFEFLGNMSEQPYILQTNIYADGFGNREERIYLWFDPTKDFHTYSVLWNLHQIVFMVDSIPIRVYRNHGDKGVPFPRRQPMSLEATLWNGDSWATRGGQDKIDWTKGPFIASFRNYNIDACVWKGNPRFCRVASHVNWWNLNNFSTLTSPQRRWFKWVRKYHMIYDYCQDNERFQNNLPQEWSLPKY

>AhXTH26

MGGCHLCFLFLCLSSAMVVVSGSSSMNNNLPIIAFEDGYTPLFGDNNLIIHNDGKLVHLTLDQGTGSGFVSHELYLHGYFSAKIKLPADYTAGVVVAFYMSNGDMFKKNHDEIDFEFLGNIRGRDWRMQTNFYGNGSTNTGREERYDLWFDPSQDFHQYSILWTDSKIIFYIDNVPIREVKRTESMGGDFPSKPMTLYATIWDASDWATDGGKYRVNYKYAPYVAEFSNFVMHGCGVDPIDENVAMCNNAQNSKANIIPKHKIKMENFRNNHMTYSYCYDRARYQVPPPECVISLQEAEALRKLDPATFGGGRRHRGGGKRRHHQSKGRKAEDASF

>AhXTH27

MAPSWGGSYFLLLCMLLGFSTIAYGGNFNTDFDLLFGDDRVKISDGGQSMSLSMDKYSGSGVATKDQFLFGRFDMKIKLVGGNSAGTVTAFYLSSQGDHHDEVDLEFLGNLTGDPYMLSTNVFANGVGGREVQYYLWFDPTADYHTYSIDWSPTRIIIWVDDTPIRVINNKEIIGVPFPTKQPMRLYTTLWNGDAWATRWGQVKIDLSQAPFIARFKNYNGTACVPKKGIADCKGFGASMKKGIDNENKKKLKQVNSKWVVYHYCRDLRRYAHGLPFECRRDNMAHSDNNQ

>AhXTH28

MATFYPLIRNGGGSLFLILLSCVFLVSSLLCVLGRPATFEQDFRATWSESHIRHIDQGRTIQLMLDQSSGCGFASKVKYMFGRVSMKIKLVPGDSAGTVTAFYMNSDTDSVRDELDFEFLGNRSGQPYTVQTNVYAHGKGNREQRVNLWFDPSADFHTYTILWNHKHIVFSVDDFPIRVYKNNEAKGVPYPKMQAMGVYSTLWEADNWATRGGLEKIDWSKAPFNAYYKDFDIEGCSVPGPASCSSNPSNWWEGAAYQELSAIQARRYRWVRINHVVYDYCQDKSRFPVTPPECLAGI

>AhXTH29

MAASTLSYLLLIPLLMVVAYAGNLDQEFDITWGDGRAKMLNNGELLTLSLDKASGSGFQSKNEYLFGKIDMQLKLVPGNSAGTVTAYYLSSKGTTWDEIDYEFLGNLSGDPYILHTNVFSQGKGNREQQFYLWFDPTADFHTYSITWNPQRIIFSVDGTPIREFKNSESMGVPFPKSQPMRIYSSLWNADDWATRGGLVKTDWTNAPFTASYRNFNADACIWSNGASSCGSGSGSSSTSSSWLSQELDTTAQERLRWVQKNYMIYNYCTDAKRFPQGFPPECRTS

>AhXTH30

MASSKVVVLVVPLLVMSFCMVSCWGGNFNKDFQITWGDGRAKILNNGNLLTLSLDKASGSGFQSTNEYLFGKIDMQLKLVPGNSAGTVTAYYLSSKGATWDEIDFEFLGNLSGDPYILHTNVFSQGKGNREQQFYLWFDPTADFHTYSILWNPQRIVFSVDGTPIREFKNLESAGVPFPKNQPMRIYSSLWNADDWATRGGLVKTDWSKAPFTASYRNFNANNACIWKNGRSSCKSSSSWLSQELDSTGLQRLRWVQKNYMIYNYCTDKKRFPRGFPIECNRS

>AhXTH31

MNPTSLPKKQFPDSQLLDQEFDITWGDGRAKMLNNGELLTLSLDKASCFGFQSKNEFLFDKIDMQLKLVSGNSAGTVTAYYLSSKGSNWDEIDFEFLGNVSGEPYILHTNVFSNGKGNREQQFYLRFDPTTDFHTYSILWNPQLIMFSVDSSPIRQ

>AhXTH32

MLLPYPTSVVMVALFSMTMMMMLCGGDLHKKIDITWGNGRAQMLNNGQLLTVSLDAASGSGFQSKDHYLFGHFQIQLKLVPGNSAGTVTSFYLQSEGSTWDEIDFEFLGNLSGNPYILHTNVITQGKGGREQQFYLWFDPTSDFHTYSILWNPLCVILYVDGIPIREFKNYESRNISFPMKKPMRMYGSLWDAEDWATKGGVIKTNWSAAPFIAYFKNFFVNACVESTPPSIKSSCTHLNKKNNNHNSVGEKWITQELKPSEVDKLNWVHKNFMVYNYCSDLKRFPQGLPLECKIN

>AhXTH33

MVASCVNNNNDALIVIVITLFLILPPSSMAGSNFNQQVDITWGDGRGKILNSGKILTLSLDRASGSGFQSKNQYLYGKIDMQIKLVPGNSAGTVTAYYLRSDGISWDEIDFEFLGNLSGDPYVVHTNVYTQGTGGREQQFYLWFDPTANLHTYSILWNPAHIVFYIDGRAIREFKNLEGVGIPYPKNQPMKLYSSLWNADDWATRGGLVKTDWSQAPFTASFRNLKANGCVWSNGVSSCNLSSNSSDNNNSWLSQQLDSNGQRKLKWVQKNYMIYNYCSDINRFPQGLPLECTLLTT

>AhXTH34

MGIEVLIINSVLLIGFAVAASAGNFNQDFEITWGNDRAKVLNNGQLLTLSLDKASGSGFRSRNEYLFGKIDMQLKLVPGNSAGTVTAYYLSSLGDTHDEIDFEFLGNLSGEPYTLHTNVFTQGKGNREQQFHLWFDPTKDFHTYSLLWNPQSIIFSVDGTPIREFKNWESKGVPFPKNQAMRIYSSLWDAEDWATRGGLVKTDWTQAPFTASYKGFNAQACVWSSSSGSSCSSKQGQSWFTQSLDSTGQARIQWVQKNYMIYNYCTDTKRFPQGLPPECTLA

>AhXTH35

MASSSPSLLLIPLLVGSIMVVAYGAKLDQEVDITWGDGRAKMLNNGELLTLSLDKASGSGFQSKNEYLFGKIDMQLKLVSGNSAGTVTAYYLSSKGATWDEIDYEFLGNLSGDPYILHTNVFSQGKGNREQQFYLWFDPTADFHTYSITWNPQRIIFSVDGTPIREFKNSESIGVPFPKSQPMRIYSSLWNADDWATRGGLVKTDWTKAPFTASYRNFNADACIWSNGASSCGSGSGLGSSSTTSSSSWLSQELDTTAQERLRWVQKNYMIYNYCTDAKRFPQGFPPECRTS

>AhXTH36

MADPVLHPDTTNPLHHHQTQPLKEIAIDYTPEACSHCPNSNTITLTFDHRGGARWRTTTRFHYGTFSSLIQCPKGNTNGLNFNLYLSSLEGEKSQDEIDFEFLGKDRTIVQTNYFSGGNGNKEKIHHLGFDASDGFHEYVIKWSCDVIEWLIDGKVVRREEKKEGKGFPQKPMFLYASIWDASCIANGMWAGKYDGSDAPYVCLYKDIHVPTSTAVK

>AhXTH37

MMKALLLFLFAISSSLFMEPCYSSSGYWPPSPGYWPSHKFRSMNFYKGFRNLWGPQHQALDNNNALTIWLDRTSGSGFKSVRPFRSGYFGASIKLHPGYTAGVITAFYLSNNEAHPGFHDEVDIEFLGTTFGKPYTLQTNVYIRGSGDGTIIGREMKFHLWFDPTKDFHHYAILWSPKEIIPMWVYGSIWDASSWATEDGKYKADYRYQPFVARYTNFKASGCSAYASRWCHPVSASPYRSGGLTRQQYWAMRWVQRHHMVYNYCQDPKRDHRLTPECWGKKKENNEKRMRSRGYVIVHIYASYFIILLLTVSSETFQLVSFISFTPRNLQVQN

>AhXTH38

MGSTCTNHNGFYVGIMVIGLVVVSTMVGSCEGNFNQEFDLTWGGNRAKIFGGGQLLSLSLDRVSGSGFQSKREYLFGRIDMQLKLVAGNSAGTVTAYYLSSQGPTHDEIDFEFLGNLSGDPYTLHTNIFTQGKGDREQQFHLWFDPTKNFHTYSVIWKPQHIIFLVDNIPIRVFKNAESMGVPFPKKQPMRIYSSLWNADDWATRGGLVKTDWAKAPFTAYYRNFRATQLSSASLRPNTRSSEWETSDIDAVGRRRLRWVQKYFMIYNYCNDFKRFPQGLPAECRSRF

>AhXTH39

MSKEVSLFLGLVMGFVFVGVAFAAATAKFEELFQPSWALDHFIHEGDLLKLKLDNYSGAGFVSKSKYMFGKVTVQLKLVEGDSAGTVTAFYMSSEGPNHNEFDFEFLGNTTGEPYSVQTNVYVNGVGNREQRLNLWFDPTKDFHSYSFFWNQRQVIFLVDETPIRVHTNMEHRGIPFPKDQAMGVYSSIWNADDWATQGGRVKTNWSHAPFIATYKAFEINACECPIVSSKSVENLKRCSSNEKKYWWDEPNLGVLSLHQSHQLMWVRAKHMVYDYCADTARFPVMPAECVHHSHHKLVLKN

>AhXTH40

MLVALFICVVVLVGNIVQVDGNFSKSMYLTWGVQHASIMGEDLHLVLDTTSGSAAKSKRSFLFGSIEMLIKLIPGNAAGIVTAYYLSSTGSQHDEIDFEFLGNITGQPYTVNTNIYTQGKGNKEQQFYLWFDPAADFHNYTIHWNPTQIVWYVDGLPIRVFQNYENHGVAYPNKHGMRVYSSLWNADDWATRGGLVKTDWRGAPFIASFHHFRARACKWNGAVSINHCASNVPANWWISPLYKQLSYSEKGQLNWVRKNYMIYDYCADSKRFNGQLPPECSKTQL

>AhXTH41

MTTLVTKVPIGFLSLLLIITIATKAAAGNFYQDFEVTWGDNRAKIFNGGQLLTLSLDRASGSGFRSKNEYLFAKLDMQIKLVPGNSAGTVTTYYLSSLGGTHDEIDFEFLGNLSGDPYILHTNVFTQGRGNREQQFYLWFDPTADFHTYSILWNPHNIIFSVDGTPIREFKNLESRGVPFPKNQPMRLYSSLWNADDWATRGGLVKTDWAHAPFTASYRNFNAPPAQSWMGQSLDSTGLARIHWVQKNYMIYNYCTDLKRFPQGPPPECSLA

>AhXTH42

MALIWQLNLLFSPLIIICLLSNVVSSRTRPFTAPTVTPLTNSFPRVPIDPAFSNAFGASNVKLLANGTMATLALDKLSGSGLVSKSSYYYGFFSAAIKLPAGISSGVVVAFYLSNADKFPHNHDEIDIELLGHDKRNDWVIQTNVYANGSVSTGREEKFYFWFDPTKQYHYYSILWNSYHTVFLVDNIPVREFIHGTVFPSKPMSVYATIWDGSEWATHGGKYPVDYKYAPFVVSFSEMQLTGCTSDPLACSKSTPSSGVDPVNGPQFTKLSPQQLAALDWARKKLMFYSYCTDKNRYKVMPPECH

>AhXTH43

MGLGGGLVTFFLCLLFFAAPSASSTNLLPIIPFDEGYAPLFGDNNLVIHRDGKTVHLSLDERTGSGFVSHDLYLHGYFSASIKLPADYTAGVVVAFYMSNGDMFQNNHDEIDFEFLGNIRGKDWRIQTNVYGNGSTSIGREERYGLWFDPAEDFHQYSILWTDSQIIFYVDNVPIREIKRTESMGGDFPSKPMTLYATIWDASDWATNGGKYRVNYKYAPYVAEFSDLVLHGCAVDPIEHEAKCDNAQTSKAVPTGVTPAQRIKMENFRKKHMTYSYCYDKVRYKVPPTECLINPQEAERLRKFDPVTFGGGRRRHGKRHYRSRGSQAEEAAAF

>AhXTH44

MGGFNLLVVGVVVVVTTMVGTCSANFYDDFDLTWGENRAKIFNGGQLLSLSLDKVSGSGFRSKKEYMFGRIDMQLKLVSGNSAGTLSSEGATHDEIDFEFLGNVSGEPYILHTNVFSQGKGNREQQFYLWFDPTTNFHTYSIIWKPHHITFLVDNIPIRVFKNAESVGVPFPKNQPMRIYSSLWNADDWATRGGLVKTDWSKAPFTAYYRNFKATAFSATSAFSNVVTSEIQGSGEDSVLDAYGRRRLRWVQKYFMVYNYCNDPKRFPQGIPAECSHGRF

>AhXTH45

MDHNNTLRRFGEIIPKTHRNHNFLLSLFLFFFFFFFFFFSSHAAFDLATIPFNDGYSPLFGDSNVVRSDDGNGVNLLLDRFTGSGFISSSMYKYGFFSANIKLPSNYTAGICVAFYTSNGDVFEKTHDELDFEFLGNLAGKPWRFQTNLYGNGSTHRGREERYRLWFDPTKEYHRYSILWTAKNVVFYIDEVPIREVLRSAEMGADYPSKPMSLYATIWDASNWATSGGKYKVNYKYAPFVAEFKDLVLKGCSVDPIEESTVAGRSICSDQHADLEAQDYAAVTPRRRLAMRRFRQRYMYYSYCYDTLRYPNPLPECDIIPSEKQRFKETGRLKFGGSHRRQSRRKGRTTTPVDDTDQGDM

>AhXTH46

MYMAFFKNPFFLLLSLWALVLSGVCVWGKPVTFLQDFRVTWSDSHIRQIDNQGTAIQLILDRNSGCGFASKSRYMFGRISMKIKLIAGDSAGTVTAFYMSSDTDAIRDELDFEFLGNRSGQPYTVQTNIYAHGKGGREQRVNLWFDPSAQFHTYTILWNHHHIVFYVDEFPIRVYKNDVARGVAYPRMQGMGVYSTLWEADNWATRGGLEKIDWRKAPFYAYYKDFDIEGCQLPGPTSCASNASNWWEGAAYQALTPTQARLYRWVRINHIIYDYCQDKPRFPLGPPPECLS

>AhXTH47

MVRMEAKASSSLGVILLLVVIAEAAVSKGSFEDNFSIMWSEDHFSTSKDGQIWYLSLDKDTGCGFQTKQRYRFGWFSMKLKLVAGDSAGVVTAYYMCSENGAGPERDELDFEFLGNRTGQPFLIQTNVYKNGTGGREMRHMLWFDPTEDYHTYSILWNNHQIVFFVDRVPIRVFKNNGKENNFFPNEKPMYLFSSIWNADEWATRGGLEKTNWKLAPFVSSYKDFSVDGCQWKDPYPACVSTTTDNWWDQYSAWHLSDDQKKDYAWVQRNLVIYDYCNDSQRYPILPEECSLSPWD

>AhXTH48

MAAASEKMVLALLFIFFMARGIIIVDANFGKSMYLTWGTQHASIQGEDLQLVLDQTSGSAAQTKIPFLFGSIESKIKLVPNNSAGTVTAYYLSSTGSQHDEIDFEFLGNISGQPYIVHTNIYTQGNGSKEQQFYLWFDPTADFHNYTIHWNPTQVVWYIDSIPIRVFMNYEEEGIAYPNKQGMKVYTSLWNADDWATRGGLVKTNWTNAPFIARLNRFRARACKWNGPISINNCASNVPSNWWTSPIYKQLSYAQMGQLNWVRNNYMIYDYCKDTKRFNGQVPPECFKTQF

>AhXTH49

MMMKALLLFLFAISSSLFMEPCYSSSGYWPPSPGYWPSHKFRSMNFYKGFRNLWGPQHQALDNNNALTIWLDRTSGSGFKSVRPFRSGYFGASIKLHPGYTAGVITAFYLSNNEAHPGFHDEVDIEFLGTTFGKPYTLQTNVYIRGSGDGTIIGREMKFHLWFDPTKDFHHYAILWSPKEIIPMWVYGSIWDASSWATEDGKYKADYRYQPFVARYTNFKASGCSAYASRWCHPVSASPYRSGGLTRQQYWAMRWVQRHHMVYNYCQDPKRDHRLTPECWG

>AhXTH50

MLMAPSSNAEWPPSPGYWPSSKFKTMNFYKGSGFKSVAPFRSGYFGASIKLQPGYTAGVITAFYLSNNEAHPGFHDEVDIEFLGTTFGKPYTLQTNVYIRGSGDGQIIGREMKFHLWFDPTKDFHHYAILWTPKEIIFFVDDVPIRRYPRKSAETFPMRPMWLYGSIWDASSWATEDGKYKADYRYQPFVARYTNFKASGCTAYAPRWCHAVSASPYRSGGLSRQQYSAMRWVQTYHMVYNYCQDSKRDHRLTPECWS

>AhXTH51

MGSRRVGVLTLSLVVVASLVSAAMCGVPRRPVDVQFGRNYVPTWAFDHIKYFNGGSEIQLHLDKYTGTGFQSKGSYLFGHFSMYIKMVPGDSAGTVTAFYLSSQTAEHDEIDFEFLGNRTGQPYILQTNVFTGGKGDREQRIYLWFFVDDVPIRVFKNCKDLGVKFPFDQPMKIYNSLWNADDWATRGGLEKTDWSKAPFIASYKGFHIDGCEASVEAKFCSTQGKRWWDQQEFRDLDALQWRRLRWVRQKFTIYNYCNDRKRYPTLPPECSRDRDI

>AhXTH52

MGYYSLWSVCCVILAWFVSVGICSTPRRPMAVPFGRNYVPTWAYDHIKYLNSGYDAQLLLDKYTGTGFQSKGSYLFGHFSMDIKMVAGDSAGTVTAFYLSSLGTEHDEIDFEFLGNRTGQPYILQTNVFTGGKGDREQRIYLWFDPTKEYHRYSVLWNLYQIVFFVDNIPIRVFKNNKRMGVKFPFNQPMKIYNSLWNADDWATRGGLEKTDWSKAPFIASYKGFHIDGCETSVEAKFCSTQGKRWWDQQEFRDLDSYQWRRLRWVRRRFTIYNYCSDRTRYPQMPPECRTNGDY

>AhXTH53

MPSLLSSSLLQMLPLLFSLIISLMLCGIIADESPPSPGYYPSSQVSSVAFDQAYRNLWGPQHQRLDQSGSLTIWLDSYSGSGFKSIRPYRSGYFGAAIKLQSGYTAGLSNNQDYPGDHDEVDIEFLGTIPGKQYVLQTNVFMRGSGDKNNVIGREMRFHLWFDPTQDFHHYAILWTPTDIIFLVDDVPIRNYPRKNDATFPERAMYVYGSIWDASSWATENGKYKADYKYQPFIGRYKDFKLQGCTTQSSSSCQPPSPSPPGYNSLSPQQYNAMQWVQNNYLVYDYCRDPNRDHTLTPEC

>AhXTH54

MSKMSSLLGFFVGLVLVGVVASSKFEELYQPAWAFDHFIHDGELIKLKLDNYSGAGFGSKSKYMFGKVSIQLKLVEGDSAGTVTAFYMSSEGPNHNEFDFEFLGNTTGEPYSVQTNVYVNGVGNREQRLDLWFDPTKDFHTYSIFWNQRQVVFLVDETPIRVHTNLEHKGIPFPKDQAMGVYSSIWNADDWATQGGRVKTDWSHAPFVATYKDFTIDACECPVGVSSSSVAPENAKRCSSSEDKKYWWDEPTMSELNVHQSHQLMWVRANHMVYDYCTDTARFPVTPAECVHHRH

>AhXTH55

MSSLIFVFLIILYFIDGVLARRSREVSFDQNYKVIWGDNHVVSLNQGTEIQLLMDNSSGSGFGSKKNYGSGFFHLRIKVPGGDSAGVVTAYYMTSQGSRHDELDFEFLGNKKGKPYILQTNVFSEGEGNREQRFFVDNIPIRVFKNMSNIGVRYPTKPMQTQASLWDGDSWATDGGRTKINWNSAPFKAYFQGFDVKGCEVLQDSSDIQHCASHKYQWNTPSFWQLDPVRQRQYENVKTRYMIYDYCTDRKRNPTPPLECQN

>AhXTH56

MSSLIFVFLIVLFTDDGVLVRGGNSEINFDQNYKVIWGDNHAVSLNQGTEIQLLMDNSSGAGIGSKMDYGSGFFHLRIKVPGNDSAGVVTAYYMSSQGSSSRHDELDFELLGNREEKPYILQTNVFADDGGNREQKLKLWFDPRQDFHDYQILWNQHHIVFFVDNIPIRVFKNMSNIGVSYPTKPMQIHASLWDGDSWATDGGRTKINWSSAPFKAYFQGFDVKGCEVLQNSSDIQHCDSDKYQWNTPSFWQLDPVRQRQYEDVKTRYMIYDYCTDRKRNPTPPLECQH

>AhXTH57

MNNFHIALFFLIGIVSSILFQISVASVVSTGNFNKDFYVLWSPTHVNTSADGHTRTLKLDQQSGAGFASNQMFLFGQIDMQIKLVPGDSAGTVLAYYMASDQPNRDEIDFEFLGNMSEQPYILQTNIYADGFGNREERIYLWFDPTKDFHTYSVLWNLHQIVFMVDSIPIRVYRNHGDKGVPFPRRQPMSLEATLWNGDSWATRGGQDKIDWTKGPFIASFRNYNIDACVWKGNPRFCRVASHVNWWNLNNFSTLTSPQRRWFKWVRKYHMIYDYCQDNERFQNNLPQECSLPKY

>AhXTH58

MGGCHVCFLFLCLSSAMVVVSGSSSMNNNLPIIAFEDGYTPLFGDNNLIIHNDGKLVHLTLDQRTGSGFVSHELYLHGYFSAKIKLPADYTAGMSNGDMFKKNHDEIDFEFLGNIRGRDWRMQTNFYGNGSTNTGREERYDLWFDPSQDFHQYSILWTDSKIIEVKRTESMGGDFPSKPMTLYATIWDASDWATDGGKYRVNYKYAPYVAEFSNFVMHGCGVDPIDENVAMCNNAQNSKANIIPKHKIKMENFRNNHMTYSYCYDRARYQVPPPECVISLQEAEALRKLDPATFGDGRRHRGGGKRRHHQSKGRKAEDASF
