# Supplementary material for: Genome-wide identification of xyloglucan endotransglucosylase/hydrolase gene family members in peanut and their expression profiles during seed germination
Source: PeerJ. 2022 May 17;10:e13428. doi: 10.7717/peerj.13428 (PMC9121870; doi:10.7717/peerj.13428)
Supplement: Supplemental Information 5 — Amino acid sequences from 58 AhXTHs in peanut were aligned using PttXET16-34 (1UN1) as a referent sequence by MEGA X, and their secondary structures were predicted using ESPript. The conserved residues were shown in blue frames. [file peerj-10-13428-s005.pdf]

```
1UN1
                                                       1        10        20
                                                       .         .         .
1UN1         ...............................MAAAYPWTLFLGMLVMVSGTMGAALRKPV
PttXTH16-34  ...............................MAAAYPWTLFLGMLVMVSGTMGAALRKPV
XTH21        ............................MGSRRVGVLTLSLVIVASLVSAAMCGVPRRPV
XTH51        ............................MGSRRVGVLTLSLVVVASLVSAAMCGVPRRPV
XTH22        ..............................MGYYSMWSVCCVILGWFVSVGICGTPRRPM
XTH52        ..............................MGYYSLWSVCCVILAWFVSVGICSTPRRPM
XTH55        ...................................MSSLIFVFLIILYFIDGVLARR.SR
XTH56        ...................................MSSLIFVFLIVLFTDDGVLVRGGNS
XTH18        .................................MVRMEAKASSSLGVILLLVVIAEAAVS
XTH47        .................................MVRMEAKASSSLGVILLLVVIAEAAVS
XTH10        .................................MSKEVSLFLGLVMGFVFVGVAFAAATA
XTH39        .................................MSKEVSLFLGLVMGFVFVGVAFAAATA
XTH24        .....................................MSKMSSLLGFFVGLVLVGVVASS
XTH54        .....................................MSKMSSLLGFFVGLVLVGVVASS
XTH2         .........................MATFYPLIRNGGGSLFLILLSWVFLVSSLLCVLGR
XTH28        .........................MATFYPLIRNGGGSLFLILLSCVFLVSSLLCVLGR
XTH17        ...............................MYMAFFKNPFFLLLSLWALVLSGVCVWGK
XTH46        ...............................MYMAFFKNPFFLLLSLWALVLSGVCVWGK
XTH25        ..............................MFLDSQIALFFLIGIVSSILFHISVASVVS
XTH57        ...............................MNNFHIALFFLIGIVSSILFQISVASVVS
XTH19        ...................................MAAASEKMFLALLFIFFMARGIIIV
XTH48        ...................................MAAASEKMVLALLFIFFMARGIIIV
XTH40        .........................................MLVALFICVVVLVGNIVQV
XTH3         MHIIDHSYTSLGFFCFLLSLYTYLPPIFQPNQIKGTIINMGIEVLIINIVLLIGFAVAAS
XTH34        .......................................MGIEVLIINSVLLIGFAVAAS
XTH11        ..................................MTTLVTKVPIGFLSLLLIITIATKAA
XTH41        ..................................MTTLVTKVPIGFLSLLLIITIATKAA
XTH6         ........................................MAASTLSYLLLIPLLMVVAY
XTH29        ........................................MAASTLSYLLLIPLLMVVAY
XTH35        .....................................MASSSPSLLLIPLLVGSIMVVAY
XTH30        ....................................MASSKVVVLVVPLLVMSFCMVSCW
XTH4         ............................MAAACVNNNNDALIVIVITLFLIITLPSSSMA
XTH33        ............................MVASCVNNNNDALIVIVITLFLILPP..SSMA
XTH5         ....................................MLLPYPTSVVMVALFTMTMMMMLC
XTH32        ....................................MLLPYPTSVVMVALFSMTMMMMLC
XTH31        ...............................................MNPTSLPKKQFPD
XTH14        ..............................MGSTCTNHNGFYVGIMVIGLVVVSTMVGSC
XTH38        ..............................MGSTCTNHNGFYVGIMVIGLVVVSTMVGSC
XTH15        .................................MGGSTTKGFNLLVVGVVVVVTTMVGTC
XTH44        ......................................MGGFNLLVVGVVVVVTTMVGTC
XTH1         ....................................MAPSWGGSYFLFLCMLLGFSTIAY
XTH27        ....................................MAPSWGGSYFLLLCMLLGFSTIAY
```

```
                α1     β1      η1 β2  η2       β3          β4         β5
1UN1
             30        40        50        60        70        80
             .         .         .         .         .         .
1UN1         DVAFGRNYVPTWAFDHIKYFN.GGNEIQLHLDKYTGTGFQSKGSYLFGHFSMQMKLVPGD
PttXTH16-34  DVAFGRNYVPTWAFDHIKYFN.GGNEIQLHLDKYTGTGFQSKGSYLFGHFSMQMKLVPGD
XTH21        DVQFGRNYVPTWAFDHIKYFN.GGSEIQLHLDKYTGTGFQSKGSYLFGHFSMYIKMVPGD
XTH51        DVQFGRNYVPTWAFDHIKYFN.GGSEIQLHLDKYTGTGFQSKGSYLFGHFSMYIKMVPGD
XTH22        AVPFGRNYVPTWAYDHIKYLN.SGYDAQLLLDKYTGTGFQSKGSYLFGHFSMDIKMVAGD
XTH52        AVPFGRNYVPTWAYDHIKYLN.SGYDAQLLLDKYTGTGFQSKGSYLFGHFSMDIKMVAGD
XTH55        EVSFDQNYKVIWGDNHVVSLN.QGTEIQLLMDNSSGSGFGSKKNYGSGFFHLRIKVPGGD
XTH56        EINFDQNYKVIWGDNHAVSLN.QGTEIQLLMDNSSGAGIGSKMDYGSGFFHLRIKVPGND
XTH18        KGSFEDNFSIMWSEDHFSTSK.DGQIWYLSLDKDTGCGFQTKQRYRFGWFSMKLKLVAGD
XTH47        KGSFEDNFSIMWSEDHFSTSK.DGQIWYLSLDKDTGCGFQTKQRYRFGWFSMKLKLVAGD
XTH10        K..FEELFQPSWALDHFIH...EGDLLKLKLDNYSGAGFVSKSKYMFGKVTVQLKLVEGD
XTH39        K..FEELFQPSWALDHFIH...EGDLLKLKLDNYSGAGFVSKSKYMFGKVTVQLKLVEGD
XTH24        K..FEELYQPAWALDHFIH...DGELIKLKLDNYSGAGFGSKSKYMFGKVSIQLKLVEGD
XTH54        K..FEELYQPAWAFDHFIH...DGELIKLKLDNYSGAGFGSKSKYMFGKVSIQLKLVEGD
XTH2         PATFEQDFRATWSESHIRHTD.QGRTIQLMLDRSSGCGFASKVKYMFGRVSMKIKLVPGD
XTH28        PATFEQDFRATWSESHIRHID.QGRTIQLMLDQSSGCGFASKVKYMFGRVSMKIKLVPGD
XTH17        PVTFLQDFRVTWSDSHIRQIDNQGTAIQLILDRNSGCGFASKSRYMFGRISMKIKLIAGD
XTH46        PVTFLQDFRVTWSDSHIRQIDNQGTAIQLILDRNSGCGFASKSRYMFGRISMKIKLIAGD
XTH25        TGNFNKDFYVLWSPTHVNTSA.DGHTRTLKLDQQSGAGFASNQMFLFGQIDMQIKLVPGD
XTH57        TGNFNKDFYVLWSPTHVNTSA.DGHTRTLKLDQQSGAGFASNQMFLFGQIDMQIKLVPGD
XTH19        DANFGKSMYLTWGTQHASIQG...EDLQLVLHQTSGSAAQTKIPFLFGSIESKIKLVPNN
XTH48        DANFGKSMYLTWGTQHASIQG...EDLQLVLDQTSGSAAQTKIPFLFGSIESKIKLVPNN
XTH40        DGNFSKSMYLTWGVQHASIMG...EDLHLVLDTTSGSAAKSKRSFLFGSIEMLIKLIPGN
XTH3         AGNFNQDFEITWGNDRAKVLN.NGQLLTLSLDKASGSGFRSRNEYLFGKIDMQLKLVPGN
XTH34        AGNFNQDFEITWGNDRAKVLN.NGQLLTLSLDKASGSGFRSRNEYLFGKIDMQLKLVPGN
XTH11        AGNFYQDFEVTWGDNRAKIFN.GGQLLTLSLDRASGSGFRSKNEYLFAKLDMQIKLVPGN
XTH41        AGNFYQDFEVTWGDNRAKIFN.GGQLLTLSLDRASGSGFRSKNEYLFAKLDMQIKLVPGN
XTH6         AGNLDQEFDITWGDGRAKMLN.NGELLTLSLDKASGSGFQSKNEYLFGKIDMQLKLVPGN
XTH29        AGNLDQEFDITWGDGRAKMLN.NGELLTLSLDKASGSGFQSKNEYLFGKIDMQLKLVPGN
XTH35        GAKLDQEVDITWGDGRAKMLN.NGELLTLSLDKASGSGFQSKNEYLFGKIDMQLKLVSGN
XTH30        GGNFNKDFQITWGDGRAKILN.NGNLLTLSLDKASGSGFQSTNEYLFGKIDMQLKLVPGN
XTH4         GSNFNQQVDITWGDGRGKILN.SGKILTLSLDRASGSGFQSKNEYLYGKIDMQIKLVPGN
XTH33        GSNFNQQVDITWGDGRGKILN.SGKILTLSLDRASGSGFQSKNQYLYGKIDMQIKLVPGN
XTH5         GGDLHKNIDITWGNGRAQMLN.NGQLLTVSLDAASGSGFQSKDHYLFGHFQIQLKLVPGN
XTH32        GGDLHKKIDITWGNGRAQMLN.NGQLLTVSLDAASGSGFQSKDHYLFGHFQIQLKLVPGN
XTH31        SQLLDQEFDITWGDGRAKMLN.NGELLTLSLDKASCFGFQSKNEFLFDKIDMQLKLVSGN
XTH14        EGNFNQEFDLTWGGNRAKIFG.GGQLLSLSLDRVSGSGFQSKREYLFGRIDMQLKLVAGN
XTH38        EGNFNQEFDLTWGGNRAKIFG.GGQLLSLSLDRVSGSGFQSKREYLFGRIDMQLKLVAGN
XTH15        SANFYDDFDLTWGENRAKIFN.GGQLLSLSLDKVSGSGFRSKKEYMFGRIDMQLKLVSGN
XTH44        SANFYDDFDLTWGENRAKIFN.GGQLLSLSLDKVSGSGFRSKKEYMFGRIDMQLKLVSGN
XTH1         GGNFSTDFDLLFGDDRVKISD.GGQSMSLAMDKYSGSGVATKDQFLFGRFDMKIKLVGGN
XTH27        GGNFNTDFDLLFGDDRVKISD.GGQSMSLSMDKYSGSGVATKDQFLFGRFDMKIKLVGGN
```

```
1UN1          TT   β6              β7            TT     β8       TT       β9
              90        100         110          120          130          140
1UN1          SAGTVTAFYLSSQNS....EHDEIDFEFLGNRTGQPYILQTNVFTGGKGDREQRIYLWFD
PttXTH16-34   SAGTVTAFYLSSQNS....EHDEIDFEFLGNRTGQPYILQTNVFTGGKGDREQRIYLWFD
XTH21         SAGTVTAFYLSSQTA....EHDEIDFEFLGNRTGQPYILQTNVFTGGKENRES.......
XTH51         SAGTVTAFYLSSQTA....EHDEIDFEFLGNRTGQPYILQTNVFTGGKGDRE........
XTH22         SAGTVTAFYLSSLGT....EHDEIDFEFLGNRTGQPYILQTNVFTGGKGDREQRIYLWFD
XTH52         SAGTVTAFYLSSLGT....EHDEIDFEFLGNRTGQPYILQTNVFTGGKGDREQRIYLWFD
XTH55         SAGVVTAYYMTSQGS....RHDELDFEFLGNKKGKPYILQTNVFSEGEGNREQ.......
XTH56         SAGVVTAYYMSSQGS..SSRHDELDFELLGNREEKPYILQTNVFADDGGNREQKLKLWFD
XTH18         SAGVVTAYYMCSENGAG.PERDELDFEFLGNRTGQPFLIQTNVYKNGTGGREMRHMLWFD
XTH47         SAGVVTAYYMCSENGAG.PERDELDFEFLGNRTGQPFLIQTNVYKNGTGGREMRHMLWFD
XTH10         SAGTVTAFYMSSE...G.PNHNEFDFEFLGNTTGEPYSVQTNVYVNGVGNREQRLNLWFD
XTH39         SAGTVTAFYMSSE...G.PNHNEFDFEFLGNTTGEPYSVQTNVYVNGVGNREQRLNLWFD
XTH24         SAGTVTAFYMSSE...G.PNHNEFDFEFLGNTTGEPYSVQTNVYVNGVGNREQRLDLWFD
XTH54         SAGTVTAFYMSSE...G.PNHNEFDFEFLGNTTGEPYSVQTNVYVNGVGNREQRLDLWFD
XTH2          SAGTVTAFYMNSDTD...SVRDELDFEFLGNRSGQPYTVQTNVYAHGKGDREQRVNLWFD
XTH28         SAGTVTAFYMNSDTD...SVRDELDFEFLGNRSGQPYTVQTNVYAHGKGNREQRVNLWFD
XTH17         SAGTVTAFYMSSDTD...AIRDELDFEFLGNRSGQPYTVQTNIYAHGKGGREQRVNLWFD
XTH46         SAGTVTAFYMSSDTD...AIRDELDFEFLGNRSGQPYTVQTNIYAHGKGGREQRVNLWFD
XTH25         SAGTVLAYYMASDQP....NRDEIDFEFLGNMSEQPYILQTNIYADGFGNREERIYLWFD
XTH57         SAGTVLAYYMASDQP....NRDEIDFEFLGNMSEQPYILQTNIYADGFGNREERIYLWFD
XTH19         SAGTVTAYYLSSTGS....QHDEIDFEFLGNISGQPYIVHTNIYTQGNGSKEQQFYLWFD
XTH48         SAGTVTAYYLSSTGS....QHDEIDFEFLGNISGQPYIVHTNIYTQGNGSKEQQFYLWFD
XTH40         AAGIVTAYYLSSTGS....QHDEIDFEFLGNITGQPYTVNTNIYTQGKGNKEQQFYLWFD
XTH3          SAGTVTAYYLSSLGD....THDEIDFEFLGNLSGEPYTLHTNVFTQGKGNREQQFHLWFD
XTH34         SAGTVTAYYLSSLGD....THDEIDFEFLGNLSGEPYTLHTNVFTQGKGNREQQFHLWFD
XTH11         SAGTVTTYYLSSSGG....THDEIDFEFLGNLSGDPYILHTNVFTQGKGNREQQFYLWFD
XTH41         SAGTVTTYYLSSLGG....THDEIDFEFLGNLSGDPYILHTNVFTQGRGNREQQFYLWFD
XTH6          SAGTVTAYYLSSKGT....TWDEIDYEFLGNLSGDPYILHTNVFSQGKGNREQQFYLWFD
XTH29         SAGTVTAYYLSSKGT....TWDEIDYEFLGNLSGDPYILHTNVFSQGKGNREQQFYLWFD
XTH35         SAGTVTAYYLSSKGA....TWDEIDYEFLGNLSGDPYILHTNVFSQGKGNREQQFYLWFD
XTH30         SAGTVTAYYLSSKGA....TWDEIDFEFLGNLSGDPYILHTNVFSQGKGNREQQFYLWFD
XTH4          SAGTVTAYYLRSDGI....SWDEIDFEFLGNLSGDPYVVHTNVYTQGKGGKEQQFYLWFD
XTH33         SAGTVTAYYLRSDGI....SWDEIDFEFLGNLSGDPYVVHTNVYTQGTGGREQQFYLWFD
XTH5          SAGTVTSFYLQSEGS....TWDEIDFEFLGNLSGNPYILHSNVITQGKGGREQQFYLWFD
XTH32         SAGTVTSFYLQSEGS....TWDEIDFEFLGNLSGNPYILHTNVITQGKGGREQQFYLWFD
XTH31         SAGTVTAYYLSSKGS....NWDEIDFEFLGNVSGEPYILHTNVFSNGKGNREQQFYLRFD
XTH14         SAGTVTAYYLSSQGP....THDEIDFEFLGNLSGDPYTLHTNIFTQGKGDREQQFHLWFD
XTH38         SAGTVTAYYLSSQGP....THDEIDFEFLGNLSGDPYTLHTNIFTQGKGDREQQFHLWFD
XTH15         SAGTVTAYYLSSEGA....THDEIDFEFLGNVSGEPYILHTNVFSQGKGNREQQFYLWFD
XTH44         SAG.....TLSSEGA....THDEIDFEFLGNVSGEPYILHTNVFSQGKGNREQQFYLWFD
XTH1          SAGTVTAFYLSSTGD....HHDEVDLEFLGNLTGDPYMLSTNVFANGVGGREVQYYLWFD
XTH27         SAGTVTAFYLSSQGD....HHDEVDLEFLGNLTGDPYMLSTNVFANGVGGREVQYYLWFD
```

```
1UN1                 β10          β11    TT   β12         η3          β13
                     150          160          170          180          190          200
1UN1          PTKEFHYYSVLWNMYMIVFLVDDVPIRVFKNCKDLGVKFPFNQPMKIYSSLWNADDWATR
PttXTH16-34   PTKEFHYYSVLWNMYMIVFLVDDVPIRVFKNCKDLGVKFPFNQPMKIYSSLWNADDWATR
XTH21         ....ISGLIPPKNTTDIQFFVDDVPIRVFKNCKDLGVKFPFDQPMKIYNSLWNADDWATR
XTH51         ............QRIYLWFFVDDVPIRVFKNCKDLGVKFPFDQPMKIYNSLWNADDWATR
XTH22         PTKEYHRYSVLWNLYQIVFFVDNIPIRVFKNNKRMGVKFPFNQPMKIYNSLWNADDWATR
XTH52         PTKEYHRYSVLWNLYQIVFFVDNIPIRVFKNNKRMGVKFPFNQPMKIYNSLWNADDWATR
XTH55         .................RFFVDNIPIRVFKNMSNIGVRYPT.KPMQTQASLWDGDSWATD
XTH56         PRQDFHDYQILWNQHHIVFFVDNIPIRVFKNMSNIGVSYPT.KPMQIHASLWDGDSWATD
XTH18         PTEDYHTYSILWNNHQIVFFVDRVPIRVFKNNGKENNFFPNEKPMYLFSSIWNADEWATR
XTH47         PTEDYHTYSILWNNHQIVFFVDRVPIRVFKNNGKENNFFPNEKPMYLFSSIWNADEWATR
XTH10         PTKDFHSYSFFWNQRQVIFLVDETPIRVHTNMEHRGIPFPKDQAMGVYSSIWNADDWATQ
XTH39         PTKDFHSYSFFWNQRQVIFLVDETPIRVHTNMEHRGIPFPKDQAMGVYSSIWNADDWATQ
XTH24         PTKDFHTYSIFWNQRQVVFLVDDTPIRVHTNLEHKGIPFPKDQAMGVYSSIWNADDWATQ
XTH54         PTKDFHTYSIFWNQRQVVFLVDETPIRVHTNLEHKGIPFPKDQAMGVYSSIWNADDWATQ
XTH2          PSADFHTYTILWNHKHIVFSVDDFPIRVYKNNEAKGVPYPKMQAMGVYSTLWEADNWATR
XTH28         PSADFHTYTILWNHKHIVFSVDDFPIRVYKNNEAKGVPYPKMQAMGVYSTLWEADNWATR
XTH17         PSAQFHTYTILWNHHHIVFYVDEFPIRVYKNDVARGVAYPRMQGMGVYSTLWEADNWATR
XTH46         PSAQFHTYTILWNHHHIVFYVDEFPIRVYKNDVARGVAYPRMQGMGVYSTLWEADNWATR
XTH25         PTKDFHTYSVLWNLHQIVFMVDSIPIRVYRNHGDKGVPFPRRQPMSLEATLWNGDSWATR
XTH57         PTKDFHTYSVLWNLHQIVFMVDSIPIRVYRNHGDKGVPFPRRQPMSLEATLWNGDSWATR
XTH19         PTADFHNYTIHWNPTQVVWYIDSIPIRVFMNNEEEGIAYPNKQGMKVYTSLWNADDWATR
XTH48         PTADFHNYTIHWNPTQVVWYIDSIPIRVFMNYEEEGIAYPNKQGMKVYTSLWNADDWATR
XTH40         PAADFHNYTIHWNPTQIVWYVDGLPIRVFQNYENHGVAYPNKHGMRVYSSLWNADDWATR
XTH3          PTKDFHTYSLLWNPQSIIFSVDGTPIREFKNWESKGVPFPKNQAMRIYSSLWDAEDWATR
XTH34         PTKDFHTYSLLWNPQSIIFSVDGTPIREFKNWESKGVPFPKNQAMRIYSSLWDAEDWATR
XTH11         PTADFHTYSILWNPHNIIFSVDGTPIREFKNLESRGVPFPKNQPMRLYSSLWNADDWATR
XTH41         PTADFHTYSILWNPHNIIFSVDGTPIREFKNLESRGVPFPKNQPMRLYSSLWNADDWATR
XTH6          PTADFHTYSITWNPQRIIFSVDGTPIREFKNSESMGVPFPKSQPMRIYSSLWNADDWATR
XTH29         PTADFHTYSITWNPQRIIFSVDGTPIREFKNSESMGVPFPKSQPMRIYSSLWNADDWATR
XTH35         PTADFHTYSITWNPQRIIFSVDGTPIREFKNSESIGVPFPKSQPMRIYSSLWNADDWATR
XTH30         PTADFHTYSILWNPQRIVFSVDGTPIREFKNLESAGVPFPKNQPMRIYSSLWNADDWATR
XTH4          PTANLHTYSILWNPAHIVFYIDGRPIREFKNLEGVGVSYPKNQPMKLYSSIWNADDWATR
XTH33         PTANLHTYSILWNPAHIVFYIDGRAIREFKNLEGVGIPYPKNQPMKLYSSLWNADDWATR
XTH5          PTSDFHTYSILWNPLCIILYVDGIPIREFKNYESRNISFPMKKPMRMYGSLWDAEDWATR
XTH32         PTSDFHTYSILWNPLCVILYVDGIPIREFKNYESRNISFPMKKPMRMYGSLWDAEDWATK
XTH31         PTTDFHTYSILWNPQLIMFSVDSSPIRQ................................
XTH14         PTKNFHTYSVIWKPQHIIFLVDNIPIRVFKNAESMGVPFPKKQPMRIYSSLWNADDWATR
XTH38         PTKNFHTYSVIWKPQHIIFLVDNIPIRVFKNAESMGVPFPKKQPMRIYSSLWNADDWATR
XTH15         PTTNFHTYSIIWNPHHITFLVDNIPIRVFKNAESDGVPFPKNQPMRIYSSLWNADDWATR
XTH44         PTTNFHTYSIIWKPHHITFLVDNIPIRVFKNAESVGVPFPKNQPMRIYSSLWNADDWATR
XTH1          PTADYHTYSIDWSPTRIILWVDDTPIRVINNKEIIGVPFPTKQPMRLYTTLWNGDAWATR
XTH27         PTADYHTYSIDWSPTRIIIWVDDTPIRVINNKEIIGVPFPTKQPMRLYTTLWNGDAWATR
```

```
             η4      η5          β14                                  η6   η7
1UN1         ϱϱ     ϱϱϱ     ──────────────►   TTT                    ϱϱϱ  ϱϱϱ
                  210         220         230                 240        250
1UN1         GGLEKTDWSKAPFIASYRSFHIDGCEASVEA..........KFCATQG..ARWWDQKEFQ
PttXTH16-34  GGLEKTDWSKAPFIASYRSFHIDGCEASVEA..........KFCATQG..ARWWDQKEFQ
XTH21        GGLEKTDWSKAPFIASYKGFHIDGCEASVEA..........KFCSTQG..KRWWDQQEFR
XTH51        GGLEKTDWSKAPFIASYKGFHIDGCEASVEA..........KFCSTQG..KRWWDQQEFR
XTH22        GGLEKTDWSKAPFIASYKGFHIDGCETSVEA..........KFCSTQG..KRWWDQQEFR
XTH52        GGLEKTDWSKAPFIASYKGFHIDGCETSVEA..........KFCSTQG..KRWWDQQEFR
XTH55        GGRTKINWNSAPFKAYFQGFDVKGCEVLQDS..........SDIQHCASHKYQWNTPSFW
XTH56        GGRTKINWSSAPFKAYFQGFDVKGCEVLQNS..........SDIQHCDSDKYQWNTPSFW
XTH18        GGLEKTNWKLAPFVSSYKDFSVDGCQWKDP...........YPACVSTTTDNWWDQYSAW
XTH47        GGLEKTNWKLAPFVSSYKDFSVDGCQWKDP...........YPACVSTTTDNWWDQYSAW
XTH10        GGRVKTNWSHAPFIATYKAFEINGCECPIVSSTS...VENLKRCSSNE.KKYWWDEPNLG
XTH39        GGRVKTNWSHAPFIATYKAFEINACECPIVSSKS...VENLKRCSSNE.KKYWWDEPNLG
XTH24        GGRVKTDWSHAPFVATYKDFTIDACECPVGVSSSSVAPENAKRCSSSEDKKYWWDEPTMS
XTH54        GGRVKTDWSHAPFVATYKDFTIDACECPVGVSSSSVAPENAKRCSSSEDKKYWWDEPTMS
XTH2         GGLEKIDWSKAPFNAYYKDFDIEGCSVPGPA.............SCASNPSNWWEGAAYQ
XTH28        GGLEKIDWSKAPFNAYYKDFDIEGCSVPGPA.............SCSSNPSNWWEGAAYQ
XTH17        GGLEKIDWRKAPFYAYYKDFDIEGCQLPGPT.............SCASNASNWWEGAAYQ
XTH46        GGLEKIDWRKAPFYAYYKDFDIEGCQLPGPT.............SCASNASNWWEGAAYQ
XTH25        GGQDKIDWTKGPFIASFRNYNIDACVWKGNP...........RFCRVASHVNWWNLNNFS
XTH57        GGQDKIDWTKGPFIASFRNYNIDACVWKGNP...........RFCRVASHVNWWNLNNFS
XTH19        GGLVKTNWTNAPFIARLNRFRARACKWNGPISIN.........NCASNVPSNWWTSPIYK
XTH48        GGLVKTNWTNAPFIARLNRFRARACKWNGPISIN.........NCASNVPSNWWTSPIYK
XTH40        GGLVKTDWRGAPFIASFHHFRARACKWNGAVSIN.........HCASNVPANWWISPLYK
XTH3         GGLVKTDWTQAPFTASYKGFNAQACVWTSSSGSSCSSKQ..............GQSWFTQ
XTH34        GGLVKTDWTQAPFTASYKGFNAQACVWSSSSGSSCSSKQ..............GQSWFTQ
XTH11        GGLVKTDWAHAPFTASYRNFNAPP.............................AQSWMGQ
XTH41        GGLVKTDWAHAPFTASYRNFNAPP.............................AQSWMGQ
XTH6         GGLVKTDWTKAPFTASYRNFNAD.ACIWSNGASSCGSGSG...SSS......TSSSWLSQ
XTH29        GGLVKTDWTNAPFTASYRNFNAD.ACIWSNGASSCGSGSG...SSS......TSSSWLSQ
XTH35        GGLVKTDWTKAPFTASYRNFNAD.ACIWSNGASSCGSGSG...LGSSSTT..SSSSWLSQ
XTH30        GGLVKTDWSKAPFTASYRNFNANNACIWKNGRSSCKS................SSSWLSQ
XTH4         GGLVKTDWSQAPFTASFKNLKANGCVWSNGVSSCNLSSSN...SSNN......NNSWLSQ
XTH33        GGLVKTDWSQAPFTASFRNLKANGCVWSNGVSSCNLSS.N...SSDN......NNSWLSQ
XTH5         SGLIKTNWSAAPFIAYFKNFFVNACVESTPPSITSSCTHP...NKKSNNHNSVGEKWITQ
XTH32        GGVIKTNWSAAPFIAYFKNFFVNACVESTPPSIKSSCTHL...NKKNNNHNSVGEKWITQ
XTH31        ............................................................
XTH14        GGLVKTDWAKAPFTAYYRNFRATQLSSASLRPNTRSSEWE...TS...............
XTH38        GGLVKTDWAKAPFTAYYRNFRATQLSSASLRPNTRSSEWE...TS...............
XTH15        GGLVKTDWSKAPFTAYYRNFKVTGFSATSAFSDVATSEIQ...NSGK...........DS
XTH44        GGLVKTDWSKAPFTAYYRNFKATAFSATSAFSNVVTSEIQ...GSGE...........DS
XTH1         WGQVKIDLSQAPFIARFKNYNGTACVPKKGIADCKG.................FGASMKR
XTH27        WGQVKIDLSQAPFIARFKNYNGTACVPKKGIADCKG.................FGASMKK


                          α2             β15  η8            α3
1UN1            ϱϱϱϱϱϱϱϱϱϱϱϱϱϱ     ────► ϱϱϱ              ϱϱϱϱ
                  260         270         280         290
1UN1         DLDAFQYRRLSWVRQKYTIYNYCTDRSRYPSMPPECKRDRDI..................
PttXTH16-34  DLDAFQYRRLSWVRQKYTIYNYCTDRSRYPSMPPECKRDRDI..................
XTH21        DLDALQWRRLRWVRQKFTIYNYCNDRKRYPTLPPECSRDRDI..................
XTH51        DLDALQWRRLRWVRQKFTIYNYCNDRKRYPTLPPECSRDRDI..................
XTH22        DLDSYQWRRLRWVRRRFTIYNYCSDRTRYPQMPPECRRNGDY..................
XTH52        DLDSYQWRRLRWVRRRFTIYNYCSDRTRYPQMPPECRTNGDY..................
XTH55        QLDPVRQRQYENVKTRYMIYDYCTDRKRNPTPPLECQN......................
XTH56        QLDPVRQRQYEDVKTRYMIYDYCTDRKRNPTPPLECQH......................
XTH18        HLSDDQKKDYAWVRRNLVIYDYCNDSERYPTLPEECSLSPWD..................
XTH47        HLSDDQKKDYAWVQRNLVIYDYCNDSQRYPILPEECSLSPWD..................
XTH10        VLSLHQSHQLMWVRAKHMVYDYCADTARFPVMPAECVHHSHHKLVLKN............
XTH39        VLSLHQSHQLMWVRAKHMVYDYCADTARFPVMPAECVHHSHHKLVLKN............
XTH24        ELNVHQSHQLMWVRANHMVYDYCTDTARFPVTPAECVHHRH...................
XTH54        ELNVHQSHQLMWVRANHMVYDYCTDTARFPVTPAECVHHRH...................
XTH2         ELSAIQARRYRWVRINHVVYDYCQDKSRFPHFALMFVGKKPNMEEHKRI...........
XTH28        ELSAIQARRYRWVRINHVVYDYCQDKSRFPVTPPECLAGI....................
XTH17        ALTPTQARLYRWVRINHIIYDYCQDKPRFPLGPPPECLS.....................
XTH46        ALTPTQARLYRWVRINHIIYDYCQDKPRFPLGPPPECLS.....................
XTH25        TLTSPQRRWFKWVRKYHMIYDYCQDNERFQNNLPQEWSLPKY..................
XTH57        TLTSPQRRWFKWVRKYHMIYDYCQDNERFQNNLPQECSLPKY..................
XTH19        QLSYAQMGQLYWVRNNYMIYDYCKDTKRFNGQSSDFVI......................
XTH48        QLSYAQMGQLNWVRNNYMIYDYCKDTKRFNGQVPPECFKTQF..................
XTH40        QLSYSEKGQLNWVRKNYMIYDYCADSKRFNGQLPPECSKTQL..................
XTH3         SLDSTGQARIQWVQKNYMIYNYCTDTKRFPQGLPPECTLA....................
XTH34        SLDSTGQARIQWVQKNYMIYNYCTDTKRFPQGLPPECTLA....................
XTH11        SLDSTGLARIHWVQRNYMIYNYCTDLKRFPQGPPLECSLA....................
XTH41        SLDSTGLARIHWVQKNYMIYNYCTDLKRFPQGPPPECSLA....................
XTH6         ELDTTAQERLRWVQKNYMIYNYCTDAKRFPQGFPPECRTS....................
XTH29        ELDTTAQERLRWVQKNYMIYNYCTDAKRFPQGFPPECRTS....................
XTH35        ELDTTAQERLRWVQKNYMIYNYCTDAKRFPQGFPPECRTS....................
XTH30        ELDSTGLQRLRWVQKNYMIYNYCTDKKRFPRGFPIECNRS....................
XTH4         QLDSNGQRKLKWVQKNYMIYNYCSDINRFPQGLPLECTLRTT..................
XTH33        QLDSNGQRKLKWVQKNYMIYNYCSDINRFPQGLPLECTLLTT..................
XTH5         GLKPSEVDKLNWVHKNFIVYNYCSDLKRFPQGLPLECIPSVQKEKLLDQEFDITWGDGRA
XTH32        ELKPSEVDKLNWVHKNFMVYNYCSDLKRFPQGLPLECKIN....................
XTH31        ............................................................
XTH14        DIDAIGRRRLRWVQKYFMIYNYCNDFKRFPQGLPAECRSRF...................
XTH38        DIDAVGRRRLRWVQKYFMIYNYCNDFKRFPQGLPAECRSRF...................
XTH15        VLDAYGRRRLRWVQKYFMVYNYCNDLKRFPEGIPAECSHGRF..................
XTH44        VLDAYGRRRLRWVQKYFMVYNYCNDPKRFPQGIPAECSHGRF..................
XTH1         GLDNENKKKLKQVNSKWVVYHYCRDLRRYAHGLPFECRRDNMAHSDNNQ...........
XTH27        GIDNENKKKLKQVNSKWVVYHYCRDLRRYAHGLPFECRRDNMAHSDNNQ...........
                                     1           1
```

1UN1

```
1UN1            ............................................................
PttXTH16-34     ............................................................
XTH21           ............................................................
XTH51           ............................................................
XTH22           ............................................................
XTH52           ............................................................
XTH55           ............................................................
XTH56           ............................................................
XTH18           ............................................................
XTH47           ............................................................
XTH10           ............................................................
XTH39           ............................................................
XTH24           ............................................................
XTH54           ............................................................
XTH2            ............................................................
XTH28           ............................................................
XTH17           ............................................................
XTH46           ............................................................
XTH25           ............................................................
XTH57           ............................................................
XTH19           ............................................................
XTH48           ............................................................
XTH40           ............................................................
XTH3            ............................................................
XTH34           ............................................................
XTH11           ............................................................
XTH41           ............................................................
XTH6            ............................................................
XTH29           ............................................................
XTH35           ............................................................
XTH30           ............................................................
XTH4            ............................................................
XTH33           ............................................................
XTH5            KMLNNGELLTLSLDKASCSGFQSKNEFLFGKIDMQLKLVPGNSLSSKGSNWDEIDFEFLG
XTH32           ............................................................
XTH31           ............................................................
XTH14           ............................................................
XTH38           ............................................................
XTH15           ............................................................
XTH44           ............................................................
XTH1            ............................................................
XTH27           ............................................................
```

1UN1

```
1UN1            ............................................................
PttXTH16-34     ............................................................
XTH21           ............................................................
XTH51           ............................................................
XTH22           ............................................................
XTH52           ............................................................
XTH55           ............................................................
XTH56           ............................................................
XTH18           ............................................................
XTH47           ............................................................
XTH10           ............................................................
XTH39           ............................................................
XTH24           ............................................................
XTH54           ............................................................
XTH2            ............................................................
XTH28           ............................................................
XTH17           ............................................................
XTH46           ............................................................
XTH25           ............................................................
XTH57           ............................................................
XTH19           ............................................................
XTH48           ............................................................
XTH40           ............................................................
XTH3            ............................................................
XTH34           ............................................................
XTH11           ............................................................
XTH41           ............................................................
XTH6            ............................................................
XTH29           ............................................................
XTH35           ............................................................
XTH30           ............................................................
XTH4            ............................................................
XTH33           ............................................................
XTH5            NVSGEPYILHTNVFSNGKGNTEQQFYLWFDPSADFHTYSILWNPQRIMFSVDSSPIREFK
XTH32           ............................................................
XTH31           ............................................................
XTH14           ............................................................
XTH38           ............................................................
XTH15           ............................................................
XTH44           ............................................................
XTH1            ............................................................
XTH27           ............................................................
```

```
1UN1

1UN1          ..............
PttXTH16-34   ..............
XTH21         ..............
XTH51         ..............
XTH22         ..............
XTH52         ..............
XTH55         ..............
XTH56         ..............
XTH18         ..............
XTH47         ..............
XTH10         ..............
XTH39         ..............
XTH24         ..............
XTH54         ..............
XTH2          ..............
XTH28         ..............
XTH17         ..............
XTH46         ..............
XTH25         ..............
XTH57         ..............
XTH19         ..............
XTH48         ..............
XTH40         ..............
XTH3          ..............
XTH34         ..............
XTH11         ..............
XTH41         ..............
XTH6          ..............
XTH29         ..............
XTH35         ..............
XTH30         ..............
XTH4          ..............
XTH33         ..............
XTH5          NMNQTEFHSQSRSQ
XTH32         ..............
XTH31         ..............
XTH14         ..............
XTH38         ..............
XTH15         ..............
XTH44         ..............
XTH1          ..............
XTH27         ..............
```

Supplementary Files 5 The multiple alignment of amino acid sequences among 58 AhXTHs in peanut and 1UN1

Amino acid sequences from 58 AhXTHs in peanut were aligned using PttXET16-34 (1UN1) as a referent sequence by MEGA X, and their secondary structures were predicted using ESPript. The conserved residues were shown in blue frames.
