# Supplementary material for: Genome-wide identification of xyloglucan endotransglucosylase/hydrolase gene family members in peanut and their expression profiles during seed germination
Source: PeerJ. 2022 May 17;10:e13428. doi: 10.7717/peerj.13428 (PMC9121870; doi:10.7717/peerj.13428)
Supplement: Supplemental Information 6 — Amino acid sequences from 58 AhXTHs in peanut were aligned using TmNXG1(2UWA) as a referent sequence by MEGA X, and their secondary structures were predicted using ESPript. The conserved residues were shown in blue frames. [file peerj-10-13428-s006.pdf]

```
                 η1        α1   β1
2UWA
           1        10        20        30        40        50
2UWA     .........MPPNILSIFLHLLPILMFSSSCLGQGPPSPGYYPSSQITSLGFDQGYTNLW
TmNXG1   .........MPPNILSIFLHLLPILMFSSSCLGQGPPSPGYYPSSQITSLGFDQGYTNLW
XTH8     .......MMKALLLFLFAISSSLFMEPCYSSSGYWPPSPGYWPSHKFRSMNFYKGFRNLW
XTH9     .MFTTMLPSSPLSFFFFFFLLVLSFMICASAQ..GPPSPGYYPGSKISPISFDQGFRNLW
XTH20    ...............MALFVFAILMLMAPSSNAEWPPSPGYWPSSKFKTMNFYKGFRNLW
XTH23    ..MPSLLSSSLLQMPPLLFSLILSLMLCGIIADESPPSPGYYPSSQVSSVAFDQAYRNLW
XTH37    .......MMKALLLFLFAISSSLFMEPCYSSSGYWPPSPGYWPSHKFRSMNFYKGFRNLW
XTH49    ......MMMKALLLFLFAISSSLFMEPCYSSSGYWPPSPGYWPSHKFRSMNFYKGFRNLW
XTH50    ........................MLMAPSSNAEWPPSPGYWPSSKFKTMNFYK......
XTH53    ..MPSLLSSSLLQMLPLLFSLIISLMLCGIIADESPPSPGYYPSSQVSSVAFDQAYRNLW
XTH7     ...............................MADPVLHPDTTNPLHHHQTQPLKEIAIDY
XTH12    ....MALIWQLKLLFSPLLIIILLAN.VVSSRTRPFTAPTVTPLTNSFPRVPIDPNFSNAF
XTH13    ...................MGLGGGLVTFFLCLLFFAAPSASSTNLLPIIPFDEGYAPLF
XTH16    MDHNNTLRRFGEIIPKTHRNHNFLLSLFLFFFFFFFFFFSSHAAFDLATIPFNDGYSPLF
XTH26    .................MGGCHLCFLFLCLSSAMVVVSGSSSMNNNLPIIAFEDGYTPLF
XTH36    ...............................MADPVLHPDTTNPLHHHQTQPLKEIAIDY
XTH42    ....MALIWQLNLLFSPLIIICLLSNVVSSRTRPFTAPTVTPLTNSFPRVPIDPAFSNAF
XTH43    ...................MGLGGGLVTFFLCLLFFAAPSASSTNLLPIIPFDEGYAPLF
XTH45    MDHNNTLRRFGEIIPKTHRNHNFLLSLFLFFFFFFFFFFSSHAAFDLATIPFNDGYSPLF
XTH58    .................MGGCHVCFLFLCLSSAMVVVSGSSSMNNNLPIIAFEDGYTPLF

          η2  β2      β3              β4                  β5
2UWA             T.T                                   TT
                  60        70        80        90        100
2UWA     GPQHQRVDQ.G.SLTIWLDSTSGSGFKSINRYRSGYFGANIKLQSGYTAGVITSFYLSNN
TmNXG1   GPQHQRVDQ.G.SLTIWLDSTSGSGFKSINRYRSGYFGANIKLQSGYTAGVITSFYLSNN
XTH8     GPQHQALDNNN.ALTIWLDRTSGSGFKSVRPFRSGYFGASIKLHPGYTAGVITAFYLSNN
XTH9     GPQHQRLEQG..TLSIWLDSNSGSGFKSLHSYQSGYFSAAIKLQPGYTAGVITTLYLSNN
XTH20    GPQHQRIEQN..ALTIWLDRTSGSGFKSVAPFRSGYFGASIKLQPGYTAGVITAFYLSNN
XTH23    GPQHQRLDQSG.SLTIWLDSYSGSGFKSIRPYRSGYFGAAIKLQSGYTAG......LSNN
XTH37    GPQHQALDNNN.ALTIWLDRTSGSGFKSVRPFRSGYFGASIKLHPGYTAGVITAFYLSNN
XTH49    GPQHQALDNNN.ALTIWLDRTSGSGFKSVRPFRSGYFGASIKLHPGYTAGVITAFYLSNN
XTH50    ....................GSGFKSVAPFRSGYFGASIKLQPGYTAGVITAFYLSNN
XTH53    GPQHQRLDQSG.SLTIWLDSYSGSGFKSIRPYRSGYFGAAIKLQSGYTAG......LSNN
XTH7     TPEACSHCPNSNTITLTFDHRGGARWRTTTRFHYGTFSSLIQCPKGNTNGLNFNLYLSSL
XTH12    GASNIKLLANGTMATLALDKLSGSGLVSKSSYYYGFFSAAIKLPAGISSGVVVAFYLSNA
XTH13    GDNNLVIHRDGKTVHLSLDERTGSGFVSHDLYLHGYFRASIKLPADYTAGVVVAFYMSNG
XTH16    GDSNVVRSDDGNGVNLLLDRFTGSGFISSSMYKYGFFSANIKLPSNYTAGICVAFYTSNG
XTH26    GDNNLIIHNDGKLVHLTLDQGTGSGFVSHELYLHGYFSAKIKLPADYTAGVVVAFYMSNG
XTH36    TPEACSHCPNSNTITLTFDHRGGARWRTTTRFHYGTFSSLIQCPKGNTNGLNFNLYLSSL
XTH42    GASNVKLLANGTMATLALDKLSGSGLVSKSSYYYGFFSAAIKLPAGISSGVVVAFYLSNA
XTH43    GDNNLVIHRDGKTVHLSLDERTGSGFVSHDLYLHGYFSASIKLPADYTAGVVVAFYMSNG
XTH45    GDSNVVRSDDGNGVNLLLDRFTGSGFISSSMYKYGFFSANIKLPSNYTAGICVAFYTSNG
XTH58    GDNNLIIHNDGKLVHLTLDQRTGSGFVSHELYLHGYFSAKIKLPADYTAGVVVAFYMSNG

                  β6             β7                β8
2UWA                        TT                          TT
          110       120       130       140       150       160
2UWA     QDYPGKHDEIDIEFLGTIPGKPYTLQTNVFIEGSGDYN.IIGREMRIHLWFDPTQDYHNY
TmNXG1   QDYPGKHDEIDIEFLGTIPGKPYTLQTNVFIEGSGDYN.IIGREMRIHLWFDPTQDYHNY
XTH8     EAHPGFHDEVDIEFLGTTFGKPYTLQTNVYIRGSGDG.TIIGREMKFHLWFDPTKDFHHY
XTH9     QDHPGNHDEIDIEFLGTTPDKPYVLQTNVYIRGTGD.GNIVGREMRFHLWFDPTQDFHIY
XTH20    EAHPGFHDEVDIEFLGTTFGKPYTLQTNVYIRGSGDG.QIIGREMKFHLWFDPTKDFHHY
XTH23    QDYPGDHDEVDIEFLGTIPDKQYVLQTNVFMRGSGDKNNVIGREMRFHLWFDPTQDFHHY
XTH37    EAHPGFHDEVDIEFLGTTFGKPYTLQTNVYIRGSGDG.TIIGREMKFHLWFDPTKDFHHY
XTH49    EAHPGFHDEVDIEFLGTTFGKPYTLQTNVYIRGSGDG.TIIGREMKFHLWFDPTKDFHHY
XTH50    EAHPGFHDEVDIEFLGTTFGKPYTLQTNVYIRGSGDG.QIIGREMKFHLWFDPTKDFHHY
XTH53    QDYPGDHDEVDIEFLGTIPGKQYVLQTNVFMRGSGDKNNVIGREMRFHLWFDPTQDFHHY
XTH7     E.GEKSQDEIDFEFLGKDR...TIVQTNYFSGGNGNK......EKIHHLGFDASDGFHEY
XTH12    DKFPHNHDEIDIELLGHDKRNDWVIQTNVYANGSVST....GREEKFYFWFDPTKQYHYY
XTH13    DMFQNNHDEIDFEFLGNIRGKDWRIQTNVYGNGSTSI....GREERYGLWFDPAEDFHQY
XTH16    DVFEKTHDELDFEFLGNLAGKPWRFQTNLYGNGSTHR....GREERYRLWFDPTKEYHRY
XTH26    DMFKKNHDEIDFEFLGNIRGRDWRMQTNFYGNGSTNT....GREERYDLWFDPSQDFHQY
XTH36    E.GEKSQDEIDFEFLGKDR...TIVQTNYFSGGNGNK......EKIHHLGFDASDGFHEY
XTH42    DKFPHNHDEIDIELLGHDKRNDWVIQTNVYANGSVST....GREEKFYFWFDPTKQYHYY
XTH43    DMFQNNHDEIDFEFLGNIRGKDWRIQTNVYGNGSTSI....GREERYGLWFDPAEDFHQY
XTH45    DVFEKTHDELDFEFLGNLAGKPWRFQTNLYGNGSTHR....GREERYRLWFDPTKEYHRY
XTH58    DMFKKNHDEIDFEFLGNIRGRDWRMQTNFYGNGSTNT....GREERYDLWFDPSQDFHQY

          β9     β10     β11       η3          β12          η4    η5
2UWA                   TT
           170       180       190       200       210       220
2UWA     AIYWTPSEIIFFVDDVPIRRYPRKSDA..TFPLRPLWVYGSVWDASSWATENGKYKADYR
TmNXG1   AIYWTPSEIIFFVDDVPIRRYPRKSDA..TFPLRPLWVYGSVWDASSWATENGKYKADYR
XTH8     AILWSPK.....................EIIPMWVYGSIWDASSWATEDGKYKADYR
XTH9     AILWKPSETIFFVDDVPIRRYPRKGDA..TYPNRPMYVYGSIWDASSWATEDGKYKANYK
XTH20    AILWSPKEIIFFVDDVPIRRYPRKSAE..TFPMRPMWLYGSIWDASSWATEDGKYKADYR
XTH23    AILWTPTDIIFLVDDVPIRNYPRKNDA..TFPERAMYVYGSIWDASSWATENGKYKADYK
XTH37    AILWSPK.....................EIIPMWVYGSIWDASSWATEDGKYKADYR
XTH49    AILWSPK.....................EIIPMWVYGSIWDASSWATEDGKYKADYR
XTH50    AILWTPKEIIFFVDDVPIRRYPRKSAE..TFPMRPMWLYGSIWDASSWATEDGKYKADYR
XTH53    AILWTPTDIIFLVDDVPIRNYPRKNDA..TFPERAMYVYGSIWDASSWATENGKYKADYK
XTH7     VIKWSYDVIEWLIDGKVVRREEKKEGKG..FPQKPMFLYASIWDASCIDNGRWAGKYDGS
XTH12    SILWNSYHTVFLVDNIPVREFIH....GSVFPSKPMSVYATIWDGSEWATHGGKYPVDYK
XTH13    SILWTDSQIIFYVDNVPIREIKRTESMGGDFPSKPMTLYATIWDASDWATNGGKYRVNYK
XTH16    SILWTAKNVVFYIDEVPIREVLRSAEMGADYPSKPMSLYATIWDASNWATSGGKYKVNYK
XTH26    SILWTDSKIIFYIDNVPIREVKRTESMGGDFPSKPMTLYATIWDASDWATDGGKYRVNYK
XTH36    VIKWSCDVIEWLIDGKVVRREEKKEGKG..FPQKPMFLYASIWDASCIANGMWAGKYDGS
XTH42    SILWNSYHTVFLVDNIPVREFIH....GTVFPSKPMSVYATIWDGSEWATHGGKYPVDYK
XTH43    SILWTDSQIIFYVDNVPIREIKRTESMGGDFPSKPMTLYATIWDASDWATNGGKYRVNYK
XTH45    SILWTAKNVVFYIDEVPIREVLRSAEMGADYPSKPMSLYATIWDASNWATSGGKYKVNYK
XTH58    SILWTDS.........KIIEVKRTESMGGDFPSKPMTLYATIWDASDWATDGGKYRVNYK
```

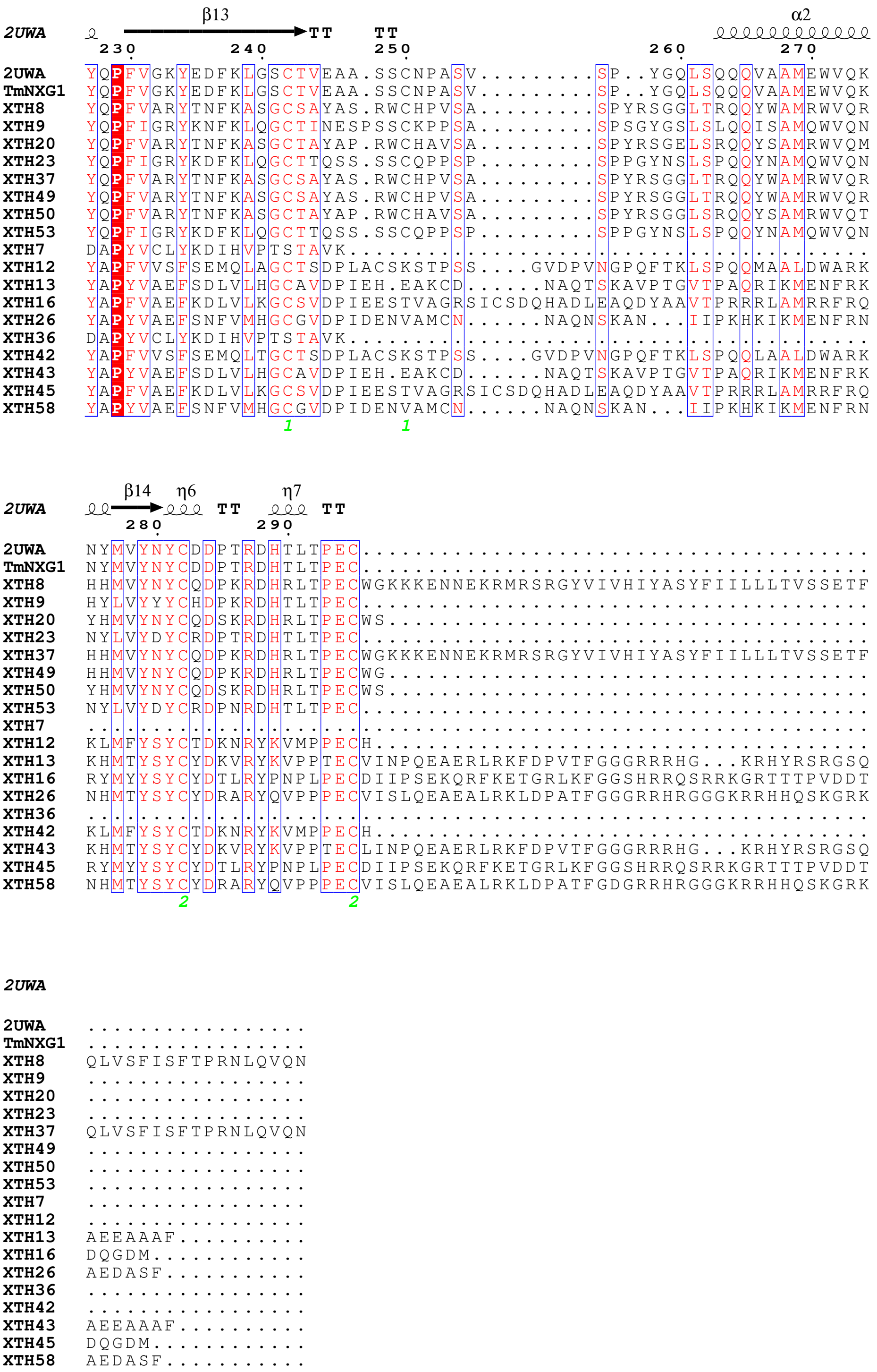

Supplementary Files 6 The multiple alignment of amino acid sequences among 58 AhXTHs in peanut and 2UWA

Amino acid sequences from 58 AhXTHs in peanut were aligned using TmNXG1(2UWA) as a referent sequence by MEGA X, and their secondary structures were predicted using ESPript. The conserved residues were shown in blue frames.
