# Supplementary figures and images for: Genome-wide identification of xyloglucan endotransglucosylase/hydrolase gene family members in peanut and their expression profiles during seed germination
Source: PeerJ. 2022 May 17;10:e13428. doi: 10.7717/peerj.13428 (PMC9121870; doi:10.7717/peerj.13428)

Pearson correlation between samples

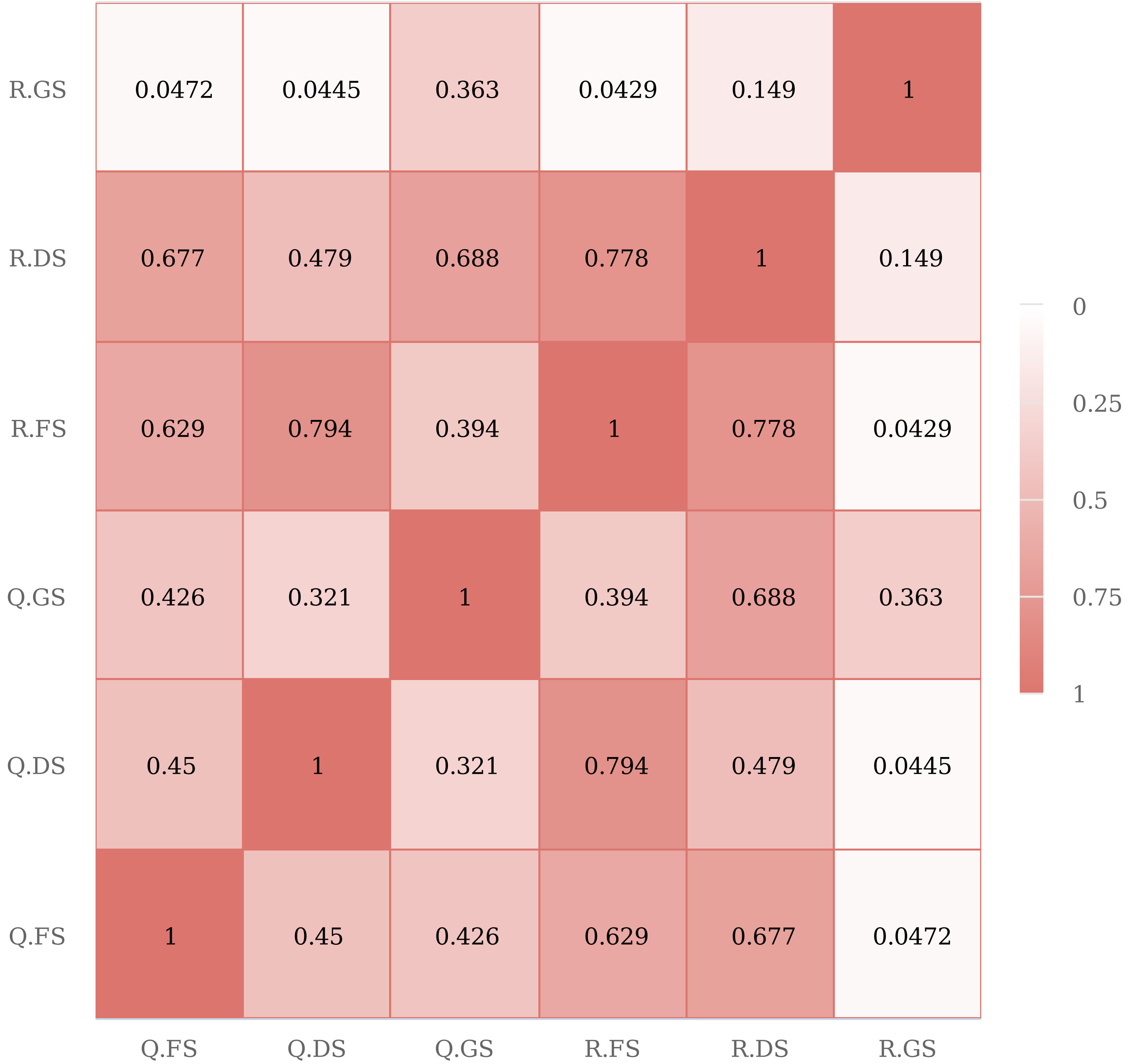

Supplement: Supplemental Information 8 — Q.FS, Q.DS, and Q.GS respectively denote the qRT-PCR results in the freshly harvested seeds (FS), the dried seeds (DS) and the newly germinated seeds (GS). R.FS, R.DS, and R.GS indicate the analysis results of RNA-seq data in FS, DS and GS, respectively. [file peerj-10-13428-s008.pdf]

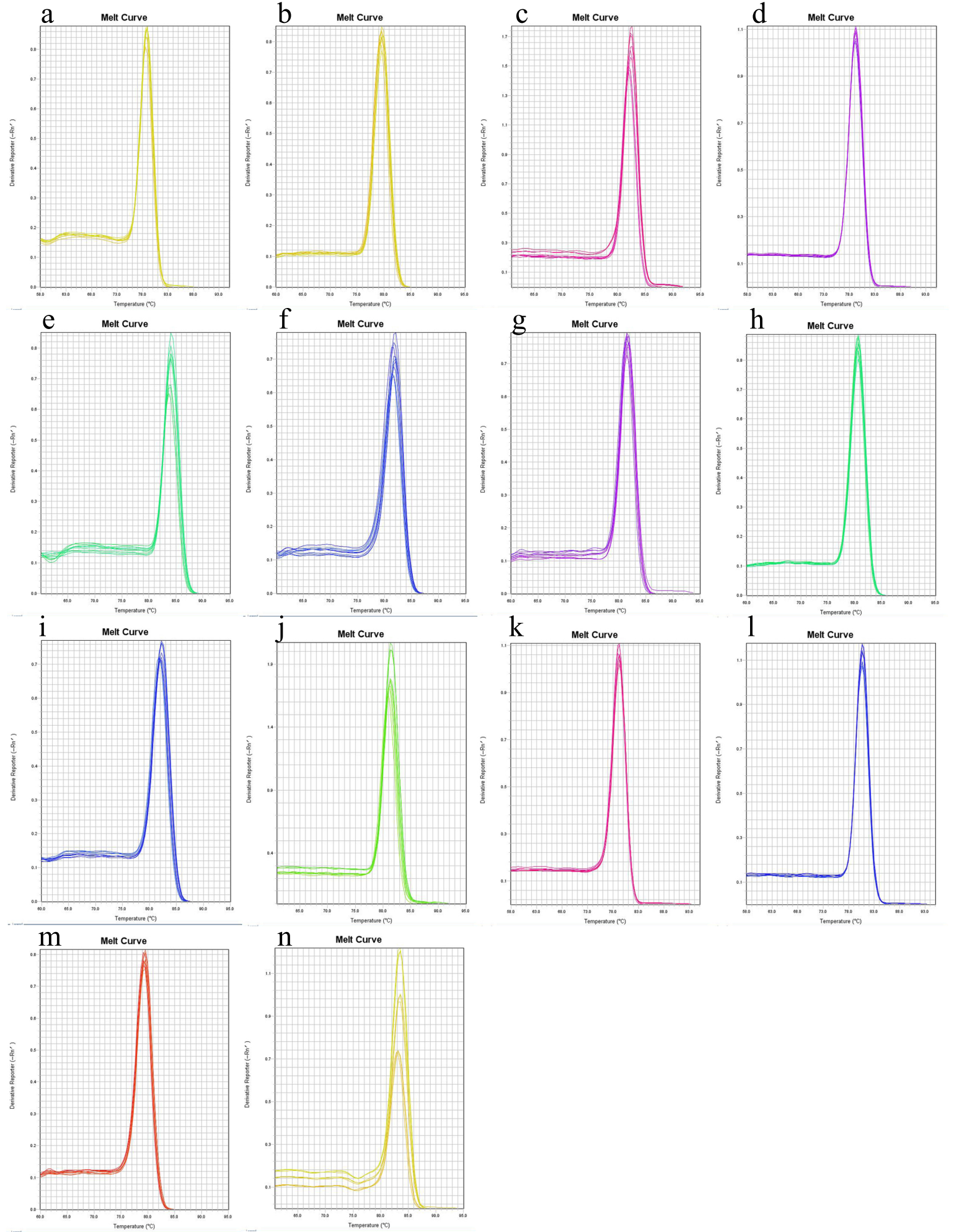

Supplement: Supplemental Information 10 — Melting curve analysis was performed during the qRT-PCR precedure. The letter a-n respectively indicate the analysis results of AhXTH4, AhXTH5, AhXTH14, AhXTH15, AhXTH16, AhXTH24, AhXTH30, AhXTH31, AhXTH35, AhXTH38, AhXTH42, AhXTH52, ACTIN and UBI. [file peerj-10-13428-s010.pdf]

2K

1

2K

2

2000bp

750bp

2000bp

750bp

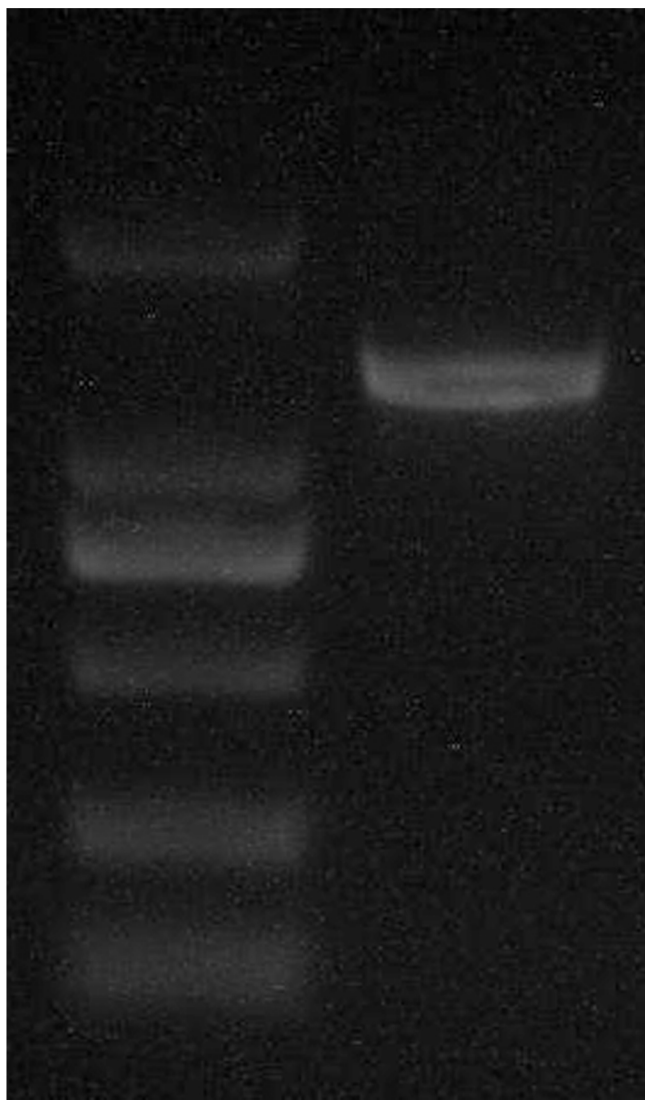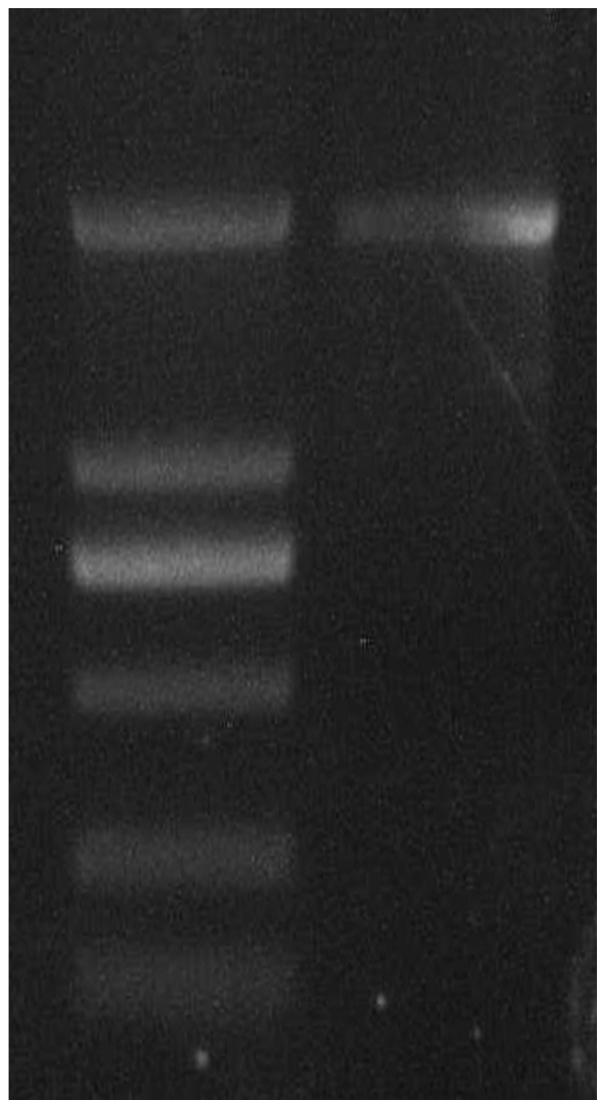

Supplement: Supplemental Information 11 — 2K: DNA molecular marker (TaKaRa DL2000); 1 and 2: PCR products of the promoters of AhXTH4 and AhXTH22 [file peerj-10-13428-s011.pdf]
